# Time Trends and Predictions of Suicide Mortality for People Aged 70 Years and Over From 1990 to 2030 Based on the Global Burden of Disease Study 2017

**DOI:** 10.3389/fpsyt.2021.721343

**Published:** 2021-09-27

**Authors:** Jun He, Feiyun Ouyang, Dan Qiu, Ling Li, Yilu Li, Shuiyuan Xiao

**Affiliations:** ^1^Department of Social Medicine and Health Management, Xiangya School of Public Health, Central South University, Changsha, China; ^2^Hunan Provincial Key Laboratory of Clinical Epidemiology, Changsha, China

**Keywords:** suicide, the elderly, mortality, prediction, global burden of disease study, socio-demographic index

## Abstract

**Background:** High suicide rate in the elderly is an important global public health problem but has not received the attention it deserves. This study aimed to examine time trends of suicide mortality for people aged 70 years and over by sex, age, and location from 1990 to 2017, and to provide predictions up to 2030.

**Methods:** Using data from the Global Burden of Disease study 2017, we presented elderly suicide mortality changes and compared the patterns for the elderly with that for all ages. We estimated associations between socio-demographic index (SDI) and suicide mortality rates using a restricted cubic spline smoother, and predicted suicide mortality rates up to 2030.

**Results:** In 2017, 118,813 people aged 70 years and over died from suicide, indicating a mortality rate of 27.5 per 100,000, with the highest rates in Eastern Sub-Saharan Africa, Western Sub-Saharan Africa, and Central Sub-Saharan Africa, and for countries and territories, the highest were in South Korea, Zimbabwe, Lesotho, Mozambique, and Senegal. Between 1990 and 2017, suicide mortality rate for the elderly aged 70 years and over decreased globally (percentage change −29.1%), and the largest decreases occurred in East Asia, Southern Latin America, and Western Europe. Nationally, the largest decrease was found in Chile, followed by Czech Republic, Hungary, Turkey, and Philippines. For most countries, the elderly mortality rate was higher than the age-standardized rate, with the largest percentage differences in China and countries in Sub-Saharan Africa. The elderly suicide mortality rate decreased as SDI increased, except for a slight rebound at mid to high SDI. According to projections, 10 out of 195 countries were expected to meet the SDGs indicator of a third reduction by 2030.

**Conclusions:** Variability in suicide mortality rates for the elderly aged 70 years and over by sex, age, region, country, and SDI can guide preventive policies, but causes of the variability need further study. Comprehensive strategies should be adopted to reduce suicide rates and close the gap to the 2030 SDGs.

## Introduction

Suicide is a serious global public health issue. In 2017, it was estimated that 794,000 people died from suicide worldwide, accounting for 1.4% of total deaths, with a global age-standardized mortality rate of 10.0 per 100,000. Suicide caused 33,577,000 years of life lost (YLLs) and was the 14th leading cause of YLLs in 2017 (1). In most countries of the world, suicide rates increase with age and are particularly high among the elderly, especially those aged 70 and older (2, 3). In the context of increasing life expectancy, falling birth rates, and rapid population aging globally, suicide deaths for the elderly should be of particular concern (4). However, they have not received the same level of attention as youth and young adults in suicide prevention (5).

Suicide rates for the elderly vary considerably by sex, region, and country. Around the year 2000, for people aged 65 and older, the suicide rate was reported to be higher among males than females, with the highest rates in Central and Eastern European, some East Asia, and some West European countries (6). Many factors can account for increased elderly suicide risk. Factors associated with the social and interpersonal relationships encompass early life events such as sexual abuse, social exclusion and lack of social support, economic insecurity, access to lethal means, stressful life events, and traumatic life experiences. Individual risk factors include major depression and other mental disorders, physical illnesses and functional impairments, personality traits such as neuroticism, hopelessness, decision making and cognitive inhibition, and some other neurobiological factors (4, 5, 7). At the national level, a number of health-related indicators, markers of socioeconomic status and health care are correlated with suicide rates for the elderly. Previous studies, using data before 2007, have shown a positive relationship of elderly suicide rates with per capita expenditures on health, life expectancy, and elderly dependency ratios (8, 9), and a U-shaped curve with fertility rates, average annual population growth rates, and adult literacy rates (10–12).

Given the great heterogeneity of suicide among different groups of the elderly (13, 14), it is necessary to compare suicide characteristics across countries and to understand trends over time for suggesting recommendations on suicide prevention. Several cross-national studies have examined differences and trends in elderly suicide rates by age, sex, and region using data from WHO (6, 15); however, they were conducted more than a decade ago and covered only a few dozen countries. Suicide is an indicator of the UN Sustainable Development Goals (SDGs), in which age-standardized mortality rate due to suicide is supposed to reduce by one third from 2015 to 2030 (16). Measuring changes in suicide mortality rates among older people is important to assess attainment of the SDGs. However, reliable global data on suicide are difficult to obtain because of the stigma, inconsistent definition of suicide, and poor registration facilities in specific countries (17). The Global Burden of Disease Study (GBD), which estimates global, regional, national mortality through data processing, gives us a chance to show the situation of elderly suicide worldwide. Therefore, the aims of this study are to (1) report the patterns of elderly mortality from suicide globally, for 21 GBD regions, and 195 countries and territories in 2017; (2) describe their changes between 1990 and 2017; and (3) project the trends up to 2030, using data from GBD 2017.

## Methods

### Data Sources

This study used data freely available online from GBD 2017 (18), whose methods and results were described in detail elsewhere (1, 19, 20). In summary, the GBD study is a comprehensive worldwide observational epidemiological study which describes mortality and morbidity from major diseases, injuries, and risk factors to health by location, age, and sex. In GBD 2017, mortality from 282 causes were estimated by location, age, and sex from 1990 to 2017 for 195 countries and territories, and the Ninth and Tenth Revisions of the International Classification of Diseases (ICD-9, codes E950-E959; ICD-10, codes X60-X64.9, X66-X84.9, Y87.0) were used to define suicide mortality (21). The causes of death data were mainly estimated from vital registration systems, verbal autopsy reports, registry, survey, police, and surveillance data, with adjustments made for their deficiencies and incompleteness (1). Garbage codes that were not possible causes of death, not specific underlying causes of death, or not informative underlying causes of death were redistributed by age, sex, location, and year to the most likely causes of death, using regression models, redistribution based on fixed proportions, proportional reassignment, and fractional assignment of a death assigned to multiple causes (1). Age-standardized rates of suicide mortality for all ages were calculated using the GBD world population age standard (20). Point estimates for metrics were derived from the mean of 1,000 draws from the posterior distribution of modeled suicide mortality by age, sex, and location, and their 95% uncertainty intervals (UIs) were estimated using the 2.5th and 97.5th percentiles of these 1,000 draws (1).

### Data Presentation

The present study was targeted at the elderly aged 70 years and over (hereafter referred to as the elderly) and included five age groups: 70 to 74, 75 to 79, 80 to 84, 85 to 89, 90 to 94, and 95 plus years. We presented death numbers, mortality rates, and mortality fractions from suicide for the elderly by sex, age, and location, and described their percentage changes between 1990 and 2017. Mortality fraction from suicide refers to the proportion of deaths from suicide to deaths from all causes. Percentage changes were considered statistically significant if the 95% UI did not include zero. To illustrate how the elderly differ from the general population, we compared suicide mortality rates for the elderly with age-standardized suicide mortality rates for all ages by calculating their percentage differences. And we compared the decline in mortality rates for the elderly and for the general population between 1990 and 2017 by calculating the differences of their percentage changes. The corresponding formulas are detailed in the Supplementary Tables 2, 4. All analyses and figures in this paper were performed with R (version 4.0.5).

### Socio-Demographic Index and Mortality Analysis

Socio-demographic index (SDI) is a composite indicator of sociodemographic development status strongly correlated with health outcomes, which was developed for GBD study. It is the geometric mean of a location's three re-scaled components: total fertility rate under age 25, mean years of education for those aged 15 and older, and lag distributed income per capita. SDI ranges from 0 to 1, with 0 representing minimum level of development relevant to health, while 1 representing maximum level (22). We used a linear regression model with a restricted cubic spline function on SDI to visualize the relationship between SDI and suicide mortality rates, weighted for the elderly population (Supplementary S1) (23). The model was fitted by the “ols” function from the R package “rms version 6.0–1,” with 5 knots at the 5, 27.5, 50, 72.5, and 95th percentiles of SDI. In sensitivity analyses, We assessed associations of the mortality rate with fertility rate, GDP per capital, and secondary school enrollment, with data from The World Bank (Supplementary S2) (24). To assess the robustness of the restricted cubic spline function, we refitted the model after removing the country with the largest mortality rate, and compared the result with a local polynomial regression fitting, using the “loess” function from the R package “stats version 4.0.5” with the span of 0.75 (Supplementary S1).

### Prediction Model

As an indicator of the SDGs, age-standardized mortality rate due to suicide should reduce by one third from 2015 to 2030 (16). While there is no specific indicator for people aged 70 and older, the one-third reduction should be followed by the elderly, as they had the highest suicide mortality rate among all ages. In order to evaluate the probability of meeting the indicator, we used Autoregressive Integrated Moving Average (ARIMA) model to predict the suicide mortality rates for the elderly among each location and each sex up to 2030, based on the mean mortality rates from 1990 to 2017 (Supplementary S1). ARIMA model is one of the most common time series analysis methods used to predict future series that is based on its own past information (25). The model is generally denoted as ARIMA (*p, d, q*) where *p, d*, and *q* refer to the order of autoregression, difference, and moving average, respectively. In this study, parameters of the models for forecasting global, regional, and national suicide mortality rates were provided by the “auto.arima” function from the R package “forecast version 8.12” (26). Ljung-Box test was performed to check the autocorrelation of the model residuals, and *P* > 0.05 indicated that the residuals were white noise. To tested the predictive ability, we used the data from 1990 to 2010 to predict the mortality rates from 2011 to 2017 and calculated the relatively mean absolute error (RMAE).

## Results

### Suicide Mortality in 2017

**Figure 1:**
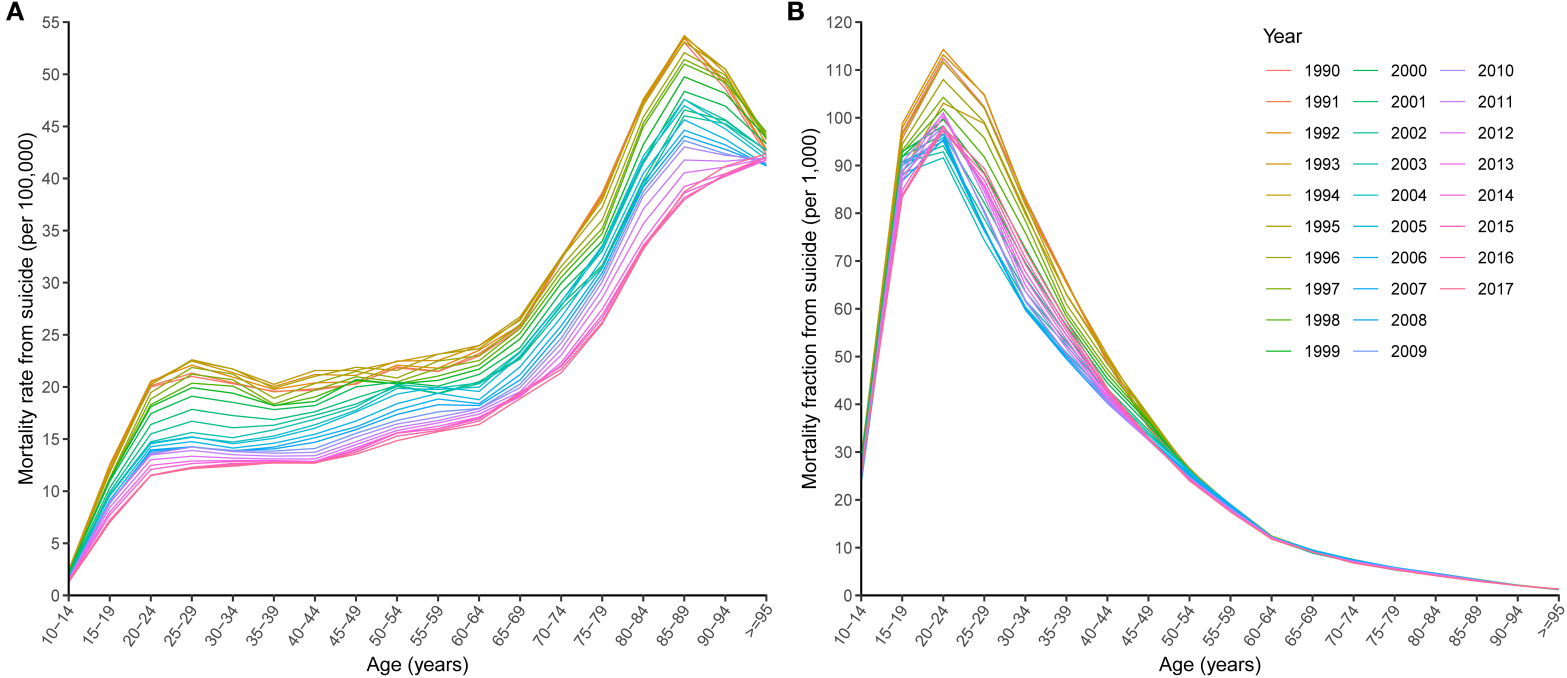
Age-specific mortality rates **(A)** and mortality fractions **(B)** from suicide by year.

**Figure 2:**
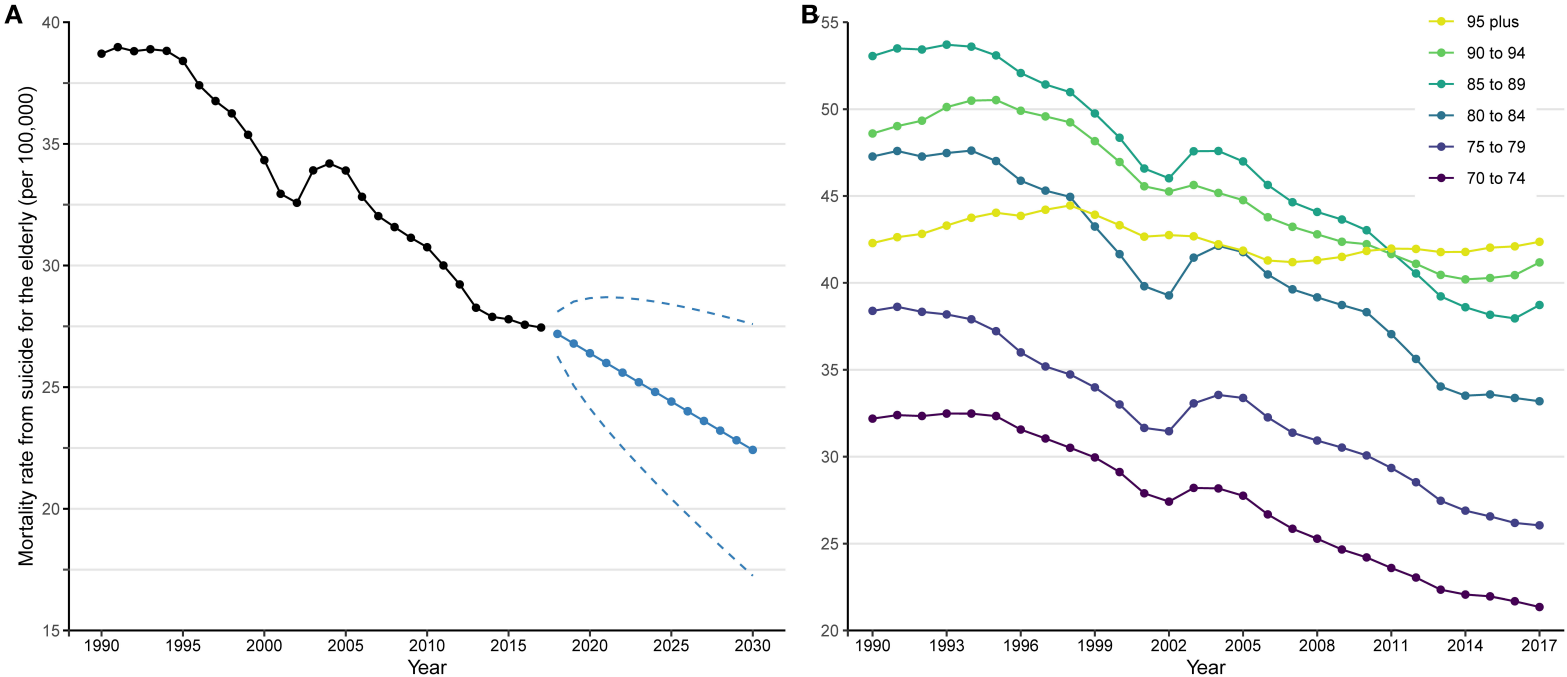
Suicide mortality rates for the elderly from 1990 to 2017 and prediction up to 2030. **(A)** Trend and prediction of global mortality rate; **(B)** trends of mortality rates by age group. In **(A)**, black points and solid line denote suicide mortality rates for the elderly from 1990 to 2017 estimated by Global Burden of Disease study 2017. Blue points and solid line denote suicide mortality rates for the elderly from 2018 to 2030 projected by Autoregressive Integrated Moving Average model based on the mean rates from 1990 to 2017. Blue dashed lines denote 95% confidence intervals of the projected suicide mortality rates.

Globally, an estimated 118,813 (95% UI 111,805 to 123,135) elderly people died from suicide in 2017, accounting for 15.0% (95% UI 14.1 to 15.5%) of suicide deaths for all ages, while the elderly made up only 5.7% (95% UI 5.5 to 5.8%) of the total population. The suicide mortality rate for the elderly (27.5 per 100,000, 95% UI 25.8 to 28.4) was the highest of all ages in 2017, and itself increased with age group (Figures 1A, 2). However, the suicide mortality fraction for the elderly (4.4‰, 95% UI 4.1 to 4.5‰) was the lowest (Figure 1B). For sex difference, the rate was higher for males (39.9 per 100,000, 95% UI 36.3 to 41.7) than for females (17.8 per 100,000, 95% UI 17.0 to 18.4).

**Figure 3:**
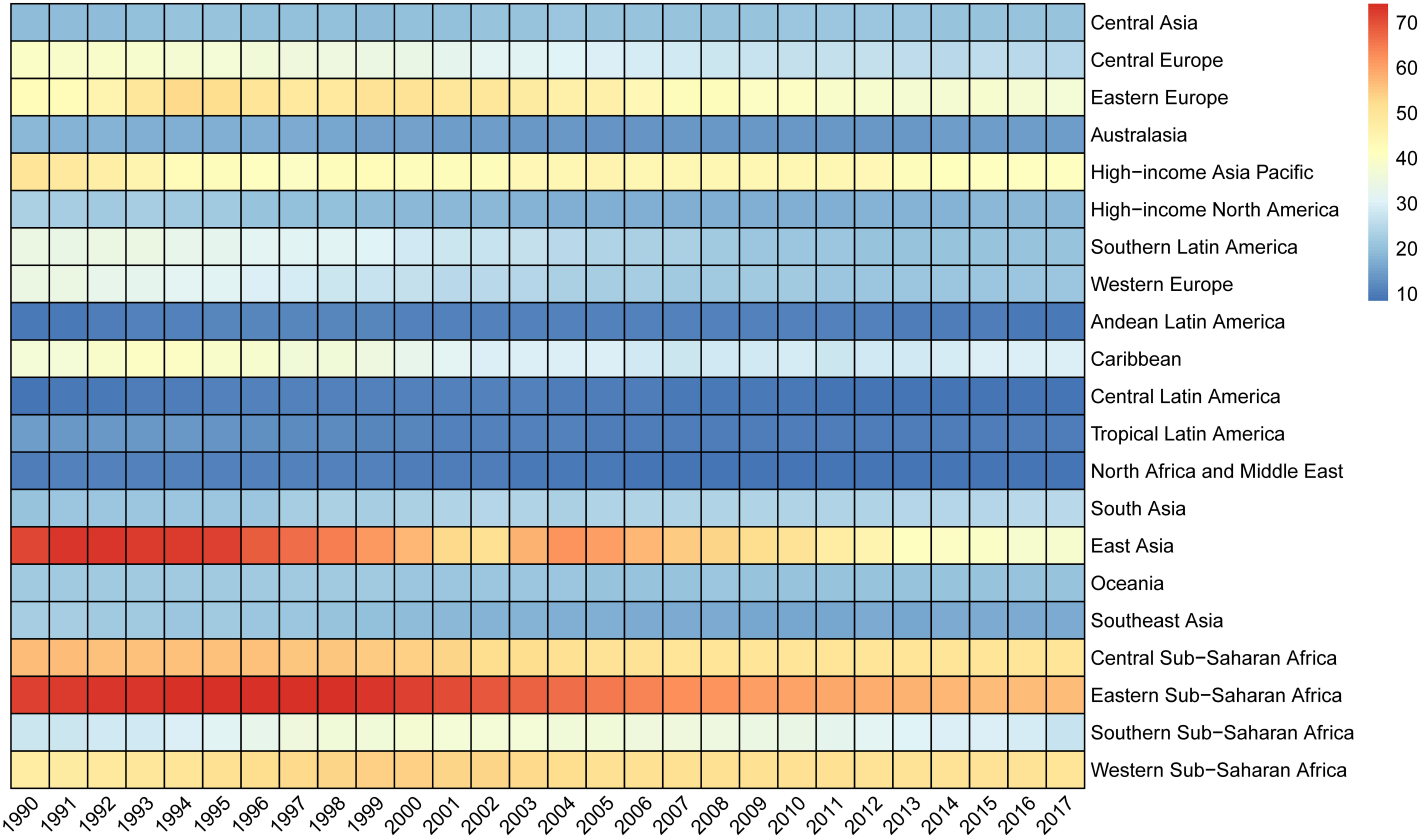
Mortality rates per 100,000 from suicide for the elderly by GBD region, 1990 to 2017. GBD, Global Burden of Disease.

**Figure 4:**
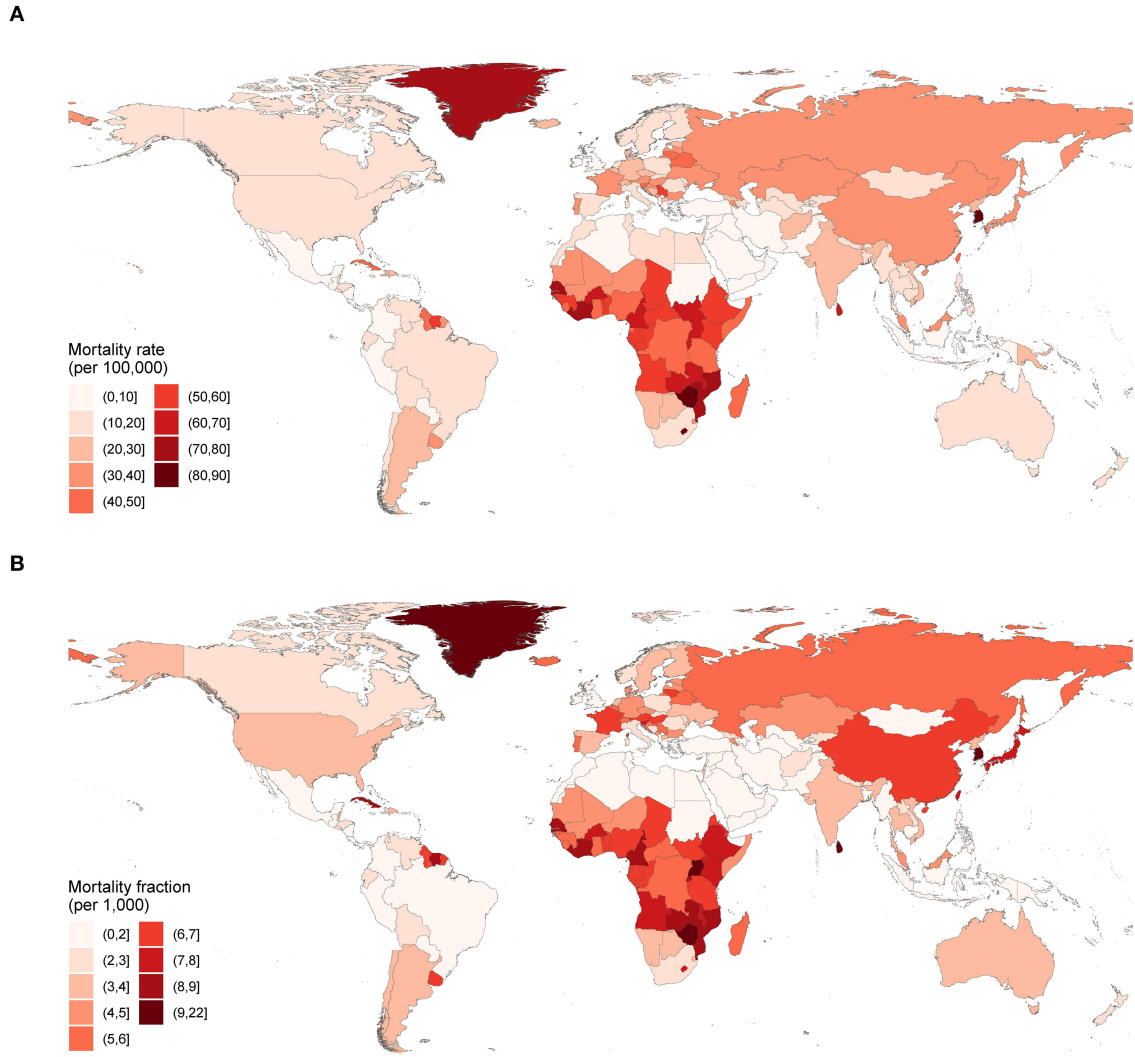
Mortality rates **(A)** and mortality fractions **(B)** from suicide for the elderly in 2017.

Table 1; Figure 3 show suicide mortality for the elderly among 21 GBD defined regions. In 2017, the mortality rates were highest in Eastern Sub-Saharan Africa, Western Sub-Saharan Africa, and Central Sub-Saharan Africa, and lowest in North Africa and Middle East, Central Latin America, and Andean Latin America. While the mortality fractions were highest in High-income Asia Pacific, Eastern Sub-Saharan Africa, and Western Sub-Saharan Africa, and lowest in North Africa and Middle East, Central Latin America, and Tropical Latin America (Table 1). Substantial variability existed in the mortality rates of 195 countries and territories. Figure 4A shows that in 2017, for countries with populations more than 1,000,000, the highest mortality rates were in South Korea, Zimbabwe, Lesotho, Mozambique, and Senegal, while the lowest were in Kuwait, Turkey, Pakistan, Cyprus, and Jamaica. For mortality fraction, the highest were in South Korea, Sri Lanka, Zimbabwe, Uganda, and Senegal, and the lowest were in Pakistan, Jamaica, Syria, Tajikistan, and Indonesia (Supplementary Table 1; Figure 4B).

**Table 1:** Death numbers, mortality rates, and mortality fractions from suicide for people aged 70 and older, and their percentage changes between 1990 and 2017, by sex and GBD region.

	**Death number (95% UI)**			**Mortality rate per 100,000 (95% UI)**			**Mortality fraction per 1,000 (95% UI)**		
	**1990**	**2017**	**Percentage change (%)**	**1990**	**2017**	**Percentage change (%)**	**1990**	**2017**	**Percentage change (%)**
Global	78,699 (71,706, 82,375)	118,813 (111,805, 123,135)	51.0 (42.2, 61.9)	38.7 (35.3, 40.5)	27.5 (25.8, 28.4)	−29.1 (−33.2, −24.0)	5.1 (4.6, 5.3)	4.4 (4.1, 4.5)	−13.7 (−18.7, −7.6)
Female	34,352 (33,105, 36,384)	43,264 (41,494, 44,857)	25.9 (16.0, 33.2)	28.6 (27.6, 30.3)	17.8 (17.0, 18.4)	−37.9 (−42.8, −34.3)	4.1 (3.9, 4.3)	3.1 (2.9, 3.2)	−25.3 (−31.1, −21.3)
Male	44,346 (36,504, 47,351)	75,548 (68,749, 79,073)	70.4 (54.3, 98.2)	53.2 (43.8, 56.8)	39.9 (36.3, 41.7)	−25.1 (−32.2, −12.9)	6.2 (5.1, 6.6)	5.8 (5.3, 6.0)	−6.6 (−15.0, 8.7)
Central Asia	428 (412, 446)	617 (582, 649)	44.0 (35.3, 53.6)	19.3 (18.6, 20.1)	20.3 (19.2, 21.4)	5.1 (−1.2, 12.1)	2.4 (2.4, 2.5)	2.3 (2.1, 2.4)	−6.7 (−12.5, −0.4)
Central Europe	3,136 (3,037, 3,213)	3,279 (3,146, 3,417)	4.6 (−0.6, 9.7)	39.8 (38.6, 40.8)	24.7 (23.7, 25.8)	−37.9 (−41.0, −34.9)	4.5 (4.3, 4.6)	3.7 (3.5, 3.8)	−17.9 (−21.8 , −14.1)
Eastern Europe	6,491 (6,375, 6,651)	7,265 (6,988, 7,468)	11.9 (7.3, 15.6)	42.7 (42.0, 43.8)	37.1 (35.7, 38.2)	−13.1 (−16.7, −10.2)	5.2 (5.1, 5.3)	4.7 (4.6, 4.9)	−8.3 (−11.9, −5.4)
Australasia	270 (257, 284)	453 (406, 506)	67.7 (48.5, 87.9)	18.5 (17.7, 19.5)	14.8 (13.2, 16.5)	−20.4 (−29.5, −10.8)	2.9 (2.8, 3.1)	3.0 (2.7, 3.2)	1.1 (−7.9, 10.4)
High–income Asia Pacific	5,671 (5,598, 5,746)	12,438 (11,739, 13,010)	119.3 (106.9, 130.3)	50.4 (49.8, 51.1)	40.8 (38.5, 42.7)	−19.2 (−23.8, −15.1)	9.0 (8.8, 9.1)	9.1 (8.7, 9.5)	2.0 (−3.3, 6.4)
High-income North America	5,328 (5,248, 5,428)	6,861 (6,585, 7,103)	28.8 (23.6, 33.6)	22.9 (22.6, 23.4)	18.4 (17.7, 19.1)	−19.5 (−22.8, −16.5)	3.7 (3.6, 3.8)	3.3 (3.2, 3.4)	−9.7 (−13.1, −6.6)
Southern Latin America	901 (867, 935)	1,039 (955, 1,136)	15.4 (4.9, 27.8)	34.3 (33.0, 35.6)	20.9 (19.2, 22.9)	−39.0 (−44.6, −32.5)	4.8 (4.7, 5.0)	3.4 (3.2, 3.7)	−29.6 (−35.0, −23.5)
Western Europe	12,924 (12,692, 13,210)	12,844 (12,194, 13,484)	−0.6 (−5.5, 4.4)	34.6 (34.0, 35.4)	21.2 (20.1, 22.2)	−38.9 (−41.9, −35.7)	4.9 (4.8, 5.0)	3.9 (3.7, 4.1)	−19.6 (−23.2, −16.2)
Andean Latin America	94 (83, 103)	289 (256, 317)	206.0 (168.9, 246.7)	9.3 (8.1, 10.1)	9.7 (8.6, 10.7)	4.4 (−8.2, 18.3)	1.5 (1.3, 1.6)	1.9 (1.7, 2.0)	27.6 (14.5, 41.2)
Caribbean	542 (519, 565)	859 (790, 935)	58.4 (44.2, 74.6)	36.7 (35.1, 38.3)	29.5 (27.1, 32.1)	−19.6 (−26.8, −11.4)	5.5 (5.2, 5.7)	4.9 (4.5, 5.2)	−10.7 (−18.1, −3.0)
Central Latin America	372 (361, 385)	1,071 (1,014, 1,128)	187.9 (167.8, 205.3)	9.1 (8.8, 9.4)	8.7 (8.2, 9.1)	−4.0 (−10.7, 1.8)	1.4 (1.4, 1.5)	1.7 (1.6, 1.7)	18.3 (10.2, 24.9)
Tropical Latin America	628 (610, 648)	1,227 (1,167, 1,271)	95.4 (84.0, 104.8)	14.4 (14.0, 14.9)	10.0 (9.5, 10.4)	−30.8 (−34.8, −27.5)	2.2 (2.1, 2.2)	1.8 (1.7, 1.9)	−15.5 (−20.6, −11.6)
North Africa and Middle East	761 (625, 868)	1,563 (1,360, 1,707)	105.5 (76.8, 136.6)	10.4 (8.5, 11.9)	8.4 (7.4, 9.2)	−18.8 (−30.1, −6.5)	1.4 (1.1, 1.6)	1.4 (1.2, 1.5)	1.2 (−12.1, 16.5)
South Asia	4,806 (3,797, 5,492)	15,756 (12,809, 17,188)	227.9 (176.4, 273.7)	20.8 (16.4, 23.8)	25.0 (20.3, 27.3)	20.3 (1.4, 37.1)	2.2 (1.7, 2.5)	3.3 (2.7, 3.5)	46.9 (24.2, 66.1)
East Asia	28,934 (24,246, 31,188)	39,843 (36,762, 42,268)	37.7 (24.3, 63.3)	71.0 (59.5, 76.6)	38.3 (35.4, 40.7)	−46.1 (−51.3, −36.0)	8.9 (7.4, 9.6)	6.1 (5.7, 6.5)	−31.2 (−37.5, −18.7)
Oceania	20 (16, 25)	42 (36, 50)	110.2 (81.9, 141.3)	22.1 (17.8, 27.1)	20.5 (17.3, 24.1)	−7.4 (−19.9, 6.2)	2.3 (1.8, 2.8)	2.2 (1.8, 2.5)	−4.0 (−16.5, 9.5)
Southeast Asia	2,447 (2,088, 2,666)	4,415 (4,051, 4,749)	80.4 (61.1, 105.9)	22.9 (19.5, 24.9)	16.8 (15.4, 18.1)	−26.5 (−34.4, −16.2)	2.8 (2.4, 3.1)	2.4 (2.2, 2.6)	−14.4 (−23.1, −2.6)
Central Sub–Saharan Africa	458 (346, 549)	884 (675, 1,032)	93.1 (54.4, 138.0)	56.9 (43.0, 68.2)	50.1 (38.3, 58.6)	−11.9 (−29.6, 8.5)	5.7 (4.3, 6.8)	5.8 (4.5, 6.7)	0.8 (−18.4, 22.0)
Eastern Sub–Saharan Africa	2,171 (1,869, 2,475)	3,593 (3,241, 4,106)	65.5 (39.1, 90.7)	71.9 (61.9, 82.0)	56.6 (51.0, 64.7)	−21.3 (−33.8, −9.3)	7.0 (6.0, 7.9)	7.4 (6.7, 8.4)	6.9 (−9.4, 22.5)
Southern Sub–Saharan Africa	366 (319, 419)	699 (633, 759)	91.1 (61.3, 117.1)	27.6 (24.0, 31.6)	27.4 (24.8, 29.7)	−0.8 (−16.2, 12.8)	4.3 (3.7, 4.9)	4.0 (3.7, 4.4)	−6.4 (−20.2, 5.2)
Western Sub–Saharan Africa	1,952 (1,592, 2,392)	3,774 (3,168, 4,532)	93.4 (58.4, 134.8)	47.7 (38.9, 58.5)	50.5 (42.4, 60.6)	5.7 (−13.4, 28.3)	5.3 (4.5, 6.3)	6.6 (5.6, 7.5)	24.4 (4.8, 42.4)

*GBD, Global Burden of Disease; UI, Uncertainty interval*.

### Changes Between GBD 1990 and GBD 2017

Between 1990 and 2017, the suicide deaths number for the elderly increased globally (percentage change 51.0%, 95% UI 42.2 to 61.9%) (Table 1). However, the suicide mortality rate (percentage change −29.1%, 95% UI −33.2% to −24.0%) and suicide mortality fraction (percentage change −13.7%, 95% UI −18.7% to −7.6%) showed overall decreasing trends (Table 1; Figures 1, 2A). Males experienced a slower relative decrease in the suicide mortality rate (percentage change −25.1%, 95% UI −32.2% to −12.9%) than females (−37.9%, 95% UI −42.8% to −34.3%). Figure 2B shows that the age specific mortality rates from suicide decreased between 1990 and 2017, excepted for the 95 plus age group.

**Figure 5:**
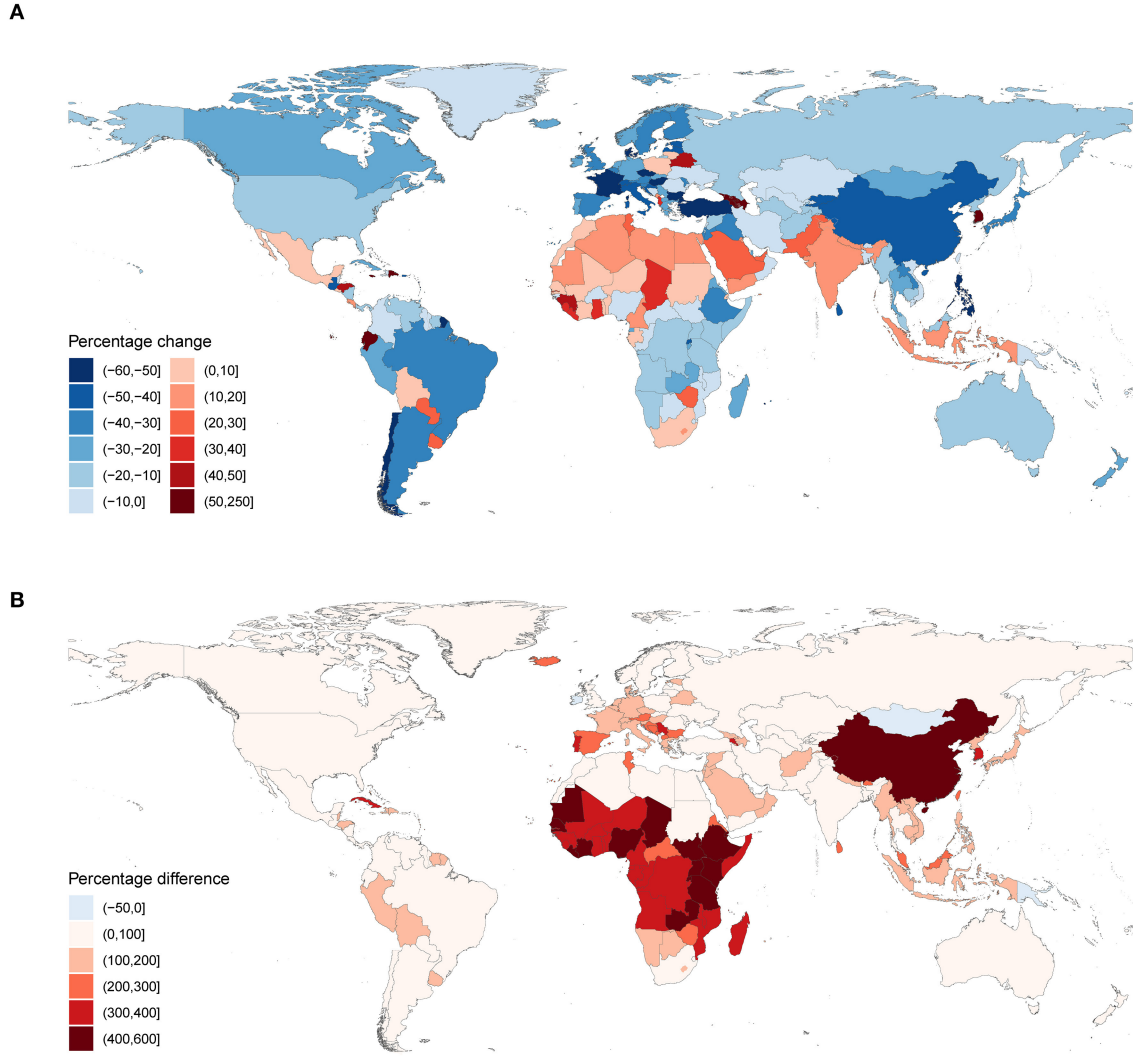
Percentage changes and percentage differences of mortality rates from suicide. **(A)** Percentage changes in mortality rates from suicide for the elderly between 1990 and 2017; **(B)** percentage differences between mortality rates from suicide for the elderly and age-standardized mortality rates from suicide for all ages in 2017. In **(B)**, Percentage difference = 100 * (R_elderly_ - R_std_) / R_std_, where R_elderly_ donates mortality rate from suicide for the elderly, and R_std_ donates age-standardized mortality rate from suicide for all ages.

Table 1; Figure 3 show that 13 of 21 GBD regions had statistically significant decreases (95% UI did not include zero) in the suicide mortality rates between 1990 and 2017, with the largest decreases in East Asia, Southern Latin America, and Western Europe. Yet, a statistically significant increase was observed in South Asia. Nationally, statistically significant decreases occurred in 71 of the 195 countries and territories (Supplementary Table 1). Figure 5A shows that the largest decreases were found in Chile, followed by Czech Republic, Hungary, Turkey, and Philippines. However, there were 15 countries and territories where the suicide mortality rates increased significantly, with the largest in Armenia, South Korea, Ecuador, Dominican Republic, and Jamaica. For suicide mortality fraction, the three regions with the largest decreases were consistent with the mortality rate. The countries and territories with the largest decreases were Philippines, Chile, Bulgaria, Hungary, and Turkey (Supplementary Table 1).

### Differences Between the Elderly and the General Population

In 2017, the global suicide mortality rate for the elderly (27.5 per 100,000, 95% UI 25.8 to 28.4) was almost three times the age-standardized suicide mortality rate for all ages (10.0 per 100,000, 95% UI 9.4 to 10.3). In most regions and countries, the elderly rates were higher than the age-standardized rates, except for four countries: Mongolia, Ireland, Papua New Guinea, and Kiribati (Supplementary Tables 2, 3). Figure 5B shows that in 2017, China and countries in Sub-Saharan Africa had the largest percentage differences between the rates for the elderly and that for all ages. From 1990 to 2017, global suicide mortality rate decreased for both the elderly (percentage change −29.1%, 95% UI −33.2% to −24.0%) and all ages (−35.4%, 95% UI −39.5% to −30.4%); however, the elderly rate fell relatively more slowly. This difference in the percentage decrease was most notable in several Asia and Southern Sub-Saharan Africa countries (Supplementary Tables 4, 5; Supplementary Figure 1).

### Associations Between SDI and Mortality Rates

**Figure 6:**
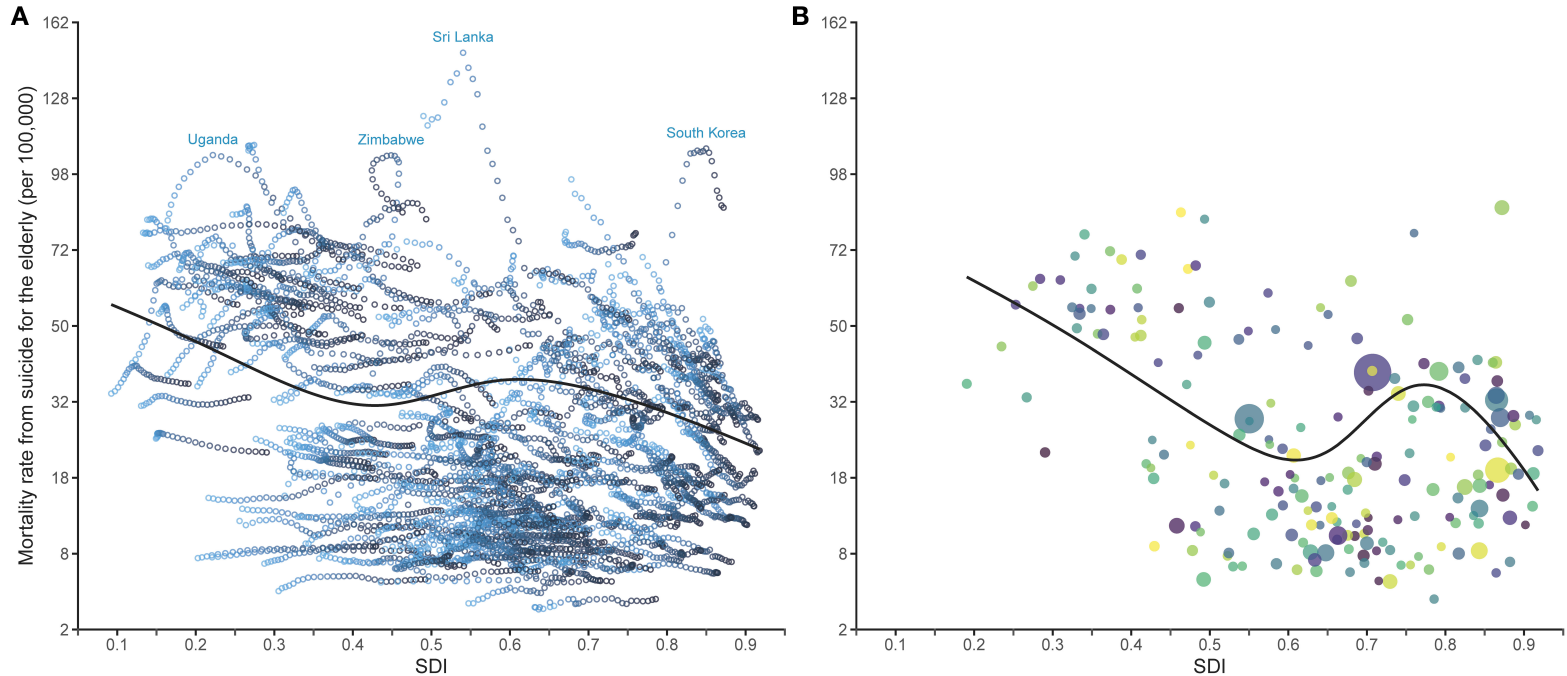
Relationship between SDI and elderly suicide mortality rates from 1990 to 2017 **(A)** and in 2017 **(B)** for 195 countries and territories. SDI, Socio-demographic Index. Models are weighted by the elderly population. Y axes are shown after the square root transformation. The black curves represent the mortality rates expected on the basis of SDI alone, with linear model and restricted cubic spline smoother which includes 5 knots at the 5, 27.5, 50, 72.5, and 95th percentiles. In **(A)**, each hollow point corresponds to the mortality rate for a country or territory in a specific year. For a given location, the gradient colors from light to dark indicate the years from 1990 to 2017. Uganda, Zimbabwe, Sri Lanka, and South Korea were four countries with high suicide mortality rates whose temporal trends showed inverted U-shape curves. In **(B)**, each point of a specific color represents one country or territory, and the point size represents the elderly population.

Association between SDI and the suicide mortality rates for 195 countries and territories from 1990 to 2017 is illustrated in Figure 6A. In general, it turned out to be an overall decreasing pattern (*P* < 0.001). The mortality rate decreased obviously at first, and then increased at SDI of about 0.4 to 0.6, followed by a continuous decrease again. For each nation, the mortality rate decreased as SDI rose over time, with some exceptions. For example, in countries where the mortality rates were notably higher than the expected, the trends were more likely to be non-monotonic. A few of them showed an inverted U-shaped curve, such as Uganda, Sri Lanka, and South Korea. For Zimbabwe, there was even a decade of decline in SDI. Figure 6B shows the association in 2017 with a similar shape of curve but compressed to higher SDI. For each year, the relationship suggested an increasing downward trend with a rebound at mid to high SDI (Supplementary Figure 2).

### Predictions for Mortality Rates Up to 2030

Figure 2A shows time trend in global suicide mortality rate for the elderly from 1990 to 2017 and the predictions up to 2030. The suicide mortality rate was supposed to decline continually for the next 13 years, to 22.4 per 100,000 in 2030. This represented a percentage change of −19.3% from 27.8 per 100,000 in 2015, yet less than a third. The mortality rate for females was predicted to decrease (percentage change −29.5%, from 18.3 per 100,000 in 2015 to 12.9 per 100,000 in 2030) almost twice as fast as for males (percentage change −15.6%, from 40.1 per 100,000 in 2015 to 33.8 per 100,000 in 2030). Of 21 GBD regions, 12 may experience a decline in the mortality rates, with East Asia (percentage change −41.2%) falling by more than one-third. In most countries and territories, the mortality rates were projected to decline by 2030, with 10 countries achieving a one-third reduction (Supplementary Table 6; Supplementary Figures 3, 4).

### Sensitivity Analyses

South Korea had much higher mortality rates than other high SDI countries and territories; however, after excluding this country, the relationship between SDI and the mortality rate remained a similar downward trend. The curve fitted by local polynomial regression did not change substantially compared to the restricted cubic spline curve (Supplementary Figure 5). The associations of the mortality rate with fertility rate, GDP per capital, and secondary school enrollment are shown in Supplementary Figure 6 as U-shaped, basically declining, and decreasing, respectively. Globally, test of predictive ability for ARIMA model showed a RMAE of 3.3% and all *P*-values of Ljung-Box test were >0.05. Nationally, the RMAE was <10% for 163 out of 195 countries and territories.

## Discussion

In this study, we analyzed patterns and changes of elderly suicide mortality by sex, age, and location using data from GBD 2017. There have been several studies on suicide among the elderly worldwide. In 2016 and before, dozens of studies were conducted by Shah et al. to examine patterns and trends in suicide for the elderly by sex, age, and location and to investigate their correlates widely, mostly using suicide data from WHO (3, 6, 8, 9, 15, 17, 27–31). Recently, several studies reviewed the risk factors, prevention, and control strategies for suicide among the elderly (5, 7). Compared to these previous studies, we included many more countries and territories with a longer span of trends. We also analyzed the differences between the elderly and all ages, estimated the associations between the composite indicator, SDI, and suicide mortality rates, and made projections of future trends. The results showed that globally, the elderly had the highest suicide mortality rate among all age groups, consistent with their being at high mortality risk from all causes combined, though the suicide mortality fraction was much lower than younger age groups. From 1990 to 2017, the number of suicide deaths increased substantially, largely due to aging and higher suicide rate for the elderly. The suicide mortality rate, however, decreased during this period, reflecting a combination of the decline in all-cause mortality rate and the decline in the suicide mortality fraction (18). Several reasons explain the decrease, including global economic and social developments, and improved health services, especially treatments for depressive disorders (32).

The elderly mortality rate was higher for males than for females in 2017, which was the same as age-standardized rate for all ages, but for suicide attempt rate, it was reversed (33). The main reason for this discrepancy was that males tended to use highly lethal methods more than females in suicidal behavior, and even in the same methods, males were more likely to succeed because they had a stronger intention to die (34). Changes in suicide mortality rates from 1990 to 2017 varied by age, with the younger elderly declining faster than the older elderly. Consequently, the suicide mortality rate in 2017 increased with age and was highest in people aged 95 and over. The gap between age groups may be due to physical diseases and bereavement increasing with age, and is expected to be even greater in the future (14). The older the person, the more likely they are to become widowed, to be out of the work force, and to choose a lethal way of suicide such as hanging, but the less likely they are to receive psychiatric services before committing suicide (13, 14). With increased longevity and aging globally, a higher proportion of the elderly population increases the burden on health care and social security in a specific country, and a stronger feeling of loneliness and isolation in the elderly can lead to more mental disorders like depression (35, 36). This phenomenon may be more serious in the older elderly, which can offset the benefits from social and economic developments, resulting their slower declining trends in suicide mortality rates.

Regional imbalances of suicide mortality for the elderly existed in suicide mortality rates, gaps between elderly and all ages, and their changes over time. In general, potential explanations for these imbalances include methodological issues of data acquisition, genetic factors, differences in mental disorders (especially depression), differences in life expectancy, socioeconomic deprivation, social fragmentation, availability of health care services, public health policies, and cultural factors (6). In 2017, of 21 GBD regions, the highest elderly suicide mortality rates were estimated for Eastern Sub-Saharan Africa, Western Sub-Saharan Africa, and Central Sub-Saharan Africa, but for the age-standardized suicide mortality rate for all ages, Sub-Saharan Africa regions were much lower and not the leading regions anymore (37). Indeed, Sub-Saharan Africa is one of regions with the largest disparities between suicide mortality rates for the elderly and all ages, as the suicide mortality rates for the elderly decreased more slowly than the general population. The three Sub-Saharan Africa regions all ranked among the top of 21 GBD regions in terms of suicide mortality fractions for the elderly, suggesting that their higher suicide mortality rates for the elderly were also a reflection of the higher suicide mortality proportions for the elderly in these regions. Sub-Saharan Africa is the world's poorest and youngest region, with only 17% of the elderly in receipt of pension in 2010/12, the lowest in the world (38). They become poorer as they age and face a heavy disease burden, particularly from chronic disease. However, most older people used health services far less than younger people did because of the unavailability, age insensitivity, or high costs of the services (39). Currently, mental health issues are becoming increasingly important in Africa, but there has a large treatment gap for psychiatric care services in the continent, and the health budgets for psychiatric care in most countries remain very low (40). Admittedly, there is considerable uncertainty in all-cause mortality and limited evidence of cause-specific mortality fractions in Sub-Saharan Africa due to the absence of full-fledged vital registration systems. For example, a study based on verbal autopsies did not show excess suicide mortality for the elderly in several sites of Sub-Saharan Africa (41). Moreover, research on mental health in Africa is rare and few articles were published on suicide, resulting in limited knowledge on this field (42, 43). The scarcity of research reflects the lack of attention paid to the issue by Africans and governments.

Nationally, South Korea had the highest suicide mortality rate for the elderly in 2017, and the rate has tripled from 1990 to 2017. In 1998, the Asian financial crisis triggered a sudden rise in South Korea's suicide, which gradually stabilized in 2005 and then began to decline in 2010. The sharp increase in suicide mortality before 2005 might be mainly due to social issues caused by the economic recession, while much of the subsequent improvement could be attributed to the establishment of the National Strategy for Suicide Prevention in 2004 and follow-up policies, the increase in welfare facilities for the elderly after 2005, and the paraquat prohibition in 2011 by the government (44–47). Poverty in the elderly may also play a role in the high suicide mortality rate, with their relative poverty rates in South Korea exceeding 40% and the highest among the Organization for Economic Co-operation and Development member countries (48). Another reason for the high suicide rate in South Korea is the dominant method of hanging, which is highly lethal and easy to access, whereas pesticide poisoning and jumping also account for a large proportion (49, 50). In contrast to South Korea, the elderly in Japan and Singapore, both High-income Asia Pacific countries, were much less affected by the financial crisis. Over 28 years, the age-standardized suicide mortality rate in Japan has increased slightly, but the rate for the elderly has experienced a significant decline, with the result that the gap between them is narrowing (18).

Before South Korea, the countries with the highest suicide mortality rates for the elderly were Sri Lanka (1990–2002) and Zimbabwe (2003–2004). In Sri Lanka, the suicide mortality rate for the elderly increased dramatically in the 1990 s, to a peak of 148.0 per 100,000 in 1997, and has since fallen sharply. This decline coincided with the introduction (in 1995) of the last of a series bans on WHO class I pesticide starting in 1984, but did not appear to be related to other factors such as unemployment, alcohol misuse, divorce, or Sri Lanka's civil war (1983–2009) (51). Research on suicide rate and its risk factors in Zimbabwe is scarce. The rapid increase in the suicide mortality rate for the elderly prior to 2005 may be attributed to the political instability and the economy hardship arising from the Fast-Track Land Reform Program. In the 2000 s, the suicide mortality rate for the elderly in Zimbabwe remained high, and its SDI even regressed during this period. However, after the country's economy rebounded in 2009, the suicide mortality rate for the elderly began to decline.

China had one of the highest suicide mortality rates in the 1990 s, and it experienced the world's largest decrease in recent decades, with the most marked decrease occurred in young females in rural China (37, 52). The elderly rate declined along with the general population, though the percentage change was slower (53). As a result, China still has a huge gap between suicide mortality rate for the elderly and the general population. While people aged 65 and older comprise about 10% of the Chinese population, they represent almost 40% of suicide deaths each year and the percentage is monotonically increasing (53, 54). Plausible reasons for the dramatic decrease in Chinese suicide mortality rate include rapid economic development, effective control of pesticides and rodenticides, improvement in health care and education, fast urbanization, modernized social values, family planning policy, and media control (55, 56). However, some of these aforementioned reasons also caused suicide among the elderly. For example, economic development and urbanization brought in empty-nesters and isolated the elderly (57). Being left-behind could increase the risk of suicide by leading to depressive symptoms and reducing social support (58). Other risk factors for the high suicide rate among the elderly in China include sociocultural context, inequalities between rural and urban, inadequacy of social welfare and medical services, and significant historical events happened in their youth (52, 54). In China, the disparity in elderly suicide rates between urban and rural area decreased significantly, but the gap still exists (53, 59). This may be a result of the imbalances in economic and social development brought about by the Rural-Urban Dual Society System, for example, the unevenness in education and job opportunities, housing subsidies, health care, social welfare, etc. Suicide methods in China are different from those of other countries. As with the general population, the most common method of suicide among the elderly in China is ingestion of pesticides, compared with firearms in Western countries (59, 60).

From 1990 to 2017, North Africa and Middle East, and Latin America have been the regions with the lowest suicide mortality rates for the elderly, but the underlying mechanisms are not well-characterized. In the Islamic dominated North Africa and Middle East region, the low rate of suicide may be explained by the Muslim concept of life and views on suicide: Islamic tradition regards suicide as a very grave sin, so Muslim seem to be more morally opposed to it than other groups (61, 62). Islam's collective goals, non-self-interested behavior, family society and cohesive communities can also play an important role in this context (63). Moreover, since suicide is considered a criminal offense in many Islamic nations, families do not report the truth of acts for fear of harassment and stigma, which may lead to an underestimation of suicide mortality rate (64, 65). In Latin America region, religion, strong socio-familial ties, and the unreliability of suicide statistics can help explain the relatively low suicide rates (66). Another possible reason is the misclassification of suicide due to legal issues, sociocultural and religious factors, and stigma against suicides (67). The undetermined rates in many Latin America countries were significantly higher than in major developed countries and these deaths were probably suicide, resulting in a high number of underreported suicides in this region (68). Although GBD's methods included reclassification of garbage codes and adjusted partial misclassification where possible, suicide mortality rates may still be underestimated when deaths from suicide were incorrectly recorded as other causes (37). Despite the above assumptions, a great deal of research is needed before we can understand the characteristics of suicide in North Africa-Middle East and Latin America countries.

The present study suggests that the relationship between national SDI and elderly suicide mortality rates was an overall decreasing pattern with a rebound at mid to high SDI countries. Much evidence supported a negative correlation between economic condition and suicide rate, for poorer economy increasing prevalence of major depression, raising rate of unemployment, and reducing the number of health care facilities (69). A study has hypothesized that the association between suicide rate and fertility rate followed a U-shaped curve, which is consistent with our study (10). One possible explanation for this is that rising fertility leads to an increase in youth, who can in turn support the elderly population, but when fertility rate increases beyond a certain point the young compete with the elderly for scarce resources (10, 11). In addition, low levels of education lead to lack of job opportunities, poor problem-solving abilities, and antisocial behavior, which are all risk factors for suicide. The sensitivity analysis of the present study also showed that suicide mortality rate for the elderly declined with increasing secondary school enrollment. In this study, there was a rebound at mid to high SDI, and we found that High-income Asia Pacific, Eastern Europe, Central Europe, and Western Europe have higher suicide mortality rates than middle SDI regions, and their relatively high burden of mental and substance use disorders may have a role in mediating effects (70). In fact, the components of SDI are widely related to other socioeconomic factors, and their impact on suicide needs further study.

In addition to the tremendous emotional costs, suicide has huge economic costs for countries, communities, families, and individuals. A study performed in the United States estimated that the average cost per suicide in 2013 was $243,883 for those aged 65 to 74 and $66,218 for those aged 75 and above (71). The elderly are not “unproductive” as soon as they retire with receiving a pension from the social security. Rapid aging will promote the involvement of older people in productive activities, and there are many unpaid productive activities to realize their contributions, including caring for family members, doing household work, and formal volunteering (72). With increasing emphasis on the dangers of suicide, the UN SDGs include an indicator that age-standardized suicide mortality rate reduces by one third from 2015 to 2030 (16). To achieve the goal, suicide mortality rate for the elderly aged 70 and older, the age group with the highest rate, should reduce by at least one third from 2015 to 2030. However, with a global reduction of 23.8%, only 10 of 195 countries and territories were projected to meet the goal. By comparison, 3% of 118 countries will attain the SDGs indicator of reducing age-standardized suicide mortality rate by one third between 2015 and 2030 (73). There is a wide gap between the reality and SDGs and much more needs to be done in the next decade. Since data validity and reliability are fundamental to suicide research and policy making, it is urgent to strengthen suicide data collection and causes of death registration, especially in low- and middle-income countries in Asia, Africa and South America (17, 74). We need to further identify the genetic and environmental determinants of sex, age, regional, and SDI related variations in elderly suicide mortality rates (75). For countries with high elderly suicide rates, efforts should be made to increase access to aging social services and health care facilities for the elderly and to equip more health professionals with the ability to recognize risk factors of suicide and intervene appropriately (5, 13). Strategies for suicide prevention are critical to elevating prevention of suicide on the political agenda, yet only 38 countries reported having a national strategy by 2020 (76). More countries should be advocated to establish comprehensive national suicide prevention strategies that use a multilevel, multifaceted approach to reduce suicide mortality and accelerate the progress in closing the gap with the SDGs (4).

The general limitations of the GBD data were reported elsewhere (1, 19, 20). This study has several specific limitations. First, all results in this report are based on the estimated data from GBD study, which are considered reliable and comprehensive. However, they are necessarily limited by the quality of the original data, especially in low-income countries without vital registration systems. Global and regional estimates should be viewed cautiously because data are not available from all countries in the world. Second, we cannot distinguish between mortality from suicide and mortality associated with self-harm. Therefore, suicide mortality described in this article includes both intentional and unintentional deaths caused by self-harm. Third, associations between certain characteristics and suicide mortality rates should be interpreted with caution. Since we only analyzed data at the national and regional levels, but not at the individual level, findings of ecological design may be subject to ecological fallacy. In addition, when assessing these associations, we did not consider other parameters such as unemployment rate, life expectancy, health expenditure, and urbanization rate, which can bring in confounding. Finally, we were not able to incorporate UIs of GBD data into the ARIMA models when forecasting, and only the time series of estimated means were used in the models. The predictions are based solely on past trends in suicide mortality and do not include other factors that might affect their future changes.

## Conclusions

The present study found that South Korea and Sub-Saharan Africa had the highest suicide mortality rates for the elderly in 2017. There were significant rate differences between the elderly and the general population, and the largest was in China and Sub-Saharan Africa countries. The deaths number from suicide increased but the mortality rate decreased between 1990 and 2017, yet most countries will not be able to meet the SDGs reduction indicator by 2030 on this trend. Our results showed variability in elderly suicide mortality by sex, age, region, country, and SDI, which could be used to indicate suicide prevention and intervention policies for countries involved. Future study is needed to explore reasons for the variability among different populations, and comprehensive strategies should be adopted to close the gap to the 2030 SDGs.

## Data Availability Statement

Publicly available datasets were analyzed in this study. This data can be found here: http://ghdx.healthdata.org/gbd-2017.

## Author Contributions

SX and JH developed the study idea and designed it. JH, DQ, LL, and YL did the data collection and analyses. JH and FO drafted the manuscript. All authors interpreted the findings, prepared the manuscript, and gave their final approval of the version to be published.

## Funding

This work was supported by the National Key Research and Development Program of China (grant number: 2016YFC0900802). The funders had no role in study design, data collection and analysis, decision to publish, or preparation of the manuscript.

## Conflict of Interest

The authors declare that the research was conducted in the absence of any commercial or financial relationships that could be construed as a potential conflict of interest.

## Publisher's Note

All claims expressed in this article are solely those of the authors and do not necessarily represent those of their affiliated organizations, or those of the publisher, the editors and the reviewers. Any product that may be evaluated in this article, or claim that may be made by its manufacturer, is not guaranteed or endorsed by the publisher.

## Supplementary Material

Supplementary S1 Partial statistical methods used in the study.

Supplementary S2 The socioeconomic indicators used in Supplementary Figure 6.

Supplementary Table 1 Death numbers, mortality rates, and mortality fractions from suicide for people aged 70 and older, and their percentage changes between 1990 and 2017, in 195 countries and territories.

Supplementary Table 2 Mortality rates from suicide for the elderly (70+ years), age-standardized mortality rates from suicide for all ages, and their percentage differences, in 2017, for 21 GBD regions.

Supplementary Table 3 Mortality rates from suicide for the elderly (70+ years), age-standardized mortality rates from suicide for all ages, and their percentage differences, in 2017, for 195 countries and territories.

Supplementary Table 4 Percentage changes in mortality rates from suicide for the elderly (70+ years) between 1990 and 2017, percentage changes in age-standardized mortality rates from suicide for all ages between 1990 and 2017, and their differences, in 21 GBD regions.

Supplementary Table 5 Percentage changes in mortality rates from suicide for the elderly (70+ years) between 1990 and 2017, percentage changes in age-standardized mortality rates from suicide for all ages between 1990 and 2017, and their differences, in 195 countries and territories.

Supplementary Table 6 The orders and coefficients of ARIMA models for globe, sexes, 21 GBD regions, and 195 countries and territories.

Supplementary Figure 1 Differences between percentage changes in mortality rates from suicide for the elderly and percentage changes in age-standardized mortality rates from suicide for all ages between 1990 and 2017. Difference = *P*_elderly_−*P*_std_, where *P*_elderly_ donates percentage change in mortality rate from suicide for the elderly between 1990 and 2017, and R_std_ donates percentage change in age-standardized mortality rate from suicide for all ages between 1990 and 2017. Map was colored only for countries with decline in mortality rate from suicide for both the elderly and all ages, i.e., percentage change is less than zero.

Supplementary Figure 2 Relationship between SDI and elderly suicide mortality rates for each year. SDI = Socio-demographic Index. Models are weighted by the elderly population (point size). Y axes are shown after the square root transformation. Each point corresponds to the mortality rate for one of 195 countries and territories. The line represents the mortality rates expected on the basis of SDI alone, with linear model and restricted cubic spline (RCS) smoother which includes 5 knots at the 5th, 27.5th, 50th, 72.5th, and 95th percentiles.

Supplementary Figure 3 Projected percentage changes in mortality rates from suicide for the elderly between 2015 and 2030. Percentage change = 100 × (*R*_2030_ − *R*_2015_)/*R*_2015_, where *R*_2015_ donates mortality rate from suicide for the elderly in 2015 derived from Global Burden of Disease 2017, and *R*_2030_ donates mortality rate from suicide for the elderly in 2030 which was predicted by Autoregressive Integrated Moving Average (ARIMA) model. Blue regions refer to ten countries and territories that are expected to meet the UN Sustainable Development Goals (SDGs) indicator of a one-third reduction, including Turkey, Singapore, China, Croatia, Iraq, Bulgaria, Latvia, Switzerland, Estonia, and Sweden.

Supplementary Figure 4 Projected national mortality rates from suicide for the elderly up to 2030. Black points and solid lines denote suicide mortality rates derived from Global Burden of Disease study 2017. Blue points and solid lines denote projected suicide mortality rates from 2018 to 2030 based on the mean rates from 1990 to 2017 using Autoregressive Integrated Moving Average (ARIMA) model. Blue dashed lines denote 95% confidence intervals for the projected suicide mortality rates, with the lower bounds being forced to 0 for those <0.

Supplementary Figure 5 Relationship between SDI and elderly suicide mortality rates from 1990 to 2017 for 195 countries and territories fitted by a local polynomial regression. SDI = Socio-demographic Index. Model is weighted by the elderly population. Y axis is shown after the square root transformation. Each hollow point corresponds to the mortality rate for a country or territory in a specific year. For a given location, the gradient colors from light to dark indicate the years from 1990 to 2017. The line represents the mortality rates expected on the basis of SDI, with a local polynomial regression.

Supplementary Figure 6 Relationship of the suicide mortality rates for the elderly with fertility rate **(A)**, GDP per capital **(B)**, and secondary school enrollment **(C)** in 2017. Models are weighted by the elderly population (point size). Y axes are shown after the square root transformation. The black curves represent the mortality rates expected on the basis of each socioeconomic indicator alone, with linear model and restricted cubic spline smoother which includes 5 knots at the 5th, 27.5th, 50th, 72.5th, and 95th percentiles. Each point of a specific color represents one country or territory.

## Abbreviations

95% UI: 95% uncertainty interval
ARIMA: Autoregressive Integrated Moving Average model
GBD: the Global Burden of Diseases Injuries and Risk Factors study
ICD: the International Classification of Diseases
RMAE: relatively mean absolute error
SDGs: UN Sustainable Development Goals
SDI: Socio-demographic index
YLLs: years of life lost.
