# Supplementary material for: Time Trends and Predictions of Suicide Mortality for People Aged 70 Years and Over From 1990 to 2030 Based on the Global Burden of Disease Study 2017
Source: Front Psychiatry. 2021 Sep 27;12:721343. doi: 10.3389/fpsyt.2021.721343 (PMC8502866; doi:10.3389/fpsyt.2021.721343)
Supplement: Supplementary S1 — Partial statistical methods used in the study. [file Data_Sheet_1.zip › Supplementary S1.docx]

**Supplementary S1. Partial statistical methods used in the study**

# 1. Linear regression model with restricted cubic spline function

Linear regression model is a simple algorithm and can approximate many common situations, however, relationships among variables are seldom strictly linear in medical study practice, such as associations of suicide mortality rate with macroeconomic and social factors. This concern can be overcome by adding cubic splines to the linear regression model with highly curved functions. Cubic splines can be made to be smooth by forcing three levels of continuity (the function, its slope or first derivative, and its acceleration or second derivative, i.e., slope of the slope) at the knots. The most common choices are 3, 4, or 5 knots. If a cubic spline function has *k* knots, the function will require estimating *k* + 3 regression coefficients besides the intercept. However, cubic spline functions were found to perform poorly in the tails, that is before the first knot and after the last knot. So the restricted cubic splines (also called natural splines) constrain function to be linear in tails. They have the additional advantage that only *k* − 1 parameters must be estimated (besides the intercept) as opposed to *k* + 3 parameters with the unrestricted cubic spline.

The restricted cubic spline function with k knots *t*_1_, . . . , *t_k_* is given by (1)

*f*(*X*) = *β*_0_ + *β*_1_*X*_1_ + *β*_2_*X*_2_ + *. . .* + *β_k_*_-1_*X_k_*_-1_,

where *X*_1_ = *X* and for *j* = 1, . . . , *k* − 2,

Let

(*u*)_+_ = *u* if *u* > 0,

(*u*)_+_ = 0 if *u* ≤ 0,

${X_{j+1}=(X-t_{j})}_{+}^{3}-\left( X-t_{k-1} \right)_{+}^{3}\frac{(t_{k}-t_{j})}{(t_{k}-t_{k-1})}$*+*$\left( X-t_{k} \right)_{+}^{3}\frac{(t_{k-1}-t_{j})}{(t_{k}-t_{k-1})}$.

Then (*k* – 2) new variables are created, and *X_j_* is linear in *X* for *X* ≥ *t_k_*.

For numerical behavior and to put all basis functions for *X* on the same scale, functions of rms package in R language by default divide the terms above by *τ* = (*t_k_* − *t_1_*)^2^. For a more intuitive understanding, the interactive demonstrations of cubic splines and restricted cubic splines can be found at [pclambert.net/interactivegraphs](https://pclambert.net/interactivegraphs/). The coefficients and corresponding statistical results for Figure 6A are presented in the following table.

| term | estimate | std.error | statistic | p.value |
| --- | --- | --- | --- | --- |
| (Intercept) | 64.245 | 4.599 | 13.968 | <0.0001 |
| rcs(SDI, 5)SDI | -91.345 | 13.459 | -6.787 | <0.0001 |
| rcs(SDI, 5)SDI' | 298.021 | 43.611 | 6.834 | <0.0001 |
| rcs(SDI, 5)SDI'' | -1099.04 | 182.795 | -6.012 | <0.0001 |
| rcs(SDI, 5)SDI''' | 1109.045 | 305.927 | 3.625 | 0.0003 |

# 2. Autoregressive Integrated Moving Average model (ARIMA)

The ARIMA model is a mature Box-Jenkins method used to carry out time series analysis. Optimal parameters of ARIMA could be determined by Akaike's information Criterion(AIC) or Schwarz's Bayesian Criterion(BIC). Box-Ljung Test is used to examine autocorrelation and Relatively Mean Absolute Error (RMAE) is a common metric to test the prediction ability of the model.

Modeling of ARIMA includes three contents: autoregression, moving average, difference and accumulation. The general model is denoted as ARIMA (*p*, *d*, *q*), where *p* and *q* are orders of the ordinary autoregression and moving average, and *d* is order of the difference. ARIMA model is given by (2)

*φ*(*B*)▽*^d^x_t_* = *δ* + *θ*(*B*)*w_t_*,

where *x_t_* is the sequence value at time *t* and *w_t_* is the usual Gaussian white noise process. ▽, *B* and *δ* are on behalf of difference operator, backward shift operator, and constant term respectively. The autoregressive and moving average components are represented by polynomials *φ*(*B*) of order *p* and *θ*(*B*) of order *q* respectively, and the difference component is represented by ▽*^d^* of order *d*.

The specific expressions of each polynomial are

*φ(B)* *=* 1 − *φ*_1_*B* − *φ*_2_*B*^2^ − ··· − *φ_p_B^p^*,

*θ(B)* *= 1* + *θ*_1_*B* + *θ*_2_*B*^2^ + ··· + *θ_q_B^q^*,

▽*^d^* = (1 − *B*)*^d^*.

Smoothing, building, diagnosing and forecasting are four main steps in ARIMA modeling. ARIMA model requires stationary sequences, so in the first step, the stationary characteristic of a sequence should be surveyed. Smoothing is indispensable if the sequence is suspected of instable. In the second step, parameters of ARIMA are identified by observing autocorrelation function (ACF) and partial autocorrelation function (PACF), and the best ARIMA model is constructed accordingly. Then Ljung-Box test for modeling residuals is applied to diagnose if there is autocorrelation in the model sequence. To verify the model’s predictive ability, forecasts are made for the last years and RMAE is calculated. Finally, the model is applied to make predictions for unknow data in the future.

# 3. Local polynomial regression

Local polynomial regression is to fit a polynomial surface determined by one or more numerical predictors, using local fitting. That is, for the fit at point x, the fit is made using points in a neighborhood of x, weighted by their distance from x (with differences in ‘parametric’ variables being ignored when computing the distance). The size of the neighborhood is controlled by α (set by span in loess function in R). For α < 1, the neighborhood includes proportion α of the points, and these have tricubic weighting (proportional to (1 - (dist/maxdist)^3)^3). For α > 1, all points are used, with the ‘maximum distance’ assumed to be α^(1/p) times the actual maximum distance for p explanatory variables. For the default, fitting is by (weighted) least squares (3).

1. Harrell Jr FE. *Regression modeling strategies: with applications to linear models, logistic and ordinal regression, and survival analysis*: springer (2015).

2. Shumway RH, Stoffer DS. *Time Series Analysis and Its Applications: With R Examples, Third Edition*: Springer (2010).

3. R Core Team. R: A language and environment for statistical computing: R Foundation for Statistical Computing (2021). Available from: https://www.R-project.org/.
