# Supplementary material for: Time Trends and Predictions of Suicide Mortality for People Aged 70 Years and Over From 1990 to 2030 Based on the Global Burden of Disease Study 2017
Source: Front Psychiatry. 2021 Sep 27;12:721343. doi: 10.3389/fpsyt.2021.721343 (PMC8502866; doi:10.3389/fpsyt.2021.721343)
Supplement: Supplementary S1 — Partial statistical methods used in the study. [file Data_Sheet_1.zip › Supplementary S2.docx]

**Supplementary S2. The socioeconomic indicators used in Figure S6**

# 1. Fertility rate, total (births per woman)

Total fertility rate represents the number of children that would be born to a woman if she were to live to the end of her childbearing years and bear children in accordance with age-specific fertility rates of the specified year.

Source: (1) United Nations Population Division. World Population Prospects: 2019 Revision. (2) Census reports and other statistical publications from national statistical offices, (3) Eurostat: Demographic Statistics, (4) United Nations Statistical Division. Population and Vital Statistics Reprot (various years), (5) U.S. Census Bureau: International Database, and (6) Secretariat of the Pacific Community: Statistics and Demography Program.

Number of countries and territories matched with GBD 2017: 188.

# 2. GDP per capita (current US$)

GDP per capita is gross domestic product divided by midyear population. GDP is the sum of gross value added by all resident producers in the economy plus any product taxes and minus any subsidies not included in the value of the products. It is calculated without making deductions for depreciation of fabricated assets or for depletion and degradation of natural resources. Data are in current U.S. dollars.

Source: World Bank national accounts data, and OECD National Accounts data files.

Number of countries and territories matched with GBD 2017: 188.

# 3. School enrollment, secondary (% gross)

Gross enrollment ratio is the ratio of total enrollment, regardless of age, to the population of the age group that officially corresponds to the level of education shown. Secondary education completes the provision of basic education that began at the primary level, and aims at laying the foundations for lifelong learning and human development, by offering more subject- or skill-oriented instruction using more specialized teachers.

Source: UNESCO Institute for Statistics (http://uis.unesco.org/). Data as of September 2020.

Number of countries and territories matched with GBD 2017: 123.
