# Supplementary material for: Time Trends and Predictions of Suicide Mortality for People Aged 70 Years and Over From 1990 to 2030 Based on the Global Burden of Disease Study 2017
Source: Front Psychiatry. 2021 Sep 27;12:721343. doi: 10.3389/fpsyt.2021.721343 (PMC8502866; doi:10.3389/fpsyt.2021.721343)
Supplement: Supplementary S1 — Partial statistical methods used in the study. [file Data_Sheet_1.zip › Supplementary Table 1.docx]

**Supplementary Table 1. Death numbers, mortality rates, and mortality fractions from suicide for people aged 70 and older, and their percentage changes between 1990 and 2017, in 195 countries and territories.**

| **Location** | **Death number (95% UI)** | | | **Mortality rate per 100 000 (95% UI)** | | | **Mortality fraction per 1 000 (95% UI)** | | |
| --- | --- | --- | --- | --- | --- | --- | --- | --- | --- |
|  | **1990** | **2017** | **Percentage change (%)** | **1990** | **2017** | **Percentage change (%)** | **1990** | **2017** | **Percentage change (%)** |
| South Korea | 357 (338, 378) | 4157 (3782, 4552) | 1063.0 (930.0, 1198.8) | 28.1 (26.6, 29.7) | 86.0 (78.3, 94.2) | 205.8 (170.8, 241.5) | 3.8 (3.6, 4.0) | 20.3 (18.6, 21.9) | 435.2 (382.4, 493.2) |
| Zimbabwe | 125 (85, 149) | 221 (152, 273) | 77.3 (36.4, 124.9) | 67.1 (45.5, 80.4) | 84.4 (57.9, 104.2) | 25.7 (-3.3, 59.5) | 8.9 (6.1, 10.6) | 9.7 (6.8, 11.7) | 9.0 (-13.5, 34.3) |
| Lesotho | 31 (26, 38) | 44 (33, 56) | 40.6 (0.6, 89.4) | 69.0 (56.7, 83.5) | 82.1 (62.0, 105.8) | 18.9 (-14.9, 60.2) | 7.9 (6.6, 9.4) | 7.9 (6.5, 9.5) | 0.9 (-20.7, 26.3) |
| Greenland | 1 (1, 2) | 2 (2, 4) | 136.3 (90.8, 192.1) | 77.8 (60.2, 138.2) | 77.5 (61.0, 131.7) | -0.5 (-19.6, 23.0) | 8.6 (6.7, 15.2) | 11.7 (9.4, 20.2) | 36.5 (11.9, 68.5) |
| Mozambique | 186 (149, 229) | 313 (242, 383) | 68.5 (22.3, 123.7) | 77.6 (62.1, 95.6) | 77.0 (59.6, 94.4) | -0.7 (-27.9, 31.8) | 7.6 (6.2, 9.4) | 8.7 (6.9, 10.6) | 14.6 (-15.3, 48.8) |
| Senegal | 103 (80, 124) | 232 (174, 287) | 125.0 (67.6, 187.5) | 68.2 (52.6, 82.0) | 71.6 (53.7, 88.4) | 5.1 (-21.7, 34.3) | 7.3 (5.7, 8.7) | 8.9 (6.8, 10.8) | 21.3 (-6.5, 52.5) |
| Cote d'Ivoire | 88 (68, 111) | 258 (206, 317) | 193.1 (113.7, 290.3) | 66.2 (51.4, 83.7) | 70.5 (56.4, 86.8) | 6.5 (-22.3, 41.8) | 7.3 (5.7, 9.1) | 8.6 (7.0, 10.4) | 18.9 (-9.7, 54.6) |
| Liberia | 31 (24, 41) | 54 (44, 67) | 75.4 (35.1, 127.6) | 52.6 (41.3, 69.0) | 70.1 (57.0, 85.7) | 33.4 (2.7, 73.1) | 5.9 (4.7, 7.6) | 8.2 (7.0, 9.7) | 40.1 (10.2, 77.6) |
| Uganda | 230 (181, 318) | 385 (323, 457) | 67.5 (17.6, 123.3) | 79.0 (62.1, 109.4) | 69.0 (57.9, 82.0) | -12.6 (-38.7, 16.5) | 8.5 (6.9, 11.8) | 9.4 (8.1, 10.9) | 9.8 (-22.6, 42.9) |
| Cameroon | 105 (87, 132) | 310 (251, 380) | 194.4 (123.2, 280.8) | 58.8 (48.6, 73.3) | 67.2 (54.4, 82.4) | 14.3 (-13.4, 47.8) | 6.2 (5.2, 7.6) | 8.3 (6.8, 9.6) | 33.7 (3.1, 68.1) |
| Zambia | 97 (77, 122) | 171 (141, 203) | 75.8 (33.7, 127.2) | 84.2 (67.0, 105.5) | 66.1 (54.5, 78.6) | -21.5 (-40.3, 1.5) | 7.8 (6.2, 9.6) | 8.1 (6.8, 9.6) | 4.7 (-19.8, 35.9) |
| Burkina Faso | 130 (100, 159) | 233 (184, 278) | 79.1 (39.7, 136.6) | 67.2 (51.7, 82.0) | 63.2 (49.8, 75.5) | -6.0 (-26.7, 24.2) | 7.0 (5.5, 8.5) | 7.5 (6.0, 8.8) | 6.8 (-16.1, 41.2) |
| Burundi | 70 (56, 87) | 84 (65, 105) | 20.7 (-6.9, 55.3) | 83.7 (67.6, 104.8) | 62.8 (48.8, 78.7) | -24.9 (-42.1, -3.4) | 6.8 (5.5, 8.5) | 6.9 (5.6, 8.6) | 2.0 (-18.4, 28.9) |
| Sri Lanka | 589 (434, 675) | 769 (637, 933) | 30.7 (3.2, 84.9) | 120.7 (88.9, 138.4) | 62.5 (51.8, 75.8) | -48.2 (-59.1, -26.7) | 15.5 (11.4, 17.7) | 11.4 (9.8, 13.5) | -26.9 (-40.4, 2.4) |
| South Sudan | 65 (45, 92) | 82 (58, 112) | 26.1 (-6.9, 72.1) | 65.8 (45.8, 93.0) | 61.1 (43.3, 83.6) | -7.3 (-31.5, 26.6) | 6.0 (4.5, 8.2) | 6.8 (5.2, 8.9) | 12.1 (-12.2, 43.1) |
| Rwanda | 124 (99, 155) | 131 (104, 166) | 5.6 (-19.9, 38.7) | 107.0 (85.2, 133.6) | 60.3 (47.9, 76.4) | -43.6 (-57.2, -25.9) | 8.2 (6.5, 10.2) | 8.2 (6.7, 10.2) | 0.8 (-22.7, 27.4) |
| Malawi | 126 (93, 156) | 222 (183, 264) | 76.5 (31.6, 138.6) | 66.4 (49.2, 82.4) | 60.2 (49.5, 71.7) | -9.2 (-32.3, 22.7) | 6.4 (4.8, 7.9) | 7.7 (6.3, 9.1) | 19.2 (-10.3, 63.3) |
| Congo | 27 (21, 33) | 54 (40, 69) | 96.3 (40.4, 157.2) | 62.8 (48.7, 75.4) | 59.1 (43.7, 75.5) | -5.9 (-32.7, 23.3) | 5.5 (4.2, 6.5) | 6.4 (4.8, 8.2) | 18.0 (-13.2, 50.3) |
| Kenya | 232 (181, 350) | 461 (409, 534) | 98.6 (36.0, 144.8) | 64.7 (50.4, 97.4) | 56.5 (50.1, 65.4) | -12.7 (-40.2, 7.6) | 7.7 (6.0, 11.6) | 7.5 (6.7, 8.7) | -2.3 (-33.0, 20.0) |
| Chad | 62 (43, 90) | 131 (97, 185) | 112.0 (63.5, 172.8) | 40.7 (28.4, 59.5) | 55.8 (41.1, 78.5) | 37.0 (5.7, 76.3) | 4.4 (3.1, 6.4) | 6.2 (4.7, 8.6) | 42.7 (13.1, 78.4) |
| Guinea | 63 (43, 99) | 136 (97, 191) | 116.4 (56.1, 185.8) | 37.9 (26.0, 59.9) | 55.0 (39.2, 77.2) | 45.0 (4.6, 91.5) | 3.9 (2.7, 6.2) | 5.5 (4.0, 7.6) | 39.6 (1.9, 81.7) |
| Suriname | 8 (7, 8) | 16 (14, 18) | 111.3 (81.1, 147.8) | 62.7 (56.5, 68.9) | 55.0 (48.1, 62.5) | -12.3 (-24.9, 2.8) | 8.6 (7.8, 9.3) | 8.5 (7.6, 9.5) | -0.7 (-13.1, 13.7) |
| Eritrea | 19 (16, 22) | 38 (30, 46) | 101.9 (51.6, 166.2) | 72.6 (60.0, 86.9) | 54.9 (43.8, 66.3) | -24.3 (-43.2, -0.2) | 5.4 (4.5, 6.4) | 6.2 (5.1, 7.5) | 14.3 (-12.8, 48.2) |
| Angola | 87 (59, 114) | 193 (135, 239) | 122.8 (58.6, 202.9) | 68.1 (46.4, 89.1) | 54.8 (38.4, 67.6) | -19.5 (-42.7, 9.5) | 6.2 (4.2, 7.9) | 7.0 (5.0, 8.7) | 14.2 (-17.0, 50.3) |
| Central African Republic | 21 (16, 27) | 34 (26, 42) | 62.6 (20.7, 108.9) | 59.6 (44.4, 74.9) | 54.7 (41.8, 67.6) | -8.1 (-31.8, 18.0) | 5.0 (3.7, 6.3) | 5.1 (3.9, 6.2) | 2.9 (-23.0, 33.1) |
| Guinea-Bissau | 9 (7, 12) | 14 (11, 17) | 53.3 (11.4, 101.1) | 54.9 (40.6, 72.4) | 54.7 (42.0, 67.6) | -0.4 (-27.7, 30.5) | 4.5 (3.4, 5.9) | 5.7 (4.4, 7.1) | 26.6 (-6.2, 63.6) |
| Benin | 52 (41, 67) | 110 (90, 135) | 113.3 (59.4, 176.8) | 49.9 (39.6, 65.2) | 54.4 (44.2, 66.7) | 9.0 (-18.6, 41.4) | 5.1 (4.1, 6.7) | 6.7 (5.6, 8.0) | 31.9 (2.1, 65.5) |
| Ethiopia | 577 (459, 689) | 911 (768, 1106) | 57.8 (17.2, 110.1) | 81.9 (65.2, 97.7) | 53.3 (45.0, 64.7) | -35.0 (-51.7, -13.4) | 6.8 (5.5, 8.1) | 7.7 (6.6, 9.2) | 12.9 (-14.7, 47.2) |
| Gabon | 14 (11, 17) | 24 (16, 30) | 68.4 (17.5, 125.0) | 52.4 (41.0, 63.3) | 53.1 (37.1, 67.8) | 1.3 (-29.3, 35.3) | 5.4 (4.2, 6.5) | 6.9 (4.8, 8.8) | 27.3 (-9.8, 70.5) |
| Serbia | 336 (262, 385) | 507 (445, 563) | 51.0 (29.0, 81.8) | 65.0 (50.7, 74.5) | 51.7 (45.3, 57.3) | -20.5 (-32.1, -4.3) | 6.9 (5.4, 8.0) | 5.7 (5.0, 6.3) | -17.7 (-29.6, -1.2) |
| Togo | 24 (20, 32) | 67 (55, 81) | 176.3 (110.4, 257.2) | 48.1 (38.5, 62.3) | 51.7 (42.1, 62.3) | 7.3 (-18.3, 38.7) | 5.3 (4.3, 6.8) | 7.0 (5.9, 8.2) | 31.0 (0.5, 65.5) |
| Madagascar | 133 (106, 170) | 168 (133, 210) | 26.0 (-2.4, 57.4) | 62.6 (49.5, 79.9) | 49.4 (39.1, 61.9) | -21.0 (-38.8, -1.3) | 6.3 (5.0, 7.9) | 5.7 (4.7, 7.0) | -9.6 (-26.8, 9.9) |
| Guyana | 9 (8, 10) | 13 (11, 15) | 38.0 (17.1, 62.4) | 52.4 (47.3, 57.5) | 49.1 (42.6, 56.8) | -6.5 (-20.6, 10.1) | 6.1 (5.5, 6.7) | 6.7 (6.0, 7.6) | 10.0 (-5.5, 27.6) |
| Cape Verde | 5 (4, 7) | 11 (9, 16) | 145.0 (90.7, 207.9) | 31.9 (25.7, 49.2) | 48.7 (40.9, 71.7) | 52.7 (18.9, 91.9) | 5.3 (4.3, 8.2) | 7.2 (6.2, 10.6) | 37.0 (7.4, 69.7) |
| Sierra Leone | 37 (27, 53) | 74 (54, 103) | 102.9 (40.9, 175.0) | 35.4 (26.1, 51.4) | 47.9 (34.5, 66.5) | 35.5 (-5.9, 83.7) | 3.5 (2.7, 4.9) | 5.3 (3.9, 7.2) | 50.8 (6.7, 101.4) |
| Democratic Republic of the Congo | 304 (228, 380) | 571 (440, 691) | 87.8 (43.6, 141.9) | 53.9 (40.4, 67.5) | 47.8 (36.8, 57.9) | -11.3 (-32.2, 14.2) | 5.7 (4.3, 7.1) | 5.4 (4.2, 6.5) | -5.8 (-26.0, 19.6) |
| Tanzania | 264 (221, 318) | 515 (416, 634) | 95.0 (50.9, 150.1) | 53.7 (44.9, 64.6) | 47.5 (38.4, 58.4) | -11.5 (-31.5, 13.5) | 6.2 (5.2, 7.4) | 6.6 (5.4, 8.1) | 5.8 (-17.8, 31.5) |
| The Gambia | 6 (4, 8) | 21 (16, 30) | 259.6 (167.9, 386.5) | 38.6 (28.3, 55.8) | 47.1 (34.8, 66.0) | 22.1 (-9.1, 65.2) | 4.4 (3.4, 6.4) | 5.4 (4.1, 7.4) | 21.2 (-8.2, 58.3) |
| Cuba | 379 (360, 398) | 535 (476, 608) | 41.3 (23.9, 60.8) | 60.7 (57.6, 63.7) | 46.8 (41.6, 53.1) | -23.0 (-32.5, -12.4) | 9.2 (8.7, 9.7) | 8.0 (7.3, 8.8) | -12.9 (-21.7, -3.4) |
| Ghana | 87 (70, 110) | 292 (219, 356) | 237.0 (135.5, 356.3) | 33.9 (27.2, 43.2) | 46.5 (34.9, 56.8) | 37.3 (-4.1, 85.9) | 3.9 (3.2, 4.9) | 5.8 (4.3, 7.0) | 47.8 (3.1, 96.3) |
| Nigeria | 1047 (772, 1371) | 1577 (1176, 2140) | 50.7 (8.4, 112.4) | 48.2 (35.6, 63.2) | 45.7 (34.1, 62.0) | -5.3 (-31.9, 33.4) | 5.5 (4.4, 6.7) | 6.4 (5.4, 7.8) | 16.6 (-5.2, 41.5) |
| Equatorial Guinea | 5 (3, 6) | 8 (6, 11) | 81.4 (16.5, 161.0) | 62.7 (46.0, 81.4) | 45.0 (31.3, 60.8) | -28.2 (-53.9, 3.3) | 5.0 (3.8, 6.4) | 6.5 (4.9, 7.9) | 29.5 (-11.7, 72.8) |
| Somalia | 41 (26, 66) | 94 (62, 152) | 128.0 (66.8, 213.0) | 53.3 (33.4, 85.8) | 44.7 (29.6, 72.2) | -16.1 (-38.6, 15.2) | 4.4 (2.8, 6.8) | 4.8 (3.4, 7.3) | 9.0 (-15.3, 41.6) |
| Djibouti | 2 (1, 3) | 8 (5, 13) | 335.4 (203.4, 518.0) | 42.1 (26.3, 73.0) | 42.6 (26.8, 68.9) | 1.2 (-29.5, 43.7) | 5.0 (3.3, 8.4) | 6.1 (4.2, 9.2) | 20.5 (-11.4, 58.6) |
| Lithuania | 104 (98, 110) | 166 (152, 181) | 59.6 (44.2, 76.4) | 40.8 (38.4, 43.3) | 41.5 (38.0, 45.4) | 1.7 (-8.1, 12.4) | 4.9 (4.7, 5.2) | 6.1 (5.6, 6.6) | 22.4 (11.3, 34.6) |
| Taiwan | 321 (302, 338) | 839 (776, 904) | 161.6 (139.5, 185.5) | 44.0 (41.4, 46.3) | 40.9 (37.8, 44.1) | -7.1 (-14.9, 1.4) | 7.0 (6.6, 7.4) | 7.4 (6.9, 7.8) | 4.8 (-3.4, 13.4) |
| Slovenia | 85 (79, 90) | 110 (99, 123) | 30.2 (15.0, 47.5) | 64.1 (60.0, 68.3) | 40.8 (36.8, 45.4) | -36.3 (-43.7, -27.8) | 8.0 (7.5, 8.6) | 7.5 (6.8, 8.2) | -7.1 (-16.8, 4.5) |
| Comoros | 4 (3, 6) | 8 (6, 14) | 129.4 (71.0, 199.0) | 45.5 (31.4, 74.2) | 40.8 (28.0, 65.6) | -10.3 (-33.1, 17.0) | 4.8 (3.3, 7.6) | 5.4 (3.8, 8.6) | 13.4 (-11.7, 43.7) |
| Belarus | 199 (189, 210) | 367 (332, 406) | 84.3 (64.2, 105.3) | 28.4 (26.9, 30.0) | 40.6 (36.7, 44.8) | 42.9 (27.3, 59.2) | 3.5 (3.3, 3.7) | 5.0 (4.5, 5.5) | 43.9 (29.5, 59.7) |
| Hungary | 823 (784, 859) | 494 (455, 535) | -40.0 (-45.4, -34.3) | 96.1 (91.5, 100.2) | 39.7 (36.6, 43.0) | -58.7 (-62.4, -54.8) | 10.4 (9.9, 10.8) | 6.1 (5.7, 6.5) | -41.2 (-45.9, -36.3) |
| Uruguay | 74 (70, 80) | 139 (122, 158) | 86.8 (58.8, 115.4) | 30.3 (28.3, 32.7) | 38.9 (34.0, 44.1) | 28.1 (8.9, 47.8) | 4.2 (4.0, 4.6) | 6.0 (5.4, 6.7) | 41.8 (23.8, 60.8) |
| Russian Federation | 4525 (4434, 4672) | 4964 (4792, 5089) | 9.7 (4.9, 13.6) | 46.7 (45.8, 48.2) | 38.8 (37.4, 39.7) | -17.1 (-20.7, -14.1) | 5.7 (5.6, 5.9) | 5.0 (4.9, 5.2) | -11.8 (-15.6, -8.6) |
| China | 27926 (23312, 30133) | 37918 (34716, 40235) | 35.8 (22.2, 61.3) | 72.2 (60.3, 77.9) | 38.5 (35.2, 40.9) | -46.7 (-52.0, -36.7) | 9.0 (7.5, 9.7) | 6.1 (5.7, 6.5) | -31.7 (-38.1, -19.2) |
| Kazakhstan | 232 (219, 245) | 275 (252, 300) | 18.9 (7.5, 31.1) | 38.4 (36.3, 40.5) | 37.1 (34.0, 40.5) | -3.3 (-12.5, 6.7) | 4.8 (4.6, 5.1) | 4.7 (4.3, 5.1) | -2.3 (-11.1, 7.6) |
| Croatia | 220 (208, 233) | 212 (194, 232) | -4.0 (-13.7, 6.5) | 67.6 (63.7, 71.4) | 37.1 (34.0, 40.6) | -45.1 (-50.7, -39.1) | 7.4 (7.0, 7.8) | 5.6 (5.2, 6.0) | -24.5 (-31.5, -17.5) |
| Austria | 427 (406, 447) | 432 (397, 473) | 1.1 (-8.3, 11.3) | 56.1 (53.5, 58.8) | 36.5 (33.6, 40.0) | -34.9 (-40.9, -28.3) | 7.4 (7.1, 7.8) | 6.8 (6.3, 7.4) | -7.8 (-15.6, 0.5) |
| Niger | 36 (24, 63) | 102 (70, 179) | 182.3 (117.9, 262.2) | 33.8 (22.4, 58.8) | 35.9 (24.5, 62.8) | 6.4 (-17.9, 36.5) | 3.6 (2.4, 6.3) | 4.8 (3.3, 8.4) | 32.7 (4.6, 67.7) |
| Mauritania | 17 (12, 25) | 33 (23, 51) | 92.9 (39.2, 159.3) | 32.4 (23.4, 47.9) | 35.8 (24.3, 55.0) | 10.3 (-20.4, 48.3) | 3.3 (2.4, 4.9) | 4.9 (3.4, 7.3) | 46.4 (10.1, 88.3) |
| Armenia | 12 (11, 12) | 76 (70, 83) | 560.0 (486.5, 644.6) | 10.1 (9.2, 10.9) | 34.4 (31.7, 37.3) | 241.8 (203.7, 285.6) | 1.2 (1.1, 1.3) | 4.3 (3.9, 4.6) | 263.2 (224.9, 303.9) |
| Ukraine | 1445 (1383, 1507) | 1585 (1469, 1720) | 9.7 (1.0, 19.9) | 36.0 (34.5, 37.5) | 33.8 (31.3, 36.6) | -6.1 (-13.6, 2.6) | 4.2 (4.1, 4.4) | 4.0 (3.7, 4.3) | -6.4 (-12.9, 1.9) |
| France | 3622 (3463, 3807) | 2958 (2691, 3237) | -18.3 (-25.7, -9.9) | 69.8 (66.7, 73.3) | 33.4 (30.4, 36.5) | -52.1 (-56.5, -47.2) | 10.2 (9.7, 10.7) | 6.7 (6.1, 7.2) | -34.4 (-39.9, -28.1) |
| Mali | 50 (33, 84) | 116 (73, 224) | 132.3 (73.2, 201.5) | 31.2 (20.6, 52.6) | 32.9 (20.7, 63.8) | 5.7 (-21.2, 37.3) | 3.1 (2.0, 5.2) | 4.4 (2.8, 8.1) | 42.2 (8.9, 83.1) |
| Japan | 5253 (5181, 5322) | 8197 (7802, 8521) | 56.0 (48.6, 62.9) | 53.3 (52.6, 54.0) | 32.4 (30.8, 33.7) | -39.2 (-42.1, -36.6) | 9.9 (9.7, 10.0) | 7.2 (6.8, 7.4) | -27.4 (-30.7, -24.6) |
| Portugal | 380 (360, 402) | 521 (474, 572) | 37.1 (22.6, 52.9) | 44.3 (42.0, 46.9) | 32.0 (29.1, 35.1) | -27.9 (-35.5, -19.5) | 5.8 (5.5, 6.1) | 5.8 (5.3, 6.3) | -0.1 (-9.8, 10.8) |
| Swaziland | 4 (3, 5) | 8 (6, 10) | 83.2 (37.3, 140.1) | 33.9 (27.7, 41.3) | 31.7 (23.7, 40.0) | -6.4 (-29.8, 22.7) | 3.6 (3.0, 4.4) | 3.9 (3.0, 4.7) | 7.1 (-16.4, 32.1) |
| Malaysia | 145 (108, 171) | 361 (298, 413) | 148.4 (103.0, 205.8) | 36.1 (26.9, 42.5) | 31.1 (25.6, 35.6) | -13.8 (-29.6, 6.1) | 4.3 (3.2, 5.1) | 4.6 (3.8, 5.2) | 5.9 (-12.9, 30.2) |
| Bulgaria | 423 (404, 446) | 312 (284, 343) | -26.1 (-33.6, -18.1) | 62.4 (59.6, 65.9) | 31.0 (28.2, 34.1) | -50.3 (-55.3, -44.8) | 7.3 (7.0, 7.7) | 4.3 (3.9, 4.7) | -42.0 (-47.4, -36.0) |
| Montenegro | 9 (8, 10) | 18 (15, 20) | 102.3 (66.5, 142.4) | 27.6 (24.3, 31.3) | 30.9 (26.1, 35.4) | 11.8 (-8.0, 34.0) | 3.7 (3.2, 4.2) | 4.1 (3.5, 4.7) | 12.4 (-6.3, 33.9) |
| Latvia | 109 (103, 116) | 86 (76, 97) | -21.5 (-31.9, -9.7) | 52.5 (49.5, 55.7) | 30.8 (27.3, 35.0) | -41.3 (-49.1, -32.5) | 5.9 (5.5, 6.2) | 4.5 (4.1, 5.0) | -23.0 (-31.9, -13.5) |
| Guam | 1 (1, 1) | 3 (2, 3) | 199.5 (153.3, 254.2) | 30.8 (27.0, 35.8) | 30.7 (26.9, 35.2) | -0.4 (-15.8, 17.7) | 5.1 (4.5, 5.9) | 4.9 (4.4, 5.5) | -4.2 (-18.9, 11.8) |
| Iceland | 7 (6, 8) | 9 (8, 10) | 36.6 (19.4, 56.5) | 38.1 (34.8, 41.5) | 29.2 (26.3, 32.5) | -23.2 (-32.9, -12.1) | 5.7 (5.3, 6.2) | 5.7 (5.1, 6.2) | -1.5 (-13.4, 11.8) |
| Belgium | 446 (424, 467) | 432 (393, 472) | -3.0 (-12.9, 7.3) | 46.5 (44.3, 48.8) | 29.1 (26.4, 31.7) | -37.5 (-43.9, -30.9) | 6.2 (5.9, 6.5) | 5.1 (4.7, 5.5) | -17.4 (-24.6, -9.5) |
| Botswana | 8 (6, 9) | 17 (14, 21) | 116.3 (70.8, 176.3) | 29.7 (24.3, 36.2) | 28.9 (23.5, 36.3) | -2.7 (-23.2, 24.2) | 3.9 (3.2, 4.7) | 3.9 (3.2, 4.7) | -0.8 (-20.3, 22.1) |
| Germany | 3220 (3094, 3352) | 3694 (3319, 4135) | 14.7 (2.2, 29.1) | 39.8 (38.2, 41.4) | 28.8 (25.8, 32.2) | -27.7 (-35.6, -18.7) | 5.1 (4.9, 5.3) | 5.0 (4.6, 5.4) | -2.1 (-10.1, 6.2) |
| India | 4356 (3335, 5031) | 14626 (11921, 15965) | 235.7 (178.8, 285.8) | 24.7 (18.9, 28.6) | 28.5 (23.3, 31.2) | 15.3 (-4.2, 32.6) | 2.6 (2.0, 3.0) | 3.7 (3.0, 4.0) | 41.9 (19.2, 61.5) |
| Northern Mariana Islands | 0 | 0 (0, 1) | 243.8 (167.5, 340.0) | 24.0 (20.0, 29.6) | 28.4 (24.1, 33.6) | 18.5 (-7.7, 51.7) | 3.9 (3.3, 4.6) | 4.5 (3.9, 5.1) | 14.7 (-5.0, 40.1) |
| Luxembourg | 13 (12, 15) | 17 (15, 19) | 23.5 (6.0, 43.1) | 39.3 (36.3, 43.1) | 28.3 (25.0, 32.0) | -27.9 (-38.1, -16.5) | 5.3 (4.9, 5.8) | 5.0 (4.5, 5.5) | -5.7 (-16.3, 7.8) |
| Marshall Islands | 0 | 0 | -1.9 (-21.4, 22.1) | 34.4 (26.6, 43.1) | 28.1 (22.5, 34.2) | -18.5 (-34.7, 1.5) | 3.4 (2.6, 4.2) | 3.1 (2.6, 3.8) | -7.0 (-23.9, 13.5) |
| Switzerland | 338 (321, 355) | 308 (278, 340) | -9.0 (-18.3, 1.5) | 48.6 (46.2, 51.1) | 27.4 (24.7, 30.3) | -43.7 (-49.5, -37.2) | 7.6 (7.2, 8.0) | 5.9 (5.4, 6.5) | -21.8 (-29.3, -13.6) |
| Namibia | 12 (10, 15) | 19 (15, 24) | 53.9 (22.1, 95.3) | 33.4 (27.2, 40.9) | 26.8 (21.3, 33.6) | -19.7 (-36.3, 1.9) | 3.9 (3.2, 4.7) | 3.7 (2.9, 4.5) | -6.0 (-26.6, 16.1) |
| Estonia | 56 (52, 61) | 47 (41, 54) | -16.0 (-28.8, -1.6) | 47.5 (44.1, 51.1) | 26.5 (22.9, 30.3) | -44.2 (-52.7, -34.6) | 5.3 (5.0, 5.7) | 4.4 (3.9, 5.0) | -17.4 (-28.2, -4.8) |
| North Korea | 205 (166, 246) | 445 (355, 571) | 117.4 (73.7, 174.6) | 30.9 (25.1, 37.1) | 25.4 (20.3, 32.6) | -17.7 (-34.2, 4.0) | 4.2 (3.4, 5.0) | 3.6 (2.9, 4.7) | -13.0 (-29.4, 6.6) |
| Federated States of Micronesia | 1 (1, 1) | 1 (0, 1) | -20.5 (-34.8, -3.3) | 29.1 (23.3, 34.7) | 24.6 (20.7, 28.6) | -15.5 (-30.7, 2.7) | 2.9 (2.3, 3.4) | 2.6 (2.2, 3.0) | -11.3 (-25.1, 5.9) |
| Kiribati | 0 | 1 (0, 1) | 47.0 (17.8, 83.5) | 26.3 (22.5, 30.9) | 24.5 (20.3, 29.1) | -7.1 (-25.6, 15.9) | 2.5 (2.2, 3.0) | 2.5 (2.1, 2.9) | -3.0 (-21.2, 19.5) |
| Singapore | 60 (56, 64) | 82 (74, 91) | 37.0 (21.5, 55.6) | 55.5 (51.7, 59.6) | 24.1 (21.7, 26.8) | -56.7 (-61.6, -50.8) | 9.1 (8.5, 9.7) | 6.7 (6.1, 7.3) | -26.9 (-34.4, -18.2) |
| Samoa | 1 (1, 1) | 1 (1, 2) | 76.6 (42.6, 118.1) | 24.3 (19.2, 29.4) | 23.6 (19.7, 27.5) | -2.8 (-21.5, 20.0) | 3.4 (2.7, 4.0) | 2.9 (2.4, 3.3) | -15.1 (-29.7, 0.6) |
| Vanuatu | 1 (1, 1) | 2 (1, 2) | 135.4 (85.0, 198.2) | 27.2 (20.5, 34.3) | 23.5 (19.0, 29.0) | -13.4 (-32.0, 9.7) | 2.7 (2.1, 3.3) | 2.5 (2.1, 3.0) | -8.6 (-26.5, 11.4) |
| Czech Republic | 448 (426, 471) | 319 (290, 348) | -28.9 (-36.3, -20.4) | 57.0 (54.2, 59.8) | 23.5 (21.4, 25.7) | -58.8 (-63.1, -53.9) | 5.8 (5.5, 6.1) | 4.0 (3.7, 4.4) | -30.4 (-36.6, -23.1) |
| Dominican Republic | 24 (21, 30) | 112 (82, 134) | 363.7 (215.7, 486.0) | 12.7 (11.1, 15.6) | 22.9 (16.7, 27.4) | 80.4 (22.8, 127.9) | 2.4 (2.2, 3.0) | 3.8 (2.8, 4.4) | 56.5 (7.8, 91.4) |
| Denmark | 288 (273, 303) | 172 (155, 189) | -40.3 (-46.5, -32.9) | 51.9 (49.1, 54.6) | 22.5 (20.3, 24.7) | -56.5 (-61.0, -51.1) | 7.0 (6.6, 7.3) | 4.2 (3.8, 4.6) | -39.9 (-45.4, -33.4) |
| Afghanistan | 79 (50, 101) | 102 (68, 122) | 29.5 (-1.1, 76.4) | 24.7 (15.7, 31.6) | 22.2 (14.8, 26.4) | -10.2 (-31.4, 22.3) | 2.2 (1.4, 2.7) | 2.5 (1.6, 2.9) | 12.4 (-14.1, 53.1) |
| Macedonia | 25 (20, 29) | 42 (35, 49) | 66.5 (38.2, 98.7) | 29.5 (23.1, 33.8) | 22.1 (18.2, 25.7) | -25.2 (-37.9, -10.7) | 3.5 (2.8, 4.0) | 3.6 (2.9, 4.1) | 1.7 (-15.3, 19.8) |
| Haiti | 30 (23, 40) | 59 (46, 76) | 97.7 (52.5, 157.1) | 23.1 (17.9, 31.0) | 21.8 (17.1, 28.0) | -5.6 (-27.2, 22.8) | 2.3 (1.8, 3.1) | 2.5 (2.0, 3.2) | 10.2 (-12.8, 39.3) |
| Vietnam | 447 (363, 540) | 901 (754, 1048) | 101.5 (58.2, 150.5) | 22.0 (17.9, 26.6) | 21.7 (18.1, 25.2) | -1.5 (-22.6, 22.5) | 2.8 (2.3, 3.4) | 3.1 (2.6, 3.5) | 9.3 (-11.7, 34.0) |
| Bosnia and Herzegovina | 36 (33, 43) | 74 (64, 94) | 103.7 (72.1, 140.9) | 22.3 (20.0, 26.1) | 21.5 (18.5, 27.4) | -3.7 (-18.6, 13.9) | 2.9 (2.6, 3.4) | 3.0 (2.6, 3.7) | 1.5 (-13.3, 19.6) |
| Virgin Islands, U.S. | 1 (1, 1) | 2 (2, 3) | 191.6 (130.0, 259.4) | 21.0 (18.6, 23.6) | 21.4 (17.2, 25.5) | 1.9 (-19.6, 25.6) | 3.1 (2.7, 3.5) | 3.4 (3.0, 4.0) | 12.5 (-7.4, 35.7) |
| Seychelles | 1 (1, 1) | 1 (1, 1) | -12.5 (-28.4, 37.3) | 36.6 (22.1, 43.4) | 21.2 (18.3, 23.9) | -42.1 (-52.6, -9.1) | 4.6 (2.8, 5.5) | 2.9 (2.5, 3.2) | -37.9 (-48.9, -2.7) |
| Georgia | 39 (36, 42) | 72 (65, 79) | 84.1 (62.6, 105.2) | 12.5 (11.5, 13.5) | 20.6 (18.5, 22.5) | 65.1 (45.8, 84.0) | 1.5 (1.4, 1.6) | 2.2 (2.0, 2.4) | 45.9 (29.2, 62.8) |
| Papua New Guinea | 11 (8, 14) | 24 (19, 30) | 121.0 (76.9, 174.8) | 22.3 (16.8, 29.6) | 20.3 (16.2, 25.6) | -9.1 (-27.2, 13.1) | 2.1 (1.6, 2.7) | 2.0 (1.6, 2.5) | -3.3 (-22.4, 18.8) |
| Argentina | 590 (560, 622) | 663 (586, 751) | 12.3 (-1.2, 29.4) | 32.0 (30.3, 33.7) | 20.3 (17.9, 22.9) | -36.6 (-44.3, -27.0) | 4.5 (4.3, 4.8) | 3.2 (2.9, 3.5) | -30.3 (-37.2, -22.4) |
| Solomon Islands | 1 (1, 2) | 2 (2, 3) | 113.0 (70.0, 169.5) | 24.1 (16.9, 32.9) | 19.6 (15.6, 25.0) | -18.8 (-35.2, 2.7) | 2.5 (1.8, 3.4) | 2.2 (1.8, 2.7) | -14.1 (-30.3, 5.9) |
| Sweden | 351 (334, 367) | 282 (260, 304) | -19.6 (-26.7, -12.5) | 32.4 (30.8, 33.9) | 19.5 (18.0, 21.0) | -39.8 (-45.1, -34.5) | 4.9 (4.7, 5.1) | 3.8 (3.5, 4.0) | -23.5 (-29.5, -17.4) |
| United States | 5012 (4934, 5106) | 6351 (6096, 6575) | 26.7 (21.6, 31.8) | 23.6 (23.2, 24.0) | 19.2 (18.5, 19.9) | -18.5 (-21.9, -15.3) | 3.8 (3.7, 3.9) | 3.4 (3.3, 3.5) | -9.7 (-13.2, -6.5) |
| South Africa | 186 (166, 227) | 391 (363, 460) | 110.3 (76.6, 134.4) | 18.2 (16.3, 22.3) | 18.8 (17.4, 22.1) | 2.9 (-13.6, 14.7) | 3.1 (2.8, 3.7) | 2.9 (2.7, 3.5) | -4.4 (-19.9, 5.9) |
| Netherlands | 332 (315, 348) | 400 (370, 431) | 20.7 (10.0, 31.8) | 26.0 (24.7, 27.2) | 18.7 (17.3, 20.1) | -28.1 (-34.5, -21.4) | 3.7 (3.6, 3.9) | 3.4 (3.2, 3.6) | -8.4 (-15.5, -0.6) |
| Finland | 137 (129, 146) | 150 (135, 167) | 9.4 (-3.3, 23.4) | 30.5 (28.7, 32.5) | 18.6 (16.6, 20.6) | -39.1 (-46.2, -31.3) | 4.3 (4.0, 4.5) | 3.6 (3.2, 3.9) | -16.1 (-25.4, -6.4) |
| Belize | 1 (1, 1) | 2 (2, 2) | 120.7 (80.5, 163.6) | 19.1 (17.0, 22.0) | 18.5 (16.3, 21.0) | -2.8 (-20.5, 16.1) | 2.8 (2.5, 3.2) | 3.3 (2.9, 3.8) | 20.4 (-0.7, 42.1) |
| Slovakia | 86 (75, 100) | 96 (84, 117) | 12.2 (-6.9, 33.7) | 26.3 (23.1, 30.5) | 18.5 (16.1, 22.4) | -29.8 (-41.8, -16.3) | 2.9 (2.5, 3.4) | 2.9 (2.5, 3.5) | -1.2 (-17.5, 17.8) |
| Timor-Leste | 2 (1, 3) | 8 (6, 10) | 279.2 (155.1, 462.3) | 24.7 (18.3, 33.3) | 18.4 (13.8, 23.8) | -25.4 (-49.8, 10.7) | 2.8 (2.1, 3.8) | 2.8 (2.1, 3.5) | -2.9 (-33.3, 39.1) |
| Saint Lucia | 1 (1, 1) | 2 (2, 2) | 70.5 (45.1, 98.7) | 22.9 (20.5, 25.4) | 18.0 (15.9, 20.3) | -21.2 (-33.0, -8.2) | 2.8 (2.5, 3.1) | 2.8 (2.5, 3.2) | 2.6 (-12.6, 19.3) |
| Saint Vincent and the Grenadines | 1 (1, 1) | 1 (1, 2) | 79.9 (51.2, 111.6) | 17.2 (15.3, 19.2) | 17.9 (15.7, 20.2) | 4.4 (-12.3, 22.7) | 2.2 (2.0, 2.5) | 2.7 (2.4, 3.0) | 20.2 (1.7, 41.4) |
| Nepal | 54 (40, 72) | 184 (145, 222) | 237.9 (153.9, 344.4) | 15.0 (10.9, 19.8) | 17.9 (14.2, 21.6) | 19.7 (-10.1, 57.4) | 1.6 (1.2, 2.1) | 2.4 (1.9, 2.8) | 48.6 (15.2, 90.8) |
| Thailand | 357 (306, 419) | 914 (799, 1043) | 156.1 (111.6, 208.6) | 23.3 (20.0, 27.3) | 17.7 (15.5, 20.2) | -24.0 (-37.2, -8.4) | 3.4 (3.0, 4.0) | 4.0 (3.5, 4.4) | 14.8 (-3.6, 34.6) |
| Chile | 236 (218, 256) | 237 (206, 270) | 0.4 (-13.2, 16.0) | 44.0 (40.7, 47.7) | 17.6 (15.4, 20.1) | -59.9 (-65.3, -53.7) | 6.2 (5.7, 6.7) | 3.3 (2.9, 3.6) | -46.6 (-53.3, -39.0) |
| Fiji | 2 (2, 3) | 5 (4, 6) | 109.4 (55.4, 163.2) | 17.6 (14.8, 22.4) | 17.6 (14.7, 20.5) | -0.1 (-25.9, 25.5) | 2.1 (1.8, 2.7) | 2.1 (1.8, 2.3) | -2.7 (-25.1, 18.1) |
| Bhutan | 2 (2, 2) | 5 (4, 7) | 156.4 (81.9, 247.0) | 19.0 (15.0, 23.1) | 17.4 (12.6, 22.9) | -8.8 (-35.3, 23.5) | 2.1 (1.7, 2.5) | 2.8 (2.1, 3.4) | 30.6 (-0.7, 67.6) |
| Laos | 24 (17, 33) | 30 (22, 38) | 25.2 (-8.8, 87.3) | 26.7 (19.3, 36.8) | 17.1 (12.6, 22.2) | -36.0 (-53.4, -4.3) | 2.5 (1.8, 3.5) | 2.2 (1.7, 2.8) | -12.8 (-34.4, 27.4) |
| Brunei | 1 (0, 1) | 2 (1, 2) | 263.5 (137.9, 370.5) | 12.3 (9.7, 14.5) | 16.9 (9.7, 20.6) | 37.6 (-9.9, 78.2) | 1.3 (1.0, 1.5) | 2.7 (1.5, 3.2) | 102.7 (34.3, 154.6) |
| Poland | 385 (366, 406) | 687 (627, 754) | 78.1 (59.9, 99.0) | 15.9 (15.1, 16.7) | 16.8 (15.3, 18.4) | 5.7 (-5.1, 18.1) | 1.9 (1.8, 2.0) | 2.7 (2.5, 3.0) | 46.4 (32.7, 62.7) |
| Spain | 897 (858, 937) | 1063 (971, 1162) | 18.4 (7.5, 30.2) | 25.8 (24.7, 27.0) | 16.6 (15.1, 18.1) | -35.8 (-41.8, -29.5) | 4.0 (3.9, 4.2) | 3.2 (2.9, 3.4) | -21.5 (-28.1, -14.3) |
| Kyrgyzstan | 40 (37, 44) | 28 (26, 31) | -29.3 (-37.7, -19.5) | 27.3 (24.9, 29.8) | 16.3 (14.7, 17.9) | -40.2 (-47.3, -31.9) | 3.3 (3.0, 3.6) | 2.1 (1.9, 2.3) | -35.0 (-42.2, -26.5) |
| Romania | 251 (238, 265) | 383 (353, 416) | 52.7 (38.5, 68.4) | 17.5 (16.6, 18.5) | 16.2 (14.9, 17.6) | -7.7 (-16.3, 1.8) | 1.9 (1.8, 2.0) | 2.2 (2.0, 2.4) | 14.0 (4.2, 25.3) |
| Moldova | 53 (49, 57) | 50 (45, 54) | -6.6 (-17.6, 5.9) | 24.9 (23.2, 26.8) | 16.2 (14.7, 17.7) | -35.1 (-42.8, -26.4) | 2.9 (2.7, 3.1) | 2.3 (2.1, 2.6) | -18.8 (-28.2, -8.3) |
| Bolivia | 22 (18, 27) | 69 (50, 89) | 217.3 (130.6, 323.9) | 14.7 (11.8, 17.9) | 15.9 (11.4, 20.4) | 8.2 (-21.4, 44.5) | 1.7 (1.3, 2.0) | 2.3 (1.7, 2.7) | 35.9 (6.4, 69.2) |
| Australia | 232 (219, 245) | 399 (352, 448) | 72.1 (49.8, 95.9) | 19.1 (18.1, 20.2) | 15.4 (13.6, 17.3) | -19.7 (-30.1, -8.6) | 3.1 (2.9, 3.2) | 3.1 (2.8, 3.4) | 1.0 (-9.4, 11.4) |
| Philippines | 464 (432, 498) | 446 (392, 507) | -3.9 (-16.6, 12.3) | 36.3 (33.8, 39.0) | 15.2 (13.4, 17.3) | -58.0 (-63.6, -51.0) | 5.0 (4.7, 5.3) | 1.9 (1.7, 2.0) | -62.5 (-65.9, -58.7) |
| Israel | 75 (70, 80) | 102 (93, 113) | 36.8 (22.9, 53.0) | 24.6 (22.8, 26.4) | 14.9 (13.6, 16.5) | -39.2 (-45.4, -32.0) | 4.0 (3.7, 4.3) | 3.0 (2.8, 3.3) | -24.4 (-31.5, -16.2) |
| El Salvador | 21 (17, 25) | 46 (37, 56) | 114.7 (69.2, 174.7) | 13.9 (11.0, 16.2) | 13.9 (11.3, 16.8) | 0.2 (-21.1, 28.1) | 2.3 (1.8, 2.6) | 2.2 (1.8, 2.6) | -2.8 (-20.5, 23.6) |
| Norway | 91 (88, 93) | 83 (78, 88) | -8.6 (-14.3, -2.1) | 19.1 (18.5, 19.6) | 13.8 (12.9, 14.6) | -27.8 (-32.3, -22.6) | 2.7 (2.7, 2.8) | 2.6 (2.4, 2.7) | -6.2 (-12.1, 0.7) |
| Maldives | 1 (1, 1) | 2 (1, 2) | 155.5 (82.3, 235.0) | 23.3 (17.3, 31.2) | 13.7 (11.4, 16.2) | -40.9 (-57.8, -22.5) | 2.7 (2.0, 3.6) | 2.9 (2.4, 3.4) | 6.9 (-22.9, 40.2) |
| Ecuador | 20 (19, 22) | 110 (97, 126) | 440.3 (360.0, 530.3) | 7.5 (6.8, 8.2) | 13.7 (12.0, 15.6) | 83.1 (55.9, 113.6) | 1.2 (1.1, 1.3) | 2.5 (2.2, 2.7) | 103.8 (78.4, 131.1) |
| Italy | 1332 (1273, 1391) | 1334 (1215, 1469) | 0.2 (-9.7, 10.7) | 24.0 (23.0, 25.1) | 13.5 (12.3, 14.8) | -44.0 (-49.5, -38.1) | 3.6 (3.5, 3.8) | 2.6 (2.4, 2.8) | -29.2 (-35.5, -22.3) |
| Honduras | 9 (7, 11) | 40 (29, 50) | 341.0 (226.5, 471.8) | 9.1 (7.3, 10.8) | 13.2 (9.4, 16.4) | 44.8 (7.2, 87.8) | 1.6 (1.3, 1.8) | 2.1 (1.5, 2.7) | 32.6 (-0.2, 67.9) |
| Tonga | 0 | 1 (0, 1) | 70.7 (39.2, 108.9) | 13.1 (11.5, 14.8) | 13.2 (11.0, 15.3) | 0.9 (-17.8, 23.4) | 1.6 (1.4, 1.8) | 1.6 (1.4, 1.8) | 1.6 (-13.5, 19.2) |
| Trinidad and Tobago | 8 (8, 9) | 12 (10, 15) | 47.6 (18.5, 80.9) | 17.5 (16.0, 19.4) | 12.8 (10.6, 15.6) | -26.8 (-41.2, -10.2) | 2.3 (2.1, 2.5) | 2.2 (1.9, 2.6) | -2.3 (-17.3, 15.8) |
| Morocco | 86 (71, 102) | 195 (160, 234) | 126.8 (77.7, 192.1) | 12.1 (10.0, 14.5) | 12.7 (10.4, 15.2) | 4.1 (-18.4, 34.1) | 1.5 (1.3, 1.8) | 1.8 (1.5, 2.0) | 14.6 (-7.3, 39.9) |
| Malta | 4 (4, 4) | 7 (7, 8) | 90.7 (65.1, 120.0) | 15.5 (14.2, 16.9) | 12.5 (11.3, 13.8) | -19.2 (-30.0, -6.8) | 2.2 (2.0, 2.4) | 2.4 (2.1, 2.6) | 9.7 (-4.6, 25.1) |
| Libya | 9 (7, 11) | 24 (16, 31) | 172.7 (104.8, 249.9) | 10.5 (8.0, 12.8) | 12.4 (8.1, 15.8) | 18.8 (-10.8, 52.4) | 1.5 (1.1, 1.8) | 1.8 (1.2, 2.2) | 18.5 (-7.2, 47.2) |
| Bermuda | 1 (1, 1) | 1 (1, 1) | 48.0 (27.6, 71.6) | 18.1 (16.4, 20.0) | 12.3 (10.9, 13.8) | -32.2 (-41.5, -21.3) | 2.6 (2.4, 2.9) | 2.5 (2.3, 2.8) | -2.7 (-15.3, 11.8) |
| American Samoa | 0 | 0 | 72.7 (40.2, 113.3) | 12.4 (10.7, 14.2) | 12.2 (10.2, 14.3) | -0.9 (-19.6, 22.4) | 1.6 (1.4, 1.9) | 1.7 (1.5, 1.9) | 3.5 (-13.4, 24.2) |
| Canada | 315 (298, 332) | 508 (456, 561) | 61.1 (43.7, 79.6) | 15.7 (14.8, 16.5) | 12.2 (11.0, 13.5) | -22.0 (-30.4, -13.0) | 2.6 (2.5, 2.7) | 2.5 (2.3, 2.8) | -3.2 (-13.3, 7.5) |
| Venezuela | 69 (63, 74) | 165 (137, 194) | 140.3 (94.7, 189.2) | 14.5 (13.4, 15.7) | 12.2 (10.1, 14.3) | -16.2 (-32.1, 0.9) | 2.3 (2.1, 2.5) | 2.2 (1.9, 2.6) | -1.7 (-18.3, 15.7) |
| Barbados | 3 (3, 3) | 3 (3, 4) | 18.6 (0.7, 36.9) | 13.7 (12.5, 15.1) | 12.0 (10.5, 13.5) | -12.8 (-26.0, 0.7) | 1.9 (1.8, 2.1) | 1.9 (1.7, 2.1) | -0.7 (-14.3, 13.9) |
| Mongolia | 8 (6, 12) | 9 (7, 15) | 21.5 (-3.8, 51.4) | 14.9 (11.6, 23.1) | 11.7 (9.3, 18.5) | -21.5 (-37.9, -2.2) | 1.5 (1.1, 2.2) | 1.6 (1.3, 2.4) | 7.2 (-14.5, 32.4) |
| Puerto Rico | 52 (48, 56) | 54 (47, 62) | 4.3 (-9.8, 21.8) | 23.0 (21.3, 24.8) | 11.6 (10.1, 13.2) | -49.7 (-56.5, -41.2) | 3.8 (3.5, 4.0) | 2.4 (2.1, 2.7) | -36.0 (-44.5, -26.3) |
| New Zealand | 39 (36, 42) | 55 (49, 61) | 41.3 (23.2, 61.1) | 15.7 (14.4, 17.1) | 11.5 (10.3, 12.9) | -26.5 (-35.9, -16.2) | 2.4 (2.2, 2.6) | 2.3 (2.1, 2.5) | -2.5 (-13.9, 10.3) |
| Dominica | 1 (0, 1) | 1 (1, 1) | 26.3 (5.8, 47.8) | 10.8 (9.8, 12.1) | 11.5 (9.9, 13.2) | 5.8 (-11.4, 23.8) | 1.5 (1.3, 1.6) | 1.5 (1.4, 1.7) | 3.5 (-12.5, 20.5) |
| Andorra | 1 (0, 1) | 1 (1, 1) | 76.4 (41.8, 120.1) | 15.6 (12.4, 19.3) | 11.3 (9.1, 15.0) | -27.5 (-41.7, -9.5) | 3.1 (2.6, 3.8) | 2.2 (1.7, 2.9) | -30.1 (-42.0, -14.6) |
| Uzbekistan | 62 (57, 68) | 89 (76, 100) | 42.5 (21.6, 65.9) | 11.5 (10.5, 12.5) | 11.3 (9.7, 12.8) | -1.5 (-16.0, 14.6) | 1.6 (1.4, 1.7) | 1.1 (1.0, 1.2) | -31.8 (-39.8, -21.4) |
| Grenada | 1 (1, 1) | 1 (1, 1) | 70.0 (46.3, 95.4) | 17.1 (15.6, 18.9) | 11.3 (10.1, 12.6) | -34.0 (-43.3, -24.2) | 1.8 (1.6, 1.9) | 1.7 (1.5, 1.8) | -5.8 (-19.0, 7.9) |
| Bangladesh | 260 (213, 361) | 693 (484, 820) | 166.3 (52.9, 254.1) | 11.8 (9.7, 16.4) | 11.2 (7.8, 13.3) | -5.1 (-45.5, 26.2) | 1.3 (1.1, 1.8) | 1.8 (1.3, 2.1) | 42.7 (-19.3, 87.4) |
| Cambodia | 25 (19, 32) | 53 (43, 63) | 114.7 (60.0, 180.6) | 13.4 (10.1, 17.0) | 11.1 (9.0, 13.1) | -17.1 (-38.2, 8.4) | 1.3 (1.0, 1.7) | 1.5 (1.2, 1.7) | 9.7 (-18.7, 43.5) |
| Azerbaijan | 14 (12, 16) | 37 (25, 44) | 159.9 (92.2, 217.3) | 6.4 (5.5, 7.2) | 10.7 (7.3, 12.7) | 66.2 (22.9, 102.9) | 0.8 (0.7, 0.9) | 1.1 (0.8, 1.3) | 35.4 (-0.2, 64.2) |
| Sao Tome and Principe | 0 | 0 (0, 1) | 92.5 (43.5, 141.8) | 7.0 (5.8, 10.1) | 10.4 (7.6, 15.5) | 49.4 (11.4, 87.7) | 0.9 (0.8, 1.3) | 1.2 (0.9, 1.8) | 37.6 (4.4, 71.7) |
| Turkmenistan | 10 (9, 11) | 15 (14, 17) | 51.2 (32.0, 71.9) | 12.0 (11.0, 13.1) | 10.2 (9.2, 11.3) | -14.7 (-25.5, -3.0) | 1.5 (1.4, 1.6) | 1.3 (1.2, 1.4) | -10.6 (-21.9, 1.7) |
| Myanmar | 119 (80, 164) | 203 (148, 240) | 70.7 (24.6, 128.1) | 12.3 (8.3, 17.0) | 10.2 (7.4, 12.1) | -17.2 (-39.6, 10.7) | 1.2 (0.8, 1.6) | 1.3 (0.9, 1.5) | 10.1 (-18.9, 42.0) |
| Egypt | 85 (62, 110) | 194 (121, 253) | 127.2 (68.3, 190.6) | 8.5 (6.2, 10.9) | 10.1 (6.3, 13.1) | 18.8 (-12.0, 51.9) | 1.0 (0.7, 1.2) | 1.2 (0.8, 1.6) | 25.9 (-4.8, 59.8) |
| Tunisia | 21 (18, 24) | 63 (48, 78) | 202.8 (124.8, 295.1) | 8.3 (7.1, 9.7) | 10.0 (7.7, 12.5) | 20.8 (-10.4, 57.6) | 1.2 (1.0, 1.4) | 1.5 (1.2, 1.7) | 25.5 (-3.3, 53.8) |
| Brazil | 620 (601, 640) | 1203 (1143, 1244) | 94.1 (82.8, 103.3) | 14.6 (14.2, 15.1) | 10.0 (9.5, 10.4) | -31.6 (-35.5, -28.3) | 2.2 (2.1, 2.2) | 1.8 (1.7, 1.9) | -16.1 (-21.0, -12.2) |
| Albania | 8 (7, 11) | 25 (20, 31) | 218.7 (126.7, 316.5) | 7.3 (6.3, 10.3) | 10.0 (8.1, 12.2) | 36.3 (-3.0, 78.1) | 1.1 (0.9, 1.6) | 1.7 (1.4, 2.0) | 52.7 (11.1, 89.7) |
| Costa Rica | 8 (7, 9) | 25 (22, 29) | 209.3 (157.4, 266.4) | 8.6 (7.8, 9.7) | 9.5 (8.2, 10.9) | 10.5 (-8.1, 30.9) | 1.5 (1.3, 1.7) | 1.9 (1.6, 2.2) | 27.1 (7.0, 48.3) |
| Mauritius | 5 (4, 5) | 7 (7, 8) | 49.7 (28.2, 76.5) | 14.8 (13.4, 16.4) | 9.3 (8.2, 10.5) | -37.6 (-46.6, -26.4) | 1.9 (1.7, 2.1) | 1.5 (1.3, 1.6) | -22.2 (-33.3, -8.8) |
| Paraguay | 8 (7, 10) | 24 (20, 31) | 201.6 (130.2, 289.7) | 7.2 (6.2, 8.9) | 9.2 (7.4, 11.6) | 28.6 (-1.8, 66.2) | 1.2 (1.0, 1.5) | 1.6 (1.3, 1.9) | 31.8 (2.8, 63.3) |
| Iran | 96 (86, 109) | 298 (275, 337) | 208.7 (165.1, 254.8) | 9.9 (8.8, 11.2) | 9.1 (8.4, 10.3) | -8.1 (-21.1, 5.6) | 1.6 (1.4, 1.8) | 1.5 (1.4, 1.7) | -7.6 (-20.4, 5.6) |
| Yemen | 15 (10, 22) | 48 (36, 63) | 212.7 (123.5, 334.9) | 7.5 (5.2, 10.8) | 8.8 (6.6, 11.6) | 16.8 (-16.5, 62.4) | 0.9 (0.7, 1.2) | 1.2 (1.0, 1.5) | 36.3 (5.2, 74.6) |
| United Arab Emirates | 1 (1, 1) | 3 (2, 4) | 286.4 (159.3, 435.8) | 7.2 (5.2, 9.4) | 8.8 (5.7, 11.3) | 21.5 (-18.5, 68.5) | 1.0 (0.7, 1.3) | 1.2 (0.8, 1.5) | 21.0 (-15.7, 59.3) |
| Panama | 8 (7, 9) | 19 (17, 21) | 139.1 (103.0, 179.7) | 9.9 (8.9, 11.0) | 8.5 (7.5, 9.6) | -14.2 (-27.2, 0.3) | 1.8 (1.6, 2.0) | 1.8 (1.6, 2.1) | 1.4 (-13.6, 18.1) |
| Lebanon | 8 (6, 10) | 26 (19, 31) | 208.7 (142.8, 284.6) | 8.9 (6.4, 11.0) | 8.4 (6.2, 9.9) | -6.0 (-26.1, 17.2) | 1.2 (0.8, 1.4) | 1.4 (1.1, 1.7) | 22.6 (-1.7, 50.5) |
| Sudan | 36 (26, 52) | 68 (52, 85) | 89.9 (33.3, 160.6) | 7.6 (5.6, 11.0) | 8.3 (6.4, 10.5) | 9.4 (-23.2, 50.1) | 0.9 (0.7, 1.3) | 1.2 (0.9, 1.4) | 27.7 (-4.6, 69.4) |
| United Kingdom | 823 (809, 836) | 692 (669, 710) | -15.9 (-19.2, -13.2) | 13.2 (13.0, 13.4) | 8.3 (8.0, 8.5) | -37.1 (-39.5, -35.1) | 1.8 (1.8, 1.8) | 1.5 (1.4, 1.5) | -18.9 (-21.9, -16.5) |
| Bahrain | 1 (1, 1) | 2 (2, 2) | 156.7 (85.9, 247.1) | 11.7 (9.2, 18.9) | 8.3 (7.1, 10.6) | -28.8 (-48.4, -3.8) | 1.2 (0.9, 1.9) | 1.6 (1.4, 2.1) | 36.5 (-0.9, 84.5) |
| Mexico | 165 (160, 170) | 489 (462, 508) | 197.1 (174.8, 211.9) | 7.7 (7.4, 7.9) | 8.2 (7.7, 8.5) | 6.5 (-1.5, 11.8) | 1.2 (1.1, 1.2) | 1.4 (1.4, 1.5) | 24.9 (15.9, 30.9) |
| Indonesia | 265 (227, 329) | 714 (652, 835) | 169.2 (109.9, 218.0) | 7.2 (6.2, 9.0) | 8.1 (7.4, 9.5) | 11.8 (-12.8, 32.0) | 0.9 (0.8, 1.1) | 1.1 (1.0, 1.2) | 17.1 (-8.5, 34.7) |
| Guatemala | 24 (22, 27) | 47 (41, 54) | 94.2 (65.1, 135.1) | 15.3 (13.7, 16.9) | 8.1 (7.0, 9.3) | -47.1 (-55.1, -36.0) | 1.8 (1.7, 2.0) | 1.4 (1.3, 1.6) | -23.3 (-33.9, -9.7) |
| Greece | 98 (91, 105) | 135 (122, 148) | 37.7 (22.7, 54.8) | 10.3 (9.6, 11.0) | 8.0 (7.3, 8.8) | -22.4 (-30.9, -12.8) | 1.6 (1.5, 1.7) | 1.4 (1.3, 1.5) | -13.7 (-22.2, -4.4) |
| Algeria | 43 (36, 51) | 123 (101, 142) | 187.1 (124.3, 255.2) | 6.9 (5.8, 8.2) | 7.8 (6.4, 9.0) | 13.1 (-11.6, 40.0) | 1.2 (1.0, 1.4) | 1.4 (1.2, 1.6) | 19.1 (-6.0, 47.3) |
| Tajikistan | 12 (10, 13) | 14 (12, 19) | 22.6 (2.7, 49.1) | 8.7 (7.7, 10.0) | 7.8 (6.6, 10.1) | -10.4 (-25.0, 8.9) | 1.2 (1.1, 1.4) | 1.0 (0.9, 1.3) | -13.4 (-26.4, 3.8) |
| Qatar | 0 | 1 (1, 2) | 361.2 (228.3, 546.0) | 10.6 (8.3, 14.5) | 7.7 (6.0, 10.8) | -27.4 (-48.3, 1.7) | 1.1 (0.9, 1.5) | 1.5 (1.3, 2.1) | 35.2 (2.2, 78.3) |
| Colombia | 62 (58, 68) | 225 (196, 260) | 260.2 (208.0, 324.6) | 7.5 (7.0, 8.2) | 7.4 (6.4, 8.5) | -2.4 (-16.5, 15.1) | 1.2 (1.1, 1.3) | 1.8 (1.6, 2.0) | 44.2 (25.5, 65.6) |
| Ireland | 26 (24, 29) | 32 (28, 36) | 21.1 (1.7, 44.4) | 9.8 (8.9, 10.8) | 7.2 (6.2, 8.2) | -27.0 (-38.7, -12.9) | 1.2 (1.1, 1.4) | 1.4 (1.2, 1.6) | 12.7 (-4.5, 33.5) |
| Jordan | 6 (5, 7) | 17 (14, 21) | 199.2 (132.4, 287.5) | 11.3 (8.9, 13.4) | 7.0 (5.9, 8.4) | -37.5 (-51.5, -19.1) | 1.7 (1.4, 2.0) | 1.6 (1.3, 1.8) | -9.5 (-28.1, 12.7) |
| Iraq | 37 (30, 47) | 70 (52, 81) | 85.9 (32.0, 149.0) | 10.2 (8.2, 12.9) | 7.0 (5.2, 8.1) | -31.8 (-51.6, -8.7) | 1.1 (0.9, 1.4) | 1.8 (1.4, 2.1) | 57.7 (11.3, 110.7) |
| The Bahamas | 1 (1, 1) | 1 (1, 1) | 108.5 (74.1, 148.5) | 7.1 (6.4, 8.0) | 6.9 (6.0, 7.8) | -3.9 (-19.8, 14.5) | 1.2 (1.0, 1.3) | 1.2 (1.0, 1.3) | 0.3 (-15.2, 19.0) |
| Oman | 2 (2, 3) | 5 (4, 6) | 126.7 (60.5, 213.4) | 7.0 (5.3, 9.2) | 6.8 (5.4, 8.3) | -1.7 (-30.4, 35.9) | 1.0 (0.8, 1.2) | 1.3 (1.0, 1.5) | 32.3 (-3.9, 72.8) |
| Palestine | 3 (3, 4) | 7 (6, 8) | 101.1 (59.3, 156.6) | 7.8 (6.5, 9.6) | 6.7 (5.8, 7.7) | -14.0 (-31.9, 9.8) | 1.0 (0.8, 1.1) | 1.1 (1.0, 1.3) | 16.3 (-6.5, 42.3) |
| Nicaragua | 6 (5, 8) | 15 (13, 22) | 169.4 (112.8, 228.3) | 7.5 (6.2, 10.6) | 6.7 (5.4, 9.5) | -10.5 (-29.3, 9.0) | 1.4 (1.2, 2.0) | 1.5 (1.2, 2.2) | 6.6 (-14.4, 28.8) |
| Saudi Arabia | 15 (10, 21) | 29 (20, 36) | 97.7 (10.8, 205.6) | 5.3 (3.7, 7.7) | 6.4 (4.4, 8.1) | 22.1 (-31.6, 88.7) | 0.7 (0.5, 1.0) | 1.1 (0.7, 1.4) | 53.3 (-11.6, 124.5) |
| Syria | 16 (13, 20) | 36 (29, 45) | 128.2 (62.5, 205.2) | 7.2 (5.9, 9.1) | 6.4 (5.1, 7.9) | -10.8 (-36.5, 19.2) | 0.9 (0.7, 1.1) | 1.0 (0.9, 1.2) | 17.9 (-13.6, 50.1) |
| Peru | 52 (43, 58) | 109 (91, 127) | 109.1 (71.5, 158.0) | 8.8 (7.3, 9.9) | 6.3 (5.2, 7.4) | -28.4 (-41.2, -11.6) | 1.5 (1.3, 1.7) | 1.4 (1.2, 1.6) | -8.9 (-22.5, 8.0) |
| Jamaica | 4 (4, 5) | 10 (8, 12) | 141.7 (91.3, 189.1) | 3.6 (3.2, 4.0) | 6.2 (5.0, 7.3) | 70.3 (34.9, 103.8) | 0.6 (0.5, 0.6) | 0.9 (0.7, 1.0) | 58.9 (25.7, 88.0) |
| Cyprus | 3 (3, 5) | 7 (6, 9) | 107.0 (50.9, 167.9) | 6.5 (5.4, 8.9) | 6.1 (5.1, 7.4) | -6.0 (-31.4, 21.7) | 1.0 (0.8, 1.3) | 1.3 (1.1, 1.5) | 33.2 (-3.0, 69.8) |
| Pakistan | 133 (110, 174) | 249 (202, 306) | 87.4 (34.7, 144.8) | 4.5 (3.8, 6.0) | 5.5 (4.5, 6.8) | 21.5 (-12.7, 58.7) | 0.5 (0.5, 0.7) | 0.6 (0.5, 0.7) | 16.9 (-14.7, 46.8) |
| Antigua and Barbuda | 0 | 0 | 31.3 (11.0, 54.0) | 6.4 (5.7, 7.1) | 5.4 (4.8, 6.1) | -15.3 (-28.4, -0.6) | 1.0 (0.9, 1.1) | 0.9 (0.8, 1.0) | -11.2 (-25.2, 3.2) |
| Turkey | 200 (141, 239) | 248 (214, 318) | 24.1 (-4.1, 98.1) | 12.8 (9.0, 15.3) | 5.3 (4.6, 6.8) | -58.4 (-67.9, -33.6) | 1.8 (1.3, 2.2) | 1.1 (1.0, 1.4) | -40.7 (-53.4, -4.8) |
| Kuwait | 1 (1, 1) | 4 (3, 4) | 386.6 (311.7, 481.4) | 3.4 (3.1, 3.8) | 3.9 (3.4, 4.5) | 14.1 (-3.5, 36.3) | 0.7 (0.7, 0.8) | 1.2 (1.0, 1.3) | 55.5 (34.2, 84.0) |

GBD = Global Burden of Disease, UI = Uncertainty interval. Death numbers (95% UI) estimated by GBD2017 were rounded to integers, which introduced 0.
