# Supplementary material for: Time Trends and Predictions of Suicide Mortality for People Aged 70 Years and Over From 1990 to 2030 Based on the Global Burden of Disease Study 2017
Source: Front Psychiatry. 2021 Sep 27;12:721343. doi: 10.3389/fpsyt.2021.721343 (PMC8502866; doi:10.3389/fpsyt.2021.721343)
Supplement: Supplementary S1 — Partial statistical methods used in the study. [file Data_Sheet_1.zip › Supplementary Table 2.docx]

**Supplementary Table 2. Mortality rates from suicide for the elderly (70+ years), age-standardized mortality rates from suicide for all ages, and their percentage differences, in 2017, for 21 GBD regions.**

| **Region** | **Mortality rate (per 100 000)** | | |
| --- | --- | --- | --- |
|  | **70+ years** | **Age-standardized** | **Percentage difference (%)** |
| Central Asia | 20.3 | 11.5 | 77.1 |
| Central Europe | 24.7 | 11.7 | 112.3 |
| Eastern Europe | 37.1 | 24.6 | 50.9 |
| Australasia | 14.8 | 11.1 | 33.3 |
| High-income Asia Pacific | 40.8 | 16.4 | 149.2 |
| High-income North America | 18.4 | 12.6 | 45.9 |
| Southern Latin America | 20.9 | 10.9 | 92.5 |
| Western Europe | 21.2 | 8.5 | 148.6 |
| Andean Latin America | 9.7 | 5.4 | 80.1 |
| Caribbean | 29.5 | 9.5 | 210.8 |
| Central Latin America | 8.7 | 6.2 | 39.5 |
| Tropical Latin America | 10.0 | 6.1 | 64.3 |
| North Africa and Middle East | 8.4 | 4.7 | 79.0 |
| South Asia | 25.0 | 13.5 | 85.6 |
| East Asia | 38.3 | 7.4 | 419.1 |
| Oceania | 20.5 | 21.1 | -2.7 |
| Southeast Asia | 16.8 | 6.1 | 174.2 |
| Central Sub-Saharan Africa | 50.1 | 12.0 | 318.1 |
| Eastern Sub-Saharan Africa | 56.6 | 11.0 | 412.3 |
| Southern Sub-Saharan Africa | 27.4 | 13.8 | 98.8 |
| Western Sub-Saharan Africa | 50.5 | 9.5 | 431.6 |

GBD = Global Burden of Disease. Percentage difference = 100 × (R_elderly_ － R_std_) / R_std_, where R_elderly_ donates mortality rate from suicide for the elderly aged 70 and older, and R_std_ donates age-standardized mortality rate from suicide for all ages.
