# Supplementary material for: Time Trends and Predictions of Suicide Mortality for People Aged 70 Years and Over From 1990 to 2030 Based on the Global Burden of Disease Study 2017
Source: Front Psychiatry. 2021 Sep 27;12:721343. doi: 10.3389/fpsyt.2021.721343 (PMC8502866; doi:10.3389/fpsyt.2021.721343)
Supplement: Supplementary S1 — Partial statistical methods used in the study. [file Data_Sheet_1.zip › Supplementary Table 3.docx]

**Supplementary Table 3. Mortality rates from suicide** **for the elderly (70+ years), age-standardized mortality rates from suicide for all ages, and their percentage differences, in 2017, for 195 countries and territories.**

| **Location** | **Mortality rate (per 100 000)** | | |
| --- | --- | --- | --- |
|  | **70+ years** | **Age-standardized** | **Percentage difference (%)** |
| Mongolia | 11.7 | 15.4 | -24.0 |
| Ireland | 7.2 | 8.4 | -14.5 |
| Papua New Guinea | 20.3 | 23.1 | -12.2 |
| Kiribati | 24.5 | 26.4 | -7.2 |
| New Zealand | 11.5 | 11.3 | 1.8 |
| Mauritius | 9.3 | 8.3 | 11.4 |
| Canada | 12.2 | 10.9 | 12.1 |
| United Kingdom | 8.3 | 7.4 | 13.0 |
| Nicaragua | 6.7 | 5.9 | 14.3 |
| Turkmenistan | 10.2 | 8.7 | 17.8 |
| Moldova | 16.2 | 13.6 | 18.6 |
| Solomon Islands | 19.6 | 16.4 | 19.4 |
| Poland | 16.8 | 13.9 | 20.2 |
| Uzbekistan | 11.3 | 9.4 | 20.5 |
| Costa Rica | 9.5 | 7.8 | 22.3 |
| Pakistan | 5.5 | 4.4 | 23.9 |
| Marshall Islands | 28.1 | 22.5 | 25.0 |
| Trinidad and Tobago | 12.8 | 10.1 | 26.9 |
| Ukraine | 33.8 | 25.6 | 31.8 |
| Cyprus | 6.1 | 4.6 | 31.8 |
| Vanuatu | 23.5 | 17.7 | 33.0 |
| Colombia | 7.4 | 5.5 | 33.2 |
| Tajikistan | 7.8 | 5.8 | 35.0 |
| Guatemala | 8.1 | 5.9 | 36.4 |
| Mexico | 8.2 | 5.9 | 37.8 |
| Australia | 15.4 | 11.0 | 39.1 |
| Venezuela | 12.2 | 8.7 | 40.5 |
| El Salvador | 13.9 | 9.8 | 41.7 |
| Swaziland | 31.7 | 22.3 | 42.1 |
| Andorra | 11.3 | 7.9 | 43.0 |
| Finland | 18.6 | 12.9 | 43.5 |
| Federated States of Micronesia | 24.6 | 17.0 | 44.1 |
| Yemen | 8.8 | 6.0 | 46.4 |
| Lithuania | 41.5 | 28.0 | 48.1 |
| Ecuador | 13.7 | 9.2 | 48.5 |
| Norway | 13.8 | 9.3 | 48.7 |
| Guam | 30.7 | 20.6 | 49.0 |
| United States | 19.2 | 12.8 | 49.8 |
| Greenland | 77.5 | 51.1 | 51.7 |
| Libya | 12.4 | 8.1 | 54.1 |
| Russian Federation | 38.8 | 25.1 | 54.5 |
| Kuwait | 3.9 | 2.5 | 57.6 |
| Paraguay | 9.2 | 5.8 | 60.1 |
| Turkey | 5.3 | 3.3 | 61.0 |
| Kazakhstan | 37.1 | 23.0 | 61.5 |
| Kyrgyzstan | 16.3 | 10.0 | 63.2 |
| Brazil | 10.0 | 6.1 | 64.6 |
| Sudan | 8.3 | 5.0 | 66.0 |
| Qatar | 7.7 | 4.6 | 66.1 |
| Iran | 9.1 | 5.4 | 67.7 |
| South Africa | 18.8 | 11.2 | 68.3 |
| Thailand | 17.7 | 10.4 | 69.7 |
| Iraq | 7.0 | 4.1 | 70.2 |
| Panama | 8.5 | 5.0 | 70.5 |
| Jamaica | 6.2 | 3.6 | 71.1 |
| United Arab Emirates | 8.8 | 5.1 | 72.6 |
| Chile | 17.6 | 10.1 | 74.0 |
| Lebanon | 8.4 | 4.8 | 75.5 |
| Sweden | 19.5 | 11.1 | 75.9 |
| Romania | 16.2 | 9.2 | 76.8 |
| Morocco | 12.7 | 7.1 | 77.0 |
| Fiji | 17.6 | 9.8 | 78.6 |
| Latvia | 30.8 | 17.0 | 81.4 |
| American Samoa | 12.2 | 6.7 | 81.6 |
| India | 28.5 | 15.6 | 83.0 |
| Egypt | 10.1 | 5.5 | 84.5 |
| Bangladesh | 11.2 | 6.0 | 85.9 |
| Guyana | 49.1 | 26.1 | 88.1 |
| Algeria | 7.8 | 4.1 | 88.9 |
| Puerto Rico | 11.6 | 6.1 | 89.8 |
| Palestine | 6.7 | 3.5 | 90.0 |
| Argentina | 20.3 | 10.6 | 91.0 |
| Slovakia | 18.5 | 9.5 | 94.8 |
| Albania | 10.0 | 5.1 | 95.1 |
| Bahrain | 8.3 | 4.2 | 96.5 |
| Peru | 6.3 | 3.1 | 100.2 |
| Myanmar | 10.2 | 5.0 | 102.6 |
| Laos | 17.1 | 8.4 | 103.1 |
| Antigua and Barbuda | 5.4 | 2.7 | 103.1 |
| Netherlands | 18.7 | 9.2 | 103.7 |
| Belgium | 29.1 | 14.3 | 103.7 |
| Japan | 32.4 | 15.6 | 106.9 |
| Syria | 6.4 | 3.1 | 107.5 |
| Estonia | 26.5 | 12.7 | 108.2 |
| Belarus | 40.6 | 19.0 | 113.2 |
| Nepal | 17.9 | 8.4 | 113.8 |
| The Bahamas | 6.9 | 3.2 | 116.1 |
| Saudi Arabia | 6.4 | 2.9 | 117.5 |
| Czech Republic | 23.5 | 10.8 | 118.1 |
| Jordan | 7.0 | 3.2 | 118.9 |
| Tonga | 13.2 | 6.0 | 120.7 |
| Suriname | 55.0 | 24.8 | 121.7 |
| Namibia | 26.8 | 11.8 | 127.1 |
| Greece | 8.0 | 3.5 | 128.6 |
| North Korea | 25.4 | 11.1 | 129.7 |
| Uruguay | 38.9 | 16.8 | 131.0 |
| Grenada | 11.3 | 4.8 | 132.7 |
| Northern Mariana Islands | 28.4 | 12.2 | 133.6 |
| Oman | 6.8 | 2.9 | 134.3 |
| Samoa | 23.6 | 10.0 | 136.2 |
| Belize | 18.5 | 7.8 | 136.5 |
| Afghanistan | 22.2 | 9.2 | 141.9 |
| Cambodia | 11.1 | 4.5 | 144.9 |
| Bolivia | 15.9 | 6.4 | 148.3 |
| Saint Vincent and the Grenadines | 17.9 | 7.2 | 148.7 |
| Timor-Leste | 18.4 | 7.4 | 149.7 |
| Dominica | 11.5 | 4.5 | 152.4 |
| Philippines | 15.2 | 6.0 | 153.6 |
| Haiti | 21.8 | 8.6 | 155.3 |
| Denmark | 22.5 | 8.8 | 156.2 |
| Dominican Republic | 22.9 | 8.9 | 156.3 |
| Lesotho | 82.1 | 31.7 | 158.8 |
| Indonesia | 8.1 | 3.1 | 159.0 |
| Israel | 14.9 | 5.7 | 160.7 |
| Saint Lucia | 18.0 | 6.9 | 161.4 |
| Azerbaijan | 10.7 | 4.1 | 161.9 |
| Seychelles | 21.2 | 8.0 | 164.3 |
| France | 33.4 | 12.4 | 169.3 |
| Brunei | 16.9 | 6.2 | 171.3 |
| Georgia | 20.6 | 7.5 | 174.3 |
| Malta | 12.5 | 4.5 | 176.6 |
| Hungary | 39.7 | 14.3 | 177.9 |
| Italy | 13.5 | 4.8 | 182.9 |
| Slovenia | 40.8 | 14.4 | 183.9 |
| Botswana | 28.9 | 10.0 | 188.8 |
| Germany | 28.8 | 9.9 | 190.1 |
| Cape Verde | 48.7 | 16.8 | 190.2 |
| Vietnam | 21.7 | 7.4 | 192.1 |
| Switzerland | 27.4 | 9.3 | 194.0 |
| Sao Tome and Principe | 10.4 | 3.5 | 195.1 |
| Honduras | 13.2 | 4.4 | 199.6 |
| Spain | 16.6 | 5.5 | 201.0 |
| Barbados | 12.0 | 4.0 | 201.2 |
| Taiwan | 40.9 | 13.5 | 202.2 |
| Bhutan | 17.4 | 5.7 | 203.7 |
| Iceland | 29.2 | 9.6 | 204.3 |
| Bosnia and Herzegovina | 21.5 | 6.8 | 213.7 |
| Sri Lanka | 62.5 | 19.8 | 215.8 |
| Zimbabwe | 84.4 | 26.4 | 219.8 |
| Tunisia | 10.0 | 3.1 | 222.9 |
| Central African Republic | 54.7 | 16.9 | 223.4 |
| Macedonia | 22.1 | 6.8 | 225.6 |
| Luxembourg | 28.3 | 8.7 | 225.7 |
| Austria | 36.5 | 11.2 | 226.2 |
| Bulgaria | 31.0 | 9.5 | 227.5 |
| Singapore | 24.1 | 7.3 | 228.4 |
| Montenegro | 30.9 | 9.4 | 229.3 |
| Croatia | 37.1 | 11.1 | 235.3 |
| Virgin Islands, U.S. | 21.4 | 6.3 | 239.8 |
| Eritrea | 54.9 | 15.7 | 249.6 |
| Bermuda | 12.3 | 3.4 | 258.7 |
| Malaysia | 31.1 | 7.8 | 296.2 |
| Guinea-Bissau | 54.7 | 13.6 | 302.3 |
| Serbia | 51.7 | 12.7 | 307.8 |
| Maldives | 13.7 | 3.3 | 310.9 |
| Democratic Republic of the Congo | 47.8 | 11.6 | 312.9 |
| South Korea | 86.0 | 20.8 | 313.5 |
| Cuba | 46.8 | 11.3 | 314.1 |
| Portugal | 32.0 | 7.7 | 316.1 |
| Armenia | 34.4 | 8.3 | 316.1 |
| Congo | 59.1 | 14.0 | 320.7 |
| Togo | 51.7 | 12.3 | 321.5 |
| Gabon | 53.1 | 12.5 | 323.6 |
| Mozambique | 77.0 | 17.2 | 347.8 |
| Madagascar | 49.4 | 11.0 | 349.6 |
| Somalia | 44.7 | 9.9 | 350.0 |
| Angola | 54.8 | 12.0 | 358.0 |
| Niger | 35.9 | 7.6 | 370.5 |
| Burkina Faso | 63.2 | 13.2 | 378.1 |
| Cameroon | 67.2 | 14.0 | 378.8 |
| Ghana | 46.5 | 9.7 | 379.4 |
| Equatorial Guinea | 45.0 | 9.3 | 381.8 |
| Mali | 32.9 | 6.8 | 383.6 |
| Sierra Leone | 47.9 | 9.9 | 385.1 |
| The Gambia | 47.1 | 9.7 | 385.3 |
| Benin | 54.4 | 11.1 | 388.4 |
| Comoros | 40.8 | 8.3 | 390.7 |
| Guinea | 55.0 | 11.1 | 394.8 |
| Malawi | 60.2 | 12.1 | 397.1 |
| Burundi | 62.8 | 12.6 | 398.0 |
| Djibouti | 42.6 | 8.4 | 404.7 |
| Chad | 55.8 | 11.0 | 406.0 |
| Zambia | 66.1 | 13.1 | 406.5 |
| South Sudan | 61.1 | 12.0 | 408.7 |
| Cote d'Ivoire | 70.5 | 13.9 | 408.9 |
| Kenya | 56.5 | 11.1 | 410.7 |
| Liberia | 70.1 | 13.2 | 430.7 |
| China | 38.5 | 7.2 | 434.2 |
| Tanzania | 47.5 | 8.8 | 438.8 |
| Mauritania | 35.8 | 6.6 | 439.0 |
| Rwanda | 60.3 | 11.0 | 451.0 |
| Ethiopia | 53.3 | 9.6 | 453.0 |
| Senegal | 71.6 | 12.9 | 454.6 |
| Uganda | 69.0 | 11.5 | 497.9 |
| Nigeria | 45.7 | 7.5 | 505.7 |

GBD = Global Burden of Disease. Percentage difference = 100 × (R_elderly_ － R_std_) / R_std_, where R_elderly_ donates mortality rate from suicide for the elderly aged 70 and older, and R_std_ donates age-standardized mortality rate from suicide for all ages.
