# Supplementary material for: Time Trends and Predictions of Suicide Mortality for People Aged 70 Years and Over From 1990 to 2030 Based on the Global Burden of Disease Study 2017
Source: Front Psychiatry. 2021 Sep 27;12:721343. doi: 10.3389/fpsyt.2021.721343 (PMC8502866; doi:10.3389/fpsyt.2021.721343)
Supplement: Supplementary S1 — Partial statistical methods used in the study. [file Data_Sheet_1.zip › Supplementary Table 4.docx]

**Supplementary Table 4. Percentage changes in mortality rates from suicide** **for the elderly (70+ years) between 1990 and 2017, percentage changes in age-standardized mortality rates from suicide for all ages between 1990 and 2017, and their differences, in 21 GBD regions.**

| **Region** | **Percentage change in mortality rate** | | |
| --- | --- | --- | --- |
|  | **70+ years** | **Age-standardized** | **Difference** |
| Central Asia | 5.1 | 0.5 | 4.6 |
| Central Europe | -37.9 | -26.7 | -11.2 |
| Eastern Europe | -13.1 | -4.0 | -9.1 |
| Australasia | -20.4 | -13.9 | -6.5 |
| High-income Asia Pacific | -19.2 | 10.7 | -29.8 |
| High-income North America | -19.5 | 3.5 | -23.0 |
| Southern Latin America | -39.0 | -19.7 | -19.3 |
| Western Europe | -38.9 | -33.7 | -5.1 |
| Andean Latin America | 4.4 | 3.6 | 0.8 |
| Caribbean | -19.6 | -26.1 | 6.4 |
| Central Latin America | -4.0 | 16.2 | -20.2 |
| Tropical Latin America | -30.8 | -14.0 | -16.7 |
| North Africa and Middle East | -18.8 | -17.4 | -1.4 |
| South Asia | 20.3 | -23.8 | 44.1 |
| East Asia | -46.1 | -64.1 | 18.1 |
| Oceania | -7.4 | -14.0 | 6.6 |
| Southeast Asia | -26.5 | -35.3 | 8.7 |
| Central Sub-Saharan Africa | -11.9 | -10.3 | -1.7 |
| Eastern Sub-Saharan Africa | -21.3 | -26.7 | 5.4 |
| Southern Sub-Saharan Africa | -0.8 | -25.3 | 24.5 |
| Western Sub-Saharan Africa | 5.7 | 0.8 | 4.9 |

GBD = Global Burden of Disease. Difference = P_elderly_ － P_std_, where P_elderly_ donates percentage change in mortality rate from suicide for the elderly between 1990 and 2017, and R_std_ donates percentage change in age-standardized mortality rate from suicide for all ages between 1990 and 2017.
