# Supplementary material for: Time Trends and Predictions of Suicide Mortality for People Aged 70 Years and Over From 1990 to 2030 Based on the Global Burden of Disease Study 2017
Source: Front Psychiatry. 2021 Sep 27;12:721343. doi: 10.3389/fpsyt.2021.721343 (PMC8502866; doi:10.3389/fpsyt.2021.721343)
Supplement: Supplementary S1 — Partial statistical methods used in the study. [file Data_Sheet_1.zip › Supplementary Table 5.docx]

**Supplementary Table 5. Percentage changes in mortality rates from suicide** **for the elderly (70+ years) between 1990 and 2017, percentage changes in age-standardized mortality rates from suicide for all ages between 1990 and 2017, and their differences, in 195 countries and territories.**

| **Location** | **Percentage change in mortality rate** | | |
| --- | --- | --- | --- |
|  | **70+ years** | **Age-standardized** | **Difference** |
| Jamaica | 70.3 | 131.7 | -61.4 |
| Argentina | -36.6 | 7.1 | -43.8 |
| Taiwan | -7.1 | 34.6 | -41.7 |
| Mexico | 6.5 | 47.6 | -41.1 |
| Japan | -39.2 | 0.8 | -40.1 |
| Ukraine | -6.1 | 31.9 | -38.0 |
| Turkey | -58.4 | -20.8 | -37.6 |
| Venezuela | -16.2 | 17.3 | -33.5 |
| Belize | -2.8 | 28.1 | -30.9 |
| Swaziland | -6.4 | 23.9 | -30.3 |
| Uruguay | 28.1 | 55.5 | -27.4 |
| Guyana | -6.5 | 18.4 | -24.9 |
| United States | -18.5 | 6.3 | -24.8 |
| Paraguay | 28.6 | 52.3 | -23.6 |
| Greece | -22.4 | 0.4 | -22.9 |
| Zimbabwe | 25.7 | 48.6 | -22.9 |
| Guam | -0.4 | 21.0 | -21.5 |
| Mongolia | -21.5 | -0.7 | -20.8 |
| Belgium | -37.5 | -18.9 | -18.6 |
| Antigua and Barbuda | -15.3 | 2.2 | -17.5 |
| Brazil | -31.6 | -15.0 | -16.6 |
| United Kingdom | -37.1 | -21.4 | -15.7 |
| Czech Republic | -58.8 | -43.1 | -15.6 |
| Suriname | -12.3 | 3.1 | -15.4 |
| Bulgaria | -50.3 | -35.8 | -14.5 |
| Israel | -39.2 | -25.7 | -13.5 |
| Malta | -19.2 | -5.7 | -13.5 |
| Moldova | -35.1 | -21.7 | -13.4 |
| Lesotho | 18.9 | 31.5 | -12.5 |
| New Zealand | -26.5 | -14.0 | -12.5 |
| Kazakhstan | -3.3 | 8.8 | -12.1 |
| Colombia | -2.4 | 9.5 | -11.9 |
| Netherlands | -28.1 | -16.2 | -11.8 |
| Costa Rica | 10.5 | 22.3 | -11.8 |
| Turkmenistan | -14.7 | -4.2 | -10.5 |
| France | -52.1 | -41.7 | -10.4 |
| Puerto Rico | -49.7 | -39.4 | -10.3 |
| Kenya | -12.7 | -2.4 | -10.3 |
| Italy | -44.0 | -33.7 | -10.3 |
| Spain | -35.8 | -25.6 | -10.2 |
| Central African Republic | -8.1 | 1.7 | -9.8 |
| Mozambique | -0.7 | 8.7 | -9.4 |
| Ireland | -27.0 | -18.4 | -8.6 |
| Malawi | -9.2 | -0.7 | -8.5 |
| Somalia | -16.1 | -7.8 | -8.3 |
| Kiribati | -7.1 | 1.1 | -8.2 |
| Tajikistan | -10.4 | -2.9 | -7.5 |
| Latvia | -41.3 | -34.2 | -7.1 |
| Panama | -14.2 | -7.3 | -7.0 |
| Grenada | -34.0 | -27.0 | -7.0 |
| Singapore | -56.7 | -49.8 | -6.9 |
| Cyprus | -6.0 | 0.5 | -6.5 |
| Libya | 18.8 | 25.1 | -6.4 |
| Macedonia | -25.2 | -18.8 | -6.4 |
| Democratic Republic of the Congo | -11.3 | -4.9 | -6.4 |
| Australia | -19.7 | -13.7 | -6.0 |
| North Korea | -17.7 | -11.7 | -5.9 |
| Togo | 7.3 | 13.1 | -5.8 |
| Russian Federation | -17.1 | -11.4 | -5.6 |
| Saint Lucia | -21.2 | -16.5 | -4.7 |
| Canada | -22.0 | -17.4 | -4.6 |
| Saint Vincent and the Grenadines | 4.4 | 8.9 | -4.5 |
| Romania | -7.7 | -3.3 | -4.4 |
| Tonga | 0.9 | 5.1 | -4.3 |
| Uzbekistan | -1.5 | 2.5 | -4.0 |
| Thailand | -24.0 | -20.2 | -3.7 |
| Chile | -59.9 | -56.3 | -3.6 |
| Philippines | -58.0 | -54.7 | -3.4 |
| Bahrain | -28.8 | -25.5 | -3.3 |
| Eritrea | -24.3 | -21.2 | -3.1 |
| Peru | -28.4 | -25.8 | -2.5 |
| Brunei | 37.6 | 39.9 | -2.3 |
| Guatemala | -47.1 | -45.1 | -2.0 |
| Burkina Faso | -6.0 | -4.0 | -2.0 |
| Sweden | -39.8 | -38.2 | -1.6 |
| Marshall Islands | -18.5 | -16.9 | -1.5 |
| Mauritius | -37.6 | -36.4 | -1.2 |
| Uganda | -12.6 | -11.4 | -1.2 |
| South Sudan | -7.3 | -6.1 | -1.2 |
| Hungary | -58.7 | -57.8 | -0.9 |
| Zambia | -21.5 | -20.7 | -0.8 |
| Dominica | 5.8 | 6.5 | -0.7 |
| Guinea-Bissau | -0.4 | 0.1 | -0.5 |
| Saudi Arabia | 22.1 | 22.3 | -0.3 |
| Germany | -27.7 | -27.5 | -0.2 |
| Croatia | -45.1 | -45.7 | 0.6 |
| The Bahamas | -3.9 | -4.5 | 0.6 |
| Andorra | -27.5 | -28.2 | 0.7 |
| American Samoa | -0.9 | -1.6 | 0.7 |
| Afghanistan | -10.2 | -11.0 | 0.8 |
| Trinidad and Tobago | -26.8 | -27.9 | 1.1 |
| Kyrgyzstan | -40.2 | -41.4 | 1.2 |
| Lithuania | 1.7 | 0.1 | 1.6 |
| Fiji | -0.1 | -1.8 | 1.7 |
| Benin | 9.0 | 6.6 | 2.4 |
| Poland | 5.7 | 2.8 | 2.9 |
| Tanzania | -11.5 | -14.7 | 3.2 |
| Sri Lanka | -48.2 | -51.5 | 3.3 |
| Lebanon | -6.0 | -9.3 | 3.4 |
| Dominican Republic | 80.4 | 76.8 | 3.5 |
| Madagascar | -21.0 | -24.7 | 3.7 |
| United Arab Emirates | 21.5 | 17.4 | 4.1 |
| Cote d'Ivoire | 6.5 | 2.4 | 4.1 |
| Gabon | 1.3 | -3.3 | 4.5 |
| Barbados | -12.8 | -17.9 | 5.1 |
| Seychelles | -42.1 | -47.2 | 5.1 |
| Malaysia | -13.8 | -19.1 | 5.3 |
| Syria | -10.8 | -16.2 | 5.4 |
| Angola | -19.5 | -25.2 | 5.7 |
| Vanuatu | -13.4 | -19.4 | 5.9 |
| Cameroon | 14.3 | 8.3 | 6.0 |
| Nigeria | -5.3 | -11.5 | 6.1 |
| Senegal | 5.1 | -1.3 | 6.4 |
| Denmark | -56.5 | -63.1 | 6.6 |
| The Gambia | 22.1 | 15.2 | 6.9 |
| Rwanda | -43.6 | -50.6 | 7.0 |
| Sierra Leone | 35.5 | 28.3 | 7.3 |
| Federated States of Micronesia | -15.5 | -22.9 | 7.3 |
| Guinea | 45.0 | 37.7 | 7.3 |
| Solomon Islands | -18.8 | -26.3 | 7.5 |
| Nicaragua | -10.5 | -18.4 | 7.8 |
| Chad | 37.0 | 29.1 | 7.9 |
| Virgin Islands, U.S. | 1.9 | -6.2 | 8.1 |
| Albania | 36.3 | 28.2 | 8.1 |
| Bermuda | -32.2 | -40.4 | 8.3 |
| Estonia | -44.2 | -52.5 | 8.3 |
| Niger | 6.4 | -2.1 | 8.5 |
| Austria | -34.9 | -43.6 | 8.7 |
| Slovakia | -29.8 | -38.7 | 8.9 |
| Jordan | -37.5 | -46.4 | 8.9 |
| Iceland | -23.2 | -32.5 | 9.2 |
| Georgia | 65.1 | 55.7 | 9.4 |
| Egypt | 18.8 | 8.6 | 10.1 |
| Norway | -27.8 | -38.3 | 10.5 |
| Djibouti | 1.2 | -9.3 | 10.5 |
| Qatar | -27.4 | -38.2 | 10.8 |
| Switzerland | -43.7 | -54.9 | 11.2 |
| Papua New Guinea | -9.1 | -20.7 | 11.6 |
| Slovenia | -36.3 | -48.1 | 11.8 |
| Congo | -5.9 | -17.8 | 11.9 |
| Comoros | -10.3 | -22.7 | 12.4 |
| Finland | -39.1 | -52.0 | 12.9 |
| Burundi | -24.9 | -37.8 | 12.9 |
| Pakistan | 21.5 | 8.5 | 13.0 |
| Namibia | -19.7 | -33.3 | 13.6 |
| Cambodia | -17.1 | -30.8 | 13.7 |
| Serbia | -20.5 | -34.8 | 14.2 |
| Liberia | 33.4 | 19.0 | 14.4 |
| Morocco | 4.1 | -10.9 | 15.0 |
| Ethiopia | -35.0 | -50.1 | 15.2 |
| Ghana | 37.3 | 21.9 | 15.4 |
| Mali | 5.7 | -10.5 | 16.2 |
| Ecuador | 83.1 | 66.7 | 16.3 |
| Iran | -8.1 | -24.6 | 16.4 |
| Timor-Leste | -25.4 | -42.4 | 17.0 |
| Portugal | -27.9 | -45.1 | 17.2 |
| Laos | -36.0 | -53.3 | 17.3 |
| Haiti | -5.6 | -23.0 | 17.4 |
| Northern Mariana Islands | 18.5 | 0.8 | 17.7 |
| Iraq | -31.8 | -49.7 | 17.9 |
| Myanmar | -17.2 | -35.2 | 18.0 |
| Luxembourg | -27.9 | -45.9 | 18.0 |
| Sao Tome and Principe | 49.4 | 31.0 | 18.4 |
| China | -46.7 | -65.6 | 18.9 |
| Equatorial Guinea | -28.2 | -47.3 | 19.0 |
| Palestine | -14.0 | -33.6 | 19.6 |
| Oman | -1.7 | -21.5 | 19.7 |
| Tunisia | 20.8 | 0.9 | 19.8 |
| Cape Verde | 52.7 | 32.8 | 20.0 |
| Cuba | -23.0 | -43.1 | 20.1 |
| Vietnam | -1.5 | -21.9 | 20.4 |
| El Salvador | 0.2 | -20.5 | 20.7 |
| Montenegro | 11.8 | -12.1 | 23.9 |
| Bhutan | -8.8 | -33.0 | 24.2 |
| Mauritania | 10.3 | -14.0 | 24.3 |
| Kuwait | 14.1 | -10.7 | 24.8 |
| Yemen | 16.8 | -9.4 | 26.2 |
| Maldives | -40.9 | -68.2 | 27.3 |
| Bosnia and Herzegovina | -3.7 | -31.5 | 27.8 |
| Algeria | 13.1 | -14.8 | 27.9 |
| Sudan | 9.4 | -18.5 | 27.9 |
| Bolivia | 8.2 | -21.1 | 29.3 |
| Samoa | -2.8 | -34.9 | 32.0 |
| Indonesia | 11.8 | -22.3 | 34.1 |
| Botswana | -2.7 | -36.8 | 34.1 |
| Bangladesh | -5.1 | -42.6 | 37.5 |
| Azerbaijan | 66.2 | 28.3 | 38.0 |
| India | 15.3 | -22.8 | 38.1 |
| South Africa | 2.9 | -39.9 | 42.8 |
| Greenland | -0.5 | -48.3 | 47.8 |
| Nepal | 19.7 | -29.3 | 49.0 |
| Belarus | 42.9 | -6.4 | 49.3 |
| Honduras | 44.8 | -25.3 | 70.2 |
| Armenia | 241.8 | 141.8 | 100.0 |
| South Korea | 205.8 | 89.7 | 116.1 |

GBD = Global Burden of Disease. Difference = P_elderly_ － P_std_, where P_elderly_ donates percentage change in mortality rate from suicide for the elderly between 1990 and 2017, and R_std_ donates percentage change in age-standardized mortality rate from suicide for all ages between 1990 and 2017.
