# Supplementary material for: Time Trends and Predictions of Suicide Mortality for People Aged 70 Years and Over From 1990 to 2030 Based on the Global Burden of Disease Study 2017
Source: Front Psychiatry. 2021 Sep 27;12:721343. doi: 10.3389/fpsyt.2021.721343 (PMC8502866; doi:10.3389/fpsyt.2021.721343)
Supplement: Supplementary S1 — Partial statistical methods used in the study. [file Data_Sheet_1.zip › Supplementary Table 6.docx]

**Supplementary Table 6. The orders and coefficients of ARIMA models for globe, sexes, 21 GBD regions, and 195 countries and territories.**

| **Location** | **ARIMA (*p*, *d*, *q*)** | **AR (1)** | | **AR (2)** | | **AR (3)** | | **AR (4)** | | **MA (1)** | | **MA (2)** | | **MA (3)** | | **Drift** | | **Intercept** | |
| --- | --- | --- | --- | --- | --- | --- | --- | --- | --- | --- | --- | --- | --- | --- | --- | --- | --- | --- | --- |
|  |  | **estimate** | **std.error** | **estimate** | **std.error** | **estimate** | **std.error** | **estimate** | **std.error** | **estimate** | **std.error** | **estimate** | **std.error** | **estimate** | **std.error** | **estimate** | **std.error** | **estimate** | **std.error** |
| **Global** | ARIMA(0,1,1) with drift | - | - | - | - | - | - | - | - | 0.605 | 0.156 | - | - | - | - | -0.397 | 0.137 | - | - |
| **Sex** | | | | | | | | | | | | | | | | | | | |
| Female | ARIMA(0,1,1) with drift | - | - | - | - | - | - | - | - | 0.764 | 0.15 | - | - | - | - | -0.379 | 0.139 | - | - |
| Male | ARIMA(0,1,1) with drift | - | - | - | - | - | - | - | - | 0.49 | 0.152 | - | - | - | - | -0.478 | 0.145 | - | - |
| **Region** | | | | | | | | | | | | | | | | | | | |
| Central Asia | ARIMA(0,1,0) | - | - | - | - | - | - | - | - | - | - | - | - | - | - | - | - | - | - |
| Central Europe | ARIMA(0,2,2) | - | - | - | - | - | - | - | - | -1.336 | 0.139 | 0.781 | 0.232 | - | - | - | - | - | - |
| Eastern Europe | ARIMA(0,1,1) | - | - | - | - | - | - | - | - | 0.507 | 0.184 | - | - | - | - | - | - | - | - |
| Australasia | ARIMA(0,2,1) | - | - | - | - | - | - | - | - | -0.661 | 0.138 | - | - | - | - | - | - | - | - |
| High-income Asia Pacific | ARIMA(2,0,0) with non-zero mean | 1.599 | 0.142 | -0.692 | 0.158 | - | - | - | - | - | - | - | - | - | - | - | - | 44.2 | 1.57 |
| High-income North America | ARIMA(0,2,1) | - | - | - | - | - | - | - | - | -0.542 | 0.15 | - | - | - | - | - | - | - | - |
| Southern Latin America | ARIMA(1,1,0) with drift | 0.341 | 0.18 | - | - | - | - | - | - | - | - | - | - | - | - | -0.485 | 0.14 | - | - |
| Western Europe | ARIMA(0,2,1) | - | - | - | - | - | - | - | - | -0.59 | 0.136 | - | - | - | - | - | - | - | - |
| Andean Latin America | ARIMA(2,0,0) with non-zero mean | 1.642 | 0.114 | -0.776 | 0.128 | - | - | - | - | - | - | - | - | - | - | - | - | 10.489 | 0.244 |
| Caribbean | ARIMA(1,1,0) | 0.611 | 0.146 | - | - | - | - | - | - | - | - | - | - | - | - | - | - | - | - |
| Central Latin America | ARIMA(0,2,1) | - | - | - | - | - | - | - | - | -0.465 | 0.163 | - | - | - | - | - | - | - | - |
| Tropical Latin America | ARIMA(0,2,0) | - | - | - | - | - | - | - | - | - | - | - | - | - | - | - | - | - | - |
| North Africa and Middle East | ARIMA(1,1,1) with drift | 0.166 | 0.212 | - | - | - | - | - | - | 0.935 | 0.203 | - | - | - | - | -0.064 | 0.031 | - | - |
| South Asia | ARIMA(0,1,0) with drift | - | - | - | - | - | - | - | - | - | - | - | - | - | - | 0.156 | 0.074 | - | - |
| East Asia | ARIMA(2,1,0) with drift | 0.765 | 0.169 | -0.51 | 0.163 | - | - | - | - | - | - | - | - | - | - | -1.181 | 0.466 | - | - |
| Oceania | ARIMA(0,1,1) with drift | - | - | - | - | - | - | - | - | 0.551 | 0.192 | - | - | - | - | -0.061 | 0.019 | - | - |
| Southeast Asia | ARIMA(0,2,0) | - | - | - | - | - | - | - | - | - | - | - | - | - | - | - | - | - | - |
| Central Sub-Saharan Africa | ARIMA(1,1,0) with drift | 0.314 | 0.181 | - | - | - | - | - | - | - | - | - | - | - | - | -0.25 | 0.095 | - | - |
| Eastern Sub-Saharan Africa | ARIMA(0,2,0) | - | - | - | - | - | - | - | - | - | - | - | - | - | - | - | - | - | - |
| Southern Sub-Saharan Africa | ARIMA(1,0,2) with non-zero mean | 0.946 | 0.049 | - | - | - | - | - | - | 0.766 | 0.204 | 0.33 | 0.137 | - | - | - | - | 29.497 | 3.413 |
| Western Sub-Saharan Africa | ARIMA(3,0,0) with non-zero mean | 1.519 | 0.181 | -0.252 | 0.35 | -0.357 | 0.186 | - | - | - | - | - | - | - | - | - | - | 51.121 | 0.45 |
| **Countries and territories** | | | | | | | | | | | | | | | | | | | |
| Afghanistan | (0,2,0) | - | - | - | - | - | - | - | - | - | - | - | - | - | - | - | - | - | - |
| Albania | (1,1,0) | 0.515 | 0.162 | - | - | - | - | - | - | - | - | - | - | - | - | - | - | - | - |
| Algeria | (0,2,0) | - | - | - | - | - | - | - | - | - | - | - | - | - | - | - | - | - | - |
| American Samoa | (1,0,1)with non-zero mean | 0.458 | 0.177 | - | - | - | - | - | - | 0.911 | 0.179 | - | - | - | - | - | - | 12.178 | 0.076 |
| Andorra | (0,2,0) | - | - | - | - | - | - | - | - | - | - | - | - | - | - | - | - | - | - |
| Angola | (0,2,1) | - | - | - | - | - | - | - | - | -0.782 | 0.125 | - | - | - | - | - | - | - | - |
| Antigua and Barbuda | (0,2,0) | - | - | - | - | - | - | - | - | - | - | - | - | - | - | - | - | - | - |
| Argentina | (1,1,0)with drift | 0.357 | 0.177 | - | - | - | - | - | - | - | - | - | - | - | - | -0.422 | 0.162 | - | - |
| Armenia | (2,1,0) | 0.523 | 0.176 | 0.345 | 0.176 | - | - | - | - | - | - | - | - | - | - | - | - | - | - |
| Australia | (1,2,0) | -0.666 | 0.146 | - | - | - | - | - | - | - | - | - | - | - | - | - | - | - | - |
| Austria | (0,2,1) | - | - | - | - | - | - | - | - | -0.772 | 0.102 | - | - | - | - | - | - | - | - |
| Azerbaijan | (1,1,0) | 0.736 | 0.119 | - | - | - | - | - | - | - | - | - | - | - | - | - | - | - | - |
| Bahrain | (1,1,0) | 0.688 | 0.133 | - | - | - | - | - | - | - | - | - | - | - | - | - | - | - | - |
| Bangladesh | (0,1,0) | - | - | - | - | - | - | - | - | - | - | - | - | - | - | - | - | - | - |
| Barbados | (0,1,0) | - | - | - | - | - | - | - | - | - | - | - | - | - | - | - | - | - | - |
| Belarus | (0,2,1) | - | - | - | - | - | - | - | - | -0.719 | 0.115 | - | - | - | - | - | - | - | - |
| Belgium | (0,2,1) | - | - | - | - | - | - | - | - | -0.66 | 0.159 | - | - | - | - | - | - | - | - |
| Belize | (0,1,1) | - | - | - | - | - | - | - | - | 0.792 | 0.103 | - | - | - | - | - | - | - | - |
| Benin | (2,0,0)with zero mean | 1.958 | 0.046 | -0.959 | 0.047 | - | - | - | - | - | - | - | - | - | - | - | - | - | - |
| Bermuda | (0,2,1) | - | - | - | - | - | - | - | - | -0.813 | 0.106 | - | - | - | - | - | - | - | - |
| Bhutan | (0,2,1) | - | - | - | - | - | - | - | - | -0.782 | 0.125 | - | - | - | - | - | - | - | - |
| Bolivia | (2,0,0)with non-zero mean | 1.736 | 0.113 | -0.794 | 0.119 | - | - | - | - | - | - | - | - | - | - | - | - | 15.863 | 0.402 |
| Bosnia and Herzegovina | (1,1,0) | 0.594 | 0.154 | - | - | - | - | - | - | - | - | - | - | - | - | - | - | - | - |
| Botswana | (1,1,0) | 0.739 | 0.119 | - | - | - | - | - | - | - | - | - | - | - | - | - | - | - | - |
| Brazil | (0,2,0) | - | - | - | - | - | - | - | - | - | - | - | - | - | - | - | - | - | - |
| Brunei | (0,2,0) | - | - | - | - | - | - | - | - | - | - | - | - | - | - | - | - | - | - |
| Bulgaria | (1,1,0)with drift | 0.513 | 0.163 | - | - | - | - | - | - | - | - | - | - | - | - | -1.071 | 0.53 | - | - |
| Burkina Faso | (1,1,0) | 0.589 | 0.157 | - | - | - | - | - | - | - | - | - | - | - | - | - | - | - | - |
| Burundi | (1,1,0) | 0.838 | 0.09 | - | - | - | - | - | - | - | - | - | - | - | - | - | - | - | - |
| Cambodia | (0,2,0) | - | - | - | - | - | - | - | - | - | - | - | - | - | - | - | - | - | - |
| Cameroon | (0,2,0) | - | - | - | - | - | - | - | - | - | - | - | - | - | - | - | - | - | - |
| Canada | (0,2,1) | - | - | - | - | - | - | - | - | -0.746 | 0.167 | - | - | - | - | - | - | - | - |
| Cape Verde | (1,1,0)with drift | 0.541 | 0.157 | - | - | - | - | - | - | - | - | - | - | - | - | 0.63 | 0.179 | - | - |
| Central African Republic | (1,2,0) | -0.594 | 0.163 | - | - | - | - | - | - | - | - | - | - | - | - | - | - | - | - |
| Chad | (0,2,0) | - | - | - | - | - | - | - | - | - | - | - | - | - | - | - | - | - | - |
| Chile | (0,2,1) | - | - | - | - | - | - | - | - | -0.514 | 0.177 | - | - | - | - | - | - | - | - |
| China | (2,1,0)with drift | 0.769 | 0.169 | -0.511 | 0.162 | - | - | - | - | - | - | - | - | - | - | -1.217 | 0.486 | - | - |
| Colombia | (0,2,2) | - | - | - | - | - | - | - | - | -0.98 | 0.196 | 0.337 | 0.189 | - | - | - | - | - | - |
| Comoros | (0,1,0) | - | - | - | - | - | - | - | - | - | - | - | - | - | - | - | - | - | - |
| Congo | (0,1,0) | - | - | - | - | - | - | - | - | - | - | - | - | - | - | - | - | - | - |
| Costa Rica | (1,0,0)with non-zero mean | 0.789 | 0.11 | - | - | - | - | - | - | - | - | - | - | - | - | - | - | 8.944 | 0.259 |
| Cote d'Ivoire | (2,0,0)with zero mean | 1.95 | 0.048 | -0.951 | 0.048 | - | - | - | - | - | - | - | - | - | - | - | - | - | - |
| Croatia | (0,1,1)with drift | - | - | - | - | - | - | - | - | 0.438 | 0.292 | - | - | - | - | -1.146 | 0.327 | - | - |
| Cuba | (1,1,0) | 0.645 | 0.141 | - | - | - | - | - | - | - | - | - | - | - | - | - | - | - | - |
| Cyprus | (0,1,1) | - | - | - | - | - | - | - | - | 0.562 | 0.165 | - | - | - | - | - | - | - | - |
| Czech Republic | (0,2,1) | - | - | - | - | - | - | - | - | -0.451 | 0.165 | - | - | - | - | - | - | - | - |
| Democratic Republic of the Congo | (0,2,1) | - | - | - | - | - | - | - | - | -0.646 | 0.164 | - | - | - | - | - | - | - | - |
| Denmark | (0,2,0) | - | - | - | - | - | - | - | - | - | - | - | - | - | - | - | - | - | - |
| Djibouti | (2,1,0) | 1.289 | 0.163 | -0.503 | 0.173 | - | - | - | - | - | - | - | - | - | - | - | - | - | - |
| Dominica | (2,0,0)with non-zero mean | 1.446 | 0.147 | -0.582 | 0.147 | - | - | - | - | - | - | - | - | - | - | - | - | 11.248 | 0.169 |
| Dominican Republic | (1,1,0) | 0.691 | 0.13 | - | - | - | - | - | - | - | - | - | - | - | - | - | - | - | - |
| Ecuador | (0,1,0)with drift | - | - | - | - | - | - | - | - | - | - | - | - | - | - | 0.23 | 0.063 | - | - |
| Egypt | (0,1,1) | - | - | - | - | - | - | - | - | 0.395 | 0.196 | - | - | - | - | - | - | - | - |
| El Salvador | (0,1,0) | - | - | - | - | - | - | - | - | - | - | - | - | - | - | - | - | - | - |
| Equatorial Guinea | (0,2,1) | - | - | - | - | - | - | - | - | -0.318 | 0.169 | - | - | - | - | - | - | - | - |
| Eritrea | (0,1,1) | - | - | - | - | - | - | - | - | -0.617 | 0.119 | - | - | - | - | - | - | - | - |
| Estonia | (0,1,1)with drift | - | - | - | - | - | - | - | - | 0.365 | 0.184 | - | - | - | - | -0.741 | 0.306 | - | - |
| Ethiopia | (1,1,0) | 0.926 | 0.053 | - | - | - | - | - | - | - | - | - | - | - | - | - | - | - | - |
| Federated States of Micronesia | (1,1,0) | 0.922 | 0.063 | - | - | - | - | - | - | - | - | - | - | - | - | - | - | - | - |
| Fiji | (0,1,0) | - | - | - | - | - | - | - | - | - | - | - | - | - | - | - | - | - | - |
| Finland | (0,2,1) | - | - | - | - | - | - | - | - | -0.7 | 0.186 | - | - | - | - | - | - | - | - |
| France | (0,2,1) | - | - | - | - | - | - | - | - | -0.512 | 0.16 | - | - | - | - | - | - | - | - |
| Gabon | (1,1,0) | 0.445 | 0.169 | - | - | - | - | - | - | - | - | - | - | - | - | - | - | - | - |
| Georgia | (0,1,1) | - | - | - | - | - | - | - | - | 0.471 | 0.151 | - | - | - | - | - | - | - | - |
| Germany | (0,2,1) | - | - | - | - | - | - | - | - | -0.496 | 0.231 | - | - | - | - | - | - | - | - |
| Ghana | (1,1,0) | 0.522 | 0.157 | - | - | - | - | - | - | - | - | - | - | - | - | - | - | - | - |
| Greece | (0,2,0) | - | - | - | - | - | - | - | - | - | - | - | - | - | - | - | - | - | - |
| Greenland | (2,0,0)with non-zero mean | 1.276 | 0.175 | -0.359 | 0.184 | - | - | - | - | - | - | - | - | - | - | - | - | 75.191 | 1.745 |
| Grenada | (0,2,1) | - | - | - | - | - | - | - | - | -0.734 | 0.131 | - | - | - | - | - | - | - | - |
| Guam | (0,1,1) | - | - | - | - | - | - | - | - | 0.781 | 0.124 | - | - | - | - | - | - | - | - |
| Guatemala | (1,1,0) | 0.563 | 0.158 | - | - | - | - | - | - | - | - | - | - | - | - | - | - | - | - |
| Guinea | (0,2,0) | - | - | - | - | - | - | - | - | - | - | - | - | - | - | - | - | - | - |
| Guinea-Bissau | (0,2,0) | - | - | - | - | - | - | - | - | - | - | - | - | - | - | - | - | - | - |
| Guyana | (2,0,0)with non-zero mean | 1.021 | 0.17 | -0.399 | 0.169 | - | - | - | - | - | - | - | - | - | - | - | - | 51.224 | 0.601 |
| Haiti | (1,1,0)with drift | 0.335 | 0.178 | - | - | - | - | - | - | - | - | - | - | - | - | -0.047 | 0.024 | - | - |
| Honduras | (0,2,2) | - | - | - | - | - | - | - | - | -1.009 | 0.468 | 0.968 | 0.875 | - | - | - | - | - | - |
| Hungary | (0,2,1) | - | - | - | - | - | - | - | - | -0.745 | 0.1 | - | - | - | - | - | - | - | - |
| Iceland | (0,1,1)with drift | - | - | - | - | - | - | - | - | 0.626 | 0.196 | - | - | - | - | -0.348 | 0.172 | - | - |
| India | (0,1,0) | - | - | - | - | - | - | - | - | - | - | - | - | - | - | - | - | - | - |
| Indonesia | (0,1,1)with drift | - | - | - | - | - | - | - | - | 0.495 | 0.152 | - | - | - | - | 0.031 | 0.01 | - | - |
| Iran | (2,0,0)with non-zero mean | 1.723 | 0.095 | -0.85 | 0.098 | - | - | - | - | - | - | - | - | - | - | - | - | 9.274 | 0.107 |
| Iraq | (0,2,1) | - | - | - | - | - | - | - | - | -0.591 | 0.163 | - | - | - | - | - | - | - | - |
| Ireland | (0,1,0)with drift | - | - | - | - | - | - | - | - | - | - | - | - | - | - | -0.098 | 0.036 | - | - |
| Israel | (1,1,0) | 0.565 | 0.172 | - | - | - | - | - | - | - | - | - | - | - | - | - | - | - | - |
| Italy | (0,2,1) | - | - | - | - | - | - | - | - | -0.787 | 0.115 | - | - | - | - | - | - | - | - |
| Jamaica | (0,1,0)with drift | - | - | - | - | - | - | - | - | - | - | - | - | - | - | 0.094 | 0.035 | - | - |
| Japan | (0,2,1) | - | - | - | - | - | - | - | - | -0.644 | 0.149 | - | - | - | - | - | - | - | - |
| Jordan | (1,1,0) | 0.659 | 0.135 | - | - | - | - | - | - | - | - | - | - | - | - | - | - | - | - |
| Kazakhstan | (1,0,2)with non-zero mean | 0.658 | 0.166 | - | - | - | - | - | - | 0.534 | 0.153 | 0.676 | 0.216 | - | - | - | - | 40.122 | 0.957 |
| Kenya | (1,2,0) | -0.438 | 0.171 | - | - | - | - | - | - | - | - | - | - | - | - | - | - | - | - |
| Kiribati | (0,1,0) | - | - | - | - | - | - | - | - | - | - | - | - | - | - | - | - | - | - |
| Kuwait | (0,1,0) | - | - | - | - | - | - | - | - | - | - | - | - | - | - | - | - | - | - |
| Kyrgyzstan | (0,1,0)with drift | - | - | - | - | - | - | - | - | - | - | - | - | - | - | -0.406 | 0.088 | - | - |
| Laos | (1,1,0) | 0.944 | 0.043 | - | - | - | - | - | - | - | - | - | - | - | - | - | - | - | - |
| Latvia | (2,1,0)with drift | 0.712 | 0.156 | -0.566 | 0.156 | - | - | - | - | - | - | - | - | - | - | -0.891 | 0.309 | - | - |
| Lebanon | (0,1,2) | - | - | - | - | - | - | - | - | 1.182 | 0.348 | 0.816 | 0.627 | - | - | - | - | - | - |
| Lesotho | (1,1,0) | 0.575 | 0.162 | - | - | - | - | - | - | - | - | - | - | - | - | - | - | - | - |
| Liberia | (0,2,1) | - | - | - | - | - | - | - | - | -0.604 | 0.177 | - | - | - | - | - | - | - | - |
| Libya | (0,1,1) | - | - | - | - | - | - | - | - | 0.795 | 0.102 | - | - | - | - | - | - | - | - |
| Lithuania | (1,1,0) | 0.467 | 0.169 | - | - | - | - | - | - | - | - | - | - | - | - | - | - | - | - |
| Luxembourg | (1,1,0)with drift | 0.406 | 0.172 | - | - | - | - | - | - | - | - | - | - | - | - | -0.397 | 0.143 | - | - |
| Macedonia | (0,1,1)with drift | - | - | - | - | - | - | - | - | 0.61 | 0.18 | - | - | - | - | -0.28 | 0.13 | - | - |
| Madagascar | (0,1,0)with drift | - | - | - | - | - | - | - | - | - | - | - | - | - | - | -0.487 | 0.198 | - | - |
| Malawi | (0,2,0) | - | - | - | - | - | - | - | - | - | - | - | - | - | - | - | - | - | - |
| Malaysia | (0,1,1) | - | - | - | - | - | - | - | - | 0.583 | 0.154 | - | - | - | - | - | - | - | - |
| Maldives | (0,1,1)with drift | - | - | - | - | - | - | - | - | 0.627 | 0.303 | - | - | - | - | -0.338 | 0.132 | - | - |
| Mali | (4,0,0)with non-zero mean | 1.179 | 0.177 | -0.104 | 0.298 | 0.025 | 0.298 | -0.353 | 0.182 | - | - | - | - | - | - | - | - | 32.138 | 0.088 |
| Malta | (0,1,0)with drift | - | - | - | - | - | - | - | - | - | - | - | - | - | - | -0.11 | 0.041 | - | - |
| Marshall Islands | (2,2,0) | -1.242 | 0.186 | -0.679 | 0.173 | - | - | - | - | - | - | - | - | - | - | - | - | - | - |
| Mauritania | (2,0,2)with non-zero mean | 1.865 | 0.041 | -0.968 | 0.036 | - | - | - | - | -0.451 | 0.252 | -0.074 | 0.3 | - | - | - | - | 35.076 | 0.194 |
| Mauritius | (2,1,1)with drift | -0.479 | 0.187 | -0.368 | 0.183 | - | - | - | - | 0.911 | 0.122 | - | - | - | - | -0.215 | 0.052 | - | - |
| Mexico | (2,0,0)with non-zero mean | 1.44 | 0.154 | -0.569 | 0.165 | - | - | - | - | - | - | - | - | - | - | - | - | 8.478 | 0.238 |
| Moldova | (1,1,0) | 0.459 | 0.176 | - | - | - | - | - | - | - | - | - | - | - | - | - | - | - | - |
| Mongolia | (0,2,0) | - | - | - | - | - | - | - | - | - | - | - | - | - | - | - | - | - | - |
| Montenegro | (1,1,0) | 0.505 | 0.164 | - | - | - | - | - | - | - | - | - | - | - | - | - | - | - | - |
| Morocco | (2,0,0)with non-zero mean | 1.818 | 0.076 | -0.876 | 0.082 | - | - | - | - | - | - | - | - | - | - | - | - | 14.054 | 0.557 |
| Mozambique | (1,1,0) | 0.524 | 0.187 | - | - | - | - | - | - | - | - | - | - | - | - | - | - | - | - |
| Myanmar | (0,2,0) | - | - | - | - | - | - | - | - | - | - | - | - | - | - | - | - | - | - |
| Namibia | (0,2,0) | - | - | - | - | - | - | - | - | - | - | - | - | - | - | - | - | - | - |
| Nepal | (1,1,0) | 0.856 | 0.084 | - | - | - | - | - | - | - | - | - | - | - | - | - | - | - | - |
| Netherlands | (0,2,1) | - | - | - | - | - | - | - | - | -0.603 | 0.162 | - | - | - | - | - | - | - | - |
| New Zealand | (0,1,0)with drift | - | - | - | - | - | - | - | - | - | - | - | - | - | - | -0.154 | 0.066 | - | - |
| Nicaragua | (0,2,0) | - | - | - | - | - | - | - | - | - | - | - | - | - | - | - | - | - | - |
| Niger | (3,0,0)with non-zero mean | 1.464 | 0.169 | -0.127 | 0.33 | -0.425 | 0.174 | - | - | - | - | - | - | - | - | - | - | 37.142 | 0.447 |
| Nigeria | (1,1,0) | 0.774 | 0.11 | - | - | - | - | - | - | - | - | - | - | - | - | - | - | - | - |
| North Korea | (0,2,0) | - | - | - | - | - | - | - | - | - | - | - | - | - | - | - | - | - | - |
| Northern Mariana Islands | (0,1,0)with drift | - | - | - | - | - | - | - | - | - | - | - | - | - | - | 0.165 | 0.084 | - | - |
| Norway | (0,2,1) | - | - | - | - | - | - | - | - | -0.823 | 0.122 | - | - | - | - | - | - | - | - |
| Oman | (2,0,1)with non-zero mean | 1.71 | 0.079 | -0.93 | 0.06 | - | - | - | - | -0.721 | 0.223 | - | - | - | - | - | - | 7.512 | 0.043 |
| Pakistan | (2,0,0)with non-zero mean | 1.8 | 0.103 | -0.831 | 0.105 | - | - | - | - | - | - | - | - | - | - | - | - | 5.184 | 0.375 |
| Palestine | (0,1,0)with drift | - | - | - | - | - | - | - | - | - | - | - | - | - | - | -0.041 | 0.023 | - | - |
| Panama | (0,2,1) | - | - | - | - | - | - | - | - | -0.687 | 0.128 | - | - | - | - | - | - | - | - |
| Papua New Guinea | (1,1,0)with drift | 0.686 | 0.142 | - | - | - | - | - | - | - | - | - | - | - | - | -0.088 | 0.042 | - | - |
| Paraguay | (0,1,0)with drift | - | - | - | - | - | - | - | - | - | - | - | - | - | - | 0.076 | 0.045 | - | - |
| Peru | (0,1,1) | - | - | - | - | - | - | - | - | 0.784 | 0.126 | - | - | - | - | - | - | - | - |
| Philippines | (0,2,1) | - | - | - | - | - | - | - | - | -0.474 | 0.192 | - | - | - | - | - | - | - | - |
| Poland | (2,0,0)with non-zero mean | 1.362 | 0.18 | -0.409 | 0.181 | - | - | - | - | - | - | - | - | - | - | - | - | 17.207 | 1.182 |
| Portugal | (0,1,0)with drift | - | - | - | - | - | - | - | - | - | - | - | - | - | - | -0.457 | 0.203 | - | - |
| Puerto Rico | (0,2,2) | - | - | - | - | - | - | - | - | -1.244 | 0.192 | 0.468 | 0.187 | - | - | - | - | - | - |
| Qatar | (0,1,1) | - | - | - | - | - | - | - | - | 0.975 | 0.315 | - | - | - | - | - | - | - | - |
| Romania | (0,1,0) | - | - | - | - | - | - | - | - | - | - | - | - | - | - | - | - | - | - |
| Russian Federation | (0,1,1) | - | - | - | - | - | - | - | - | 0.454 | 0.184 | - | - | - | - | - | - | - | - |
| Rwanda | (2,1,0) | 1.555 | 0.148 | -0.601 | 0.148 | - | - | - | - | - | - | - | - | - | - | - | - | - | - |
| Saint Lucia | (0,1,1) | - | - | - | - | - | - | - | - | 0.614 | 0.155 | - | - | - | - | - | - | - | - |
| Saint Vincent and the Grenadines | (2,0,1)with non-zero mean | 1.777 | 0.088 | -0.901 | 0.078 | - | - | - | - | -0.605 | 0.227 | - | - | - | - | - | - | 16.919 | 0.163 |
| Samoa | (0,2,1) | - | - | - | - | - | - | - | - | -0.521 | 0.155 | - | - | - | - | - | - | - | - |
| Sao Tome and Principe | (0,1,1)with drift | - | - | - | - | - | - | - | - | 0.373 | 0.181 | - | - | - | - | 0.127 | 0.021 | - | - |
| Saudi Arabia | (1,1,0) | 0.825 | 0.097 | - | - | - | - | - | - | - | - | - | - | - | - | - | - | - | - |
| Senegal | (0,1,0) | - | - | - | - | - | - | - | - | - | - | - | - | - | - | - | - | - | - |
| Serbia | (0,1,0)with drift | - | - | - | - | - | - | - | - | - | - | - | - | - | - | -0.495 | 0.251 | - | - |
| Seychelles | (0,2,0) | - | - | - | - | - | - | - | - | - | - | - | - | - | - | - | - | - | - |
| Sierra Leone | (0,2,0) | - | - | - | - | - | - | - | - | - | - | - | - | - | - | - | - | - | - |
| Singapore | (0,1,0)with drift | - | - | - | - | - | - | - | - | - | - | - | - | - | - | -1.166 | 0.179 | - | - |
| Slovakia | (0,1,0)with drift | - | - | - | - | - | - | - | - | - | - | - | - | - | - | -0.29 | 0.063 | - | - |
| Slovenia | (0,1,0)with drift | - | - | - | - | - | - | - | - | - | - | - | - | - | - | -0.861 | 0.27 | - | - |
| Solomon Islands | (0,2,0) | - | - | - | - | - | - | - | - | - | - | - | - | - | - | - | - | - | - |
| Somalia | (2,1,0) | 1.345 | 0.169 | -0.442 | 0.172 | - | - | - | - | - | - | - | - | - | - | - | - | - | - |
| South Africa | (2,0,0)with non-zero mean | 1.588 | 0.14 | -0.66 | 0.146 | - | - | - | - | - | - | - | - | - | - | - | - | 21.619 | 1.929 |
| South Korea | (0,2,0) | - | - | - | - | - | - | - | - | - | - | - | - | - | - | - | - | - | - |
| South Sudan | (0,2,0) | - | - | - | - | - | - | - | - | - | - | - | - | - | - | - | - | - | - |
| Spain | (0,1,0)with drift | - | - | - | - | - | - | - | - | - | - | - | - | - | - | -0.343 | 0.064 | - | - |
| Sri Lanka | (1,1,0) | 0.653 | 0.138 | - | - | - | - | - | - | - | - | - | - | - | - | - | - | - | - |
| Sudan | (0,2,0) | - | - | - | - | - | - | - | - | - | - | - | - | - | - | - | - | - | - |
| Suriname | (0,1,1) | - | - | - | - | - | - | - | - | 0.459 | 0.17 | - | - | - | - | - | - | - | - |
| Swaziland | (2,0,3)with non-zero mean | 1.916 | 0.453 | -0.95 | 0.574 | - | - | - | - | -0.239 | 1.957 | -0.211 | 1.146 | -0.017 | 0.293 | - | - | 35.652 | 4.431 |
| Sweden | (0,1,0)with drift | - | - | - | - | - | - | - | - | - | - | - | - | - | - | -0.478 | 0.088 | - | - |
| Switzerland | (1,1,0)with drift | 0.442 | 0.172 | - | - | - | - | - | - | - | - | - | - | - | - | -0.752 | 0.292 | - | - |
| Syria | (1,1,0) | 0.525 | 0.157 | - | - | - | - | - | - | - | - | - | - | - | - | - | - | - | - |
| Taiwan | (2,0,0)with non-zero mean | 1.612 | 0.13 | -0.716 | 0.132 | - | - | - | - | - | - | - | - | - | - | - | - | 44.819 | 1.581 |
| Tajikistan | (1,2,0) | -0.443 | 0.172 | - | - | - | - | - | - | - | - | - | - | - | - | - | - | - | - |
| Tanzania | (1,1,0) | 0.846 | 0.088 | - | - | - | - | - | - | - | - | - | - | - | - | - | - | - | - |
| Thailand | (0,1,0)with drift | - | - | - | - | - | - | - | - | - | - | - | - | - | - | -0.207 | 0.088 | - | - |
| The Bahamas | (0,1,0) | - | - | - | - | - | - | - | - | - | - | - | - | - | - | - | - | - | - |
| The Gambia | (0,2,0) | - | - | - | - | - | - | - | - | - | - | - | - | - | - | - | - | - | - |
| Timor-Leste | (0,2,0) | - | - | - | - | - | - | - | - | - | - | - | - | - | - | - | - | - | - |
| Togo | (2,0,0)with non-zero mean | 1.839 | 0.078 | -0.876 | 0.081 | - | - | - | - | - | - | - | - | - | - | - | - | 53.786 | 2.978 |
| Tonga | (2,0,0)with non-zero mean | 1.224 | 0.177 | -0.332 | 0.182 | - | - | - | - | - | - | - | - | - | - | - | - | 13.022 | 0.083 |
| Trinidad and Tobago | (0,1,0)with drift | - | - | - | - | - | - | - | - | - | - | - | - | - | - | -0.174 | 0.109 | - | - |
| Tunisia | (0,2,0) | - | - | - | - | - | - | - | - | - | - | - | - | - | - | - | - | - | - |
| Turkey | (0,1,1)with drift | - | - | - | - | - | - | - | - | 0.889 | 0.115 | - | - | - | - | -0.267 | 0.093 | - | - |
| Turkmenistan | (2,0,0)with non-zero mean | 1.556 | 0.124 | -0.71 | 0.127 | - | - | - | - | - | - | - | - | - | - | - | - | 11.688 | 0.362 |
| Uganda | (0,2,0) | - | - | - | - | - | - | - | - | - | - | - | - | - | - | - | - | - | - |
| Ukraine | (0,1,0) | - | - | - | - | - | - | - | - | - | - | - | - | - | - | - | - | - | - |
| United Arab Emirates | (0,1,0) | - | - | - | - | - | - | - | - | - | - | - | - | - | - | - | - | - | - |
| United Kingdom | (0,2,1) | - | - | - | - | - | - | - | - | -0.666 | 0.122 | - | - | - | - | - | - | - | - |
| United States | (0,2,1) | - | - | - | - | - | - | - | - | -0.537 | 0.153 | - | - | - | - | - | - | - | - |
| Uruguay | (0,2,1) | - | - | - | - | - | - | - | - | -0.771 | 0.154 | - | - | - | - | - | - | - | - |
| Uzbekistan | (2,0,0)with non-zero mean | 1.429 | 0.146 | -0.571 | 0.149 | - | - | - | - | - | - | - | - | - | - | - | - | 12.154 | 0.432 |
| Vanuatu | (0,2,0) | - | - | - | - | - | - | - | - | - | - | - | - | - | - | - | - | - | - |
| Venezuela | (1,1,0) | 0.395 | 0.172 | - | - | - | - | - | - | - | - | - | - | - | - | - | - | - | - |
| Vietnam | (0,2,0) | - | - | - | - | - | - | - | - | - | - | - | - | - | - | - | - | - | - |
| Virgin Islands, U.S. | (1,0,0)with non-zero mean | 0.919 | 0.062 | - | - | - | - | - | - | - | - | - | - | - | - | - | - | 20.8 | 0.488 |
| Yemen | (0,2,0) | - | - | - | - | - | - | - | - | - | - | - | - | - | - | - | - | - | - |
| Zambia | (1,1,0) | 0.938 | 0.056 | - | - | - | - | - | - | - | - | - | - | - | - | - | - | - | - |
| Zimbabwe | (0,2,0) | - | - | - | - | - | - | - | - | - | - | - | - | - | - | - | - | - | - |

ARIMA = Autoregressive Integrated Moving Average model.
