# Supplementary figures and images for: Time Trends and Predictions of Suicide Mortality for People Aged 70 Years and Over From 1990 to 2030 Based on the Global Burden of Disease Study 2017
Source: Front Psychiatry. 2021 Sep 27;12:721343. doi: 10.3389/fpsyt.2021.721343 (PMC8502866; doi:10.3389/fpsyt.2021.721343)

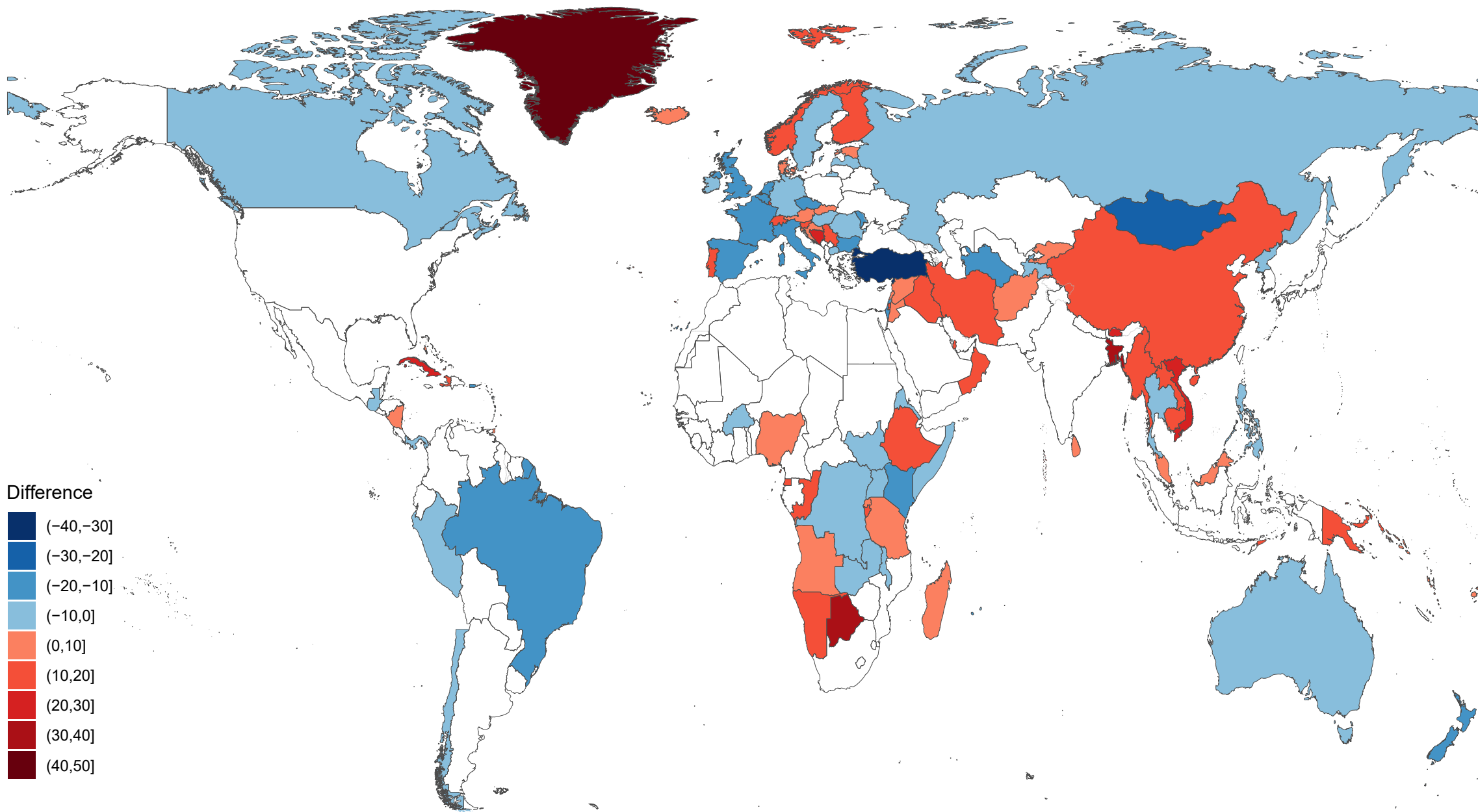

Supplement: Supplementary S1 — Partial statistical methods used in the study. [file Data_Sheet_1.zip › Supplementary Figure 1.pdf]

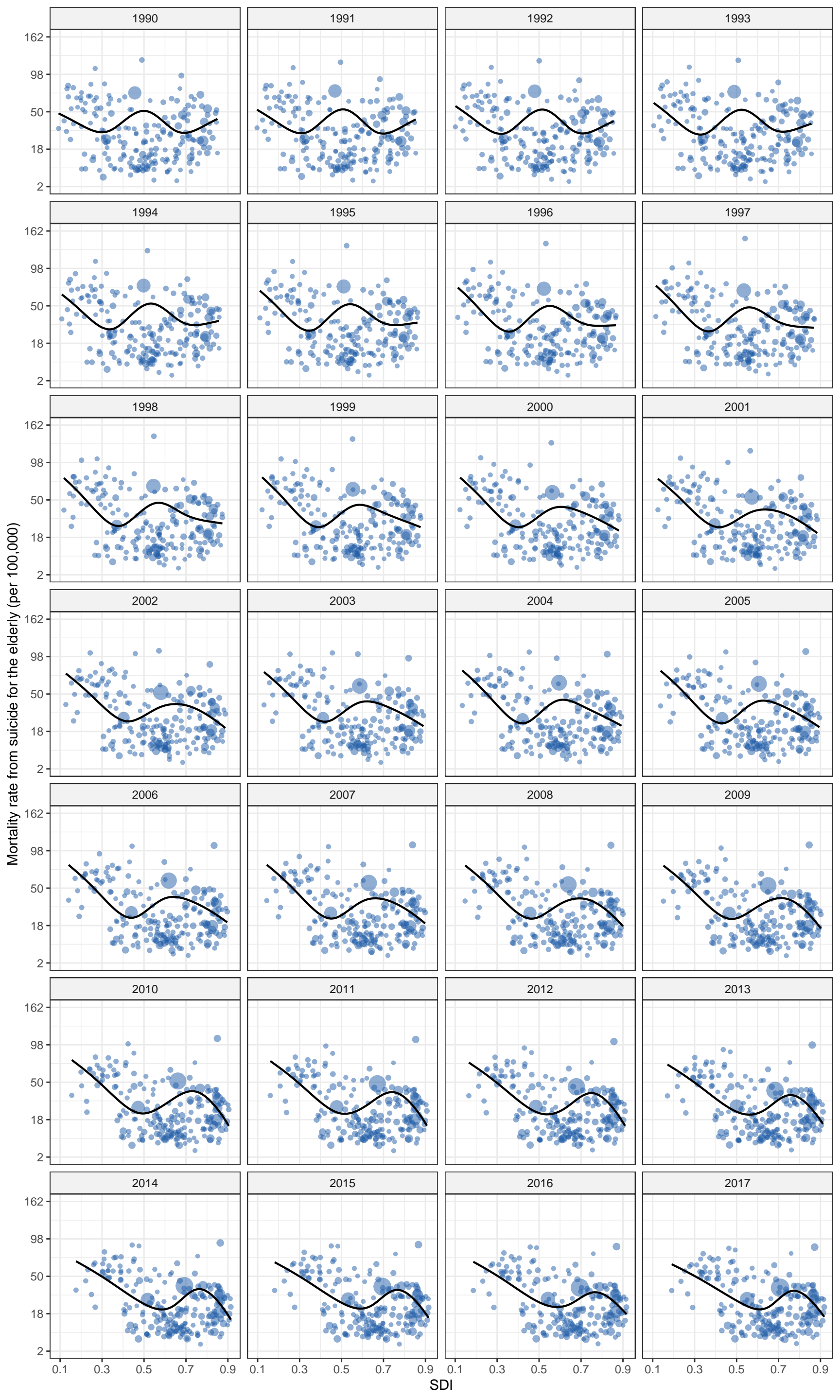

Supplement: Supplementary S1 — Partial statistical methods used in the study. [file Data_Sheet_1.zip › Supplementary Figure 2.pdf]

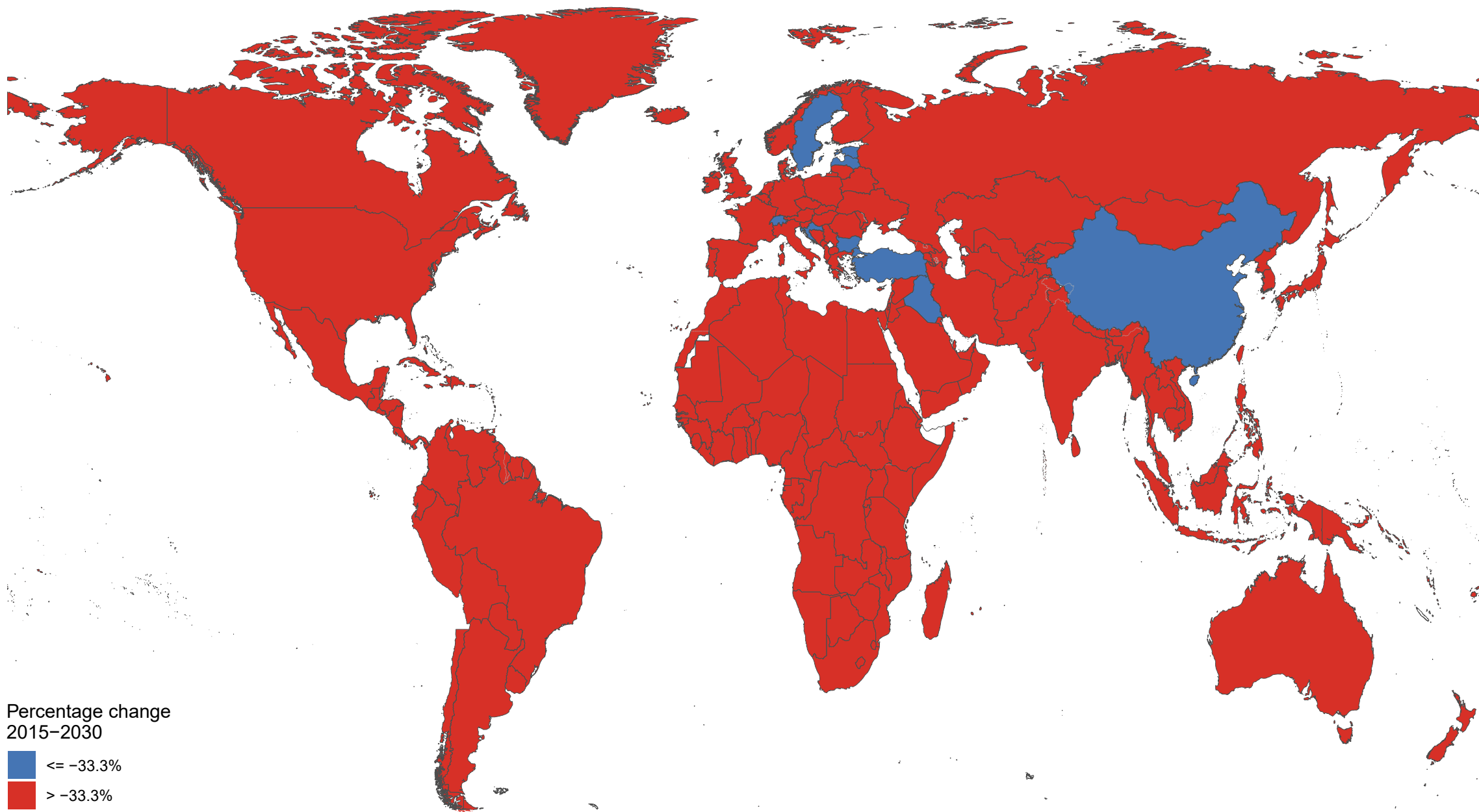

Percentage change  
2015-2030

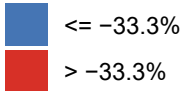

Supplement: Supplementary S1 — Partial statistical methods used in the study. [file Data_Sheet_1.zip › Supplementary Figure 3.pdf]

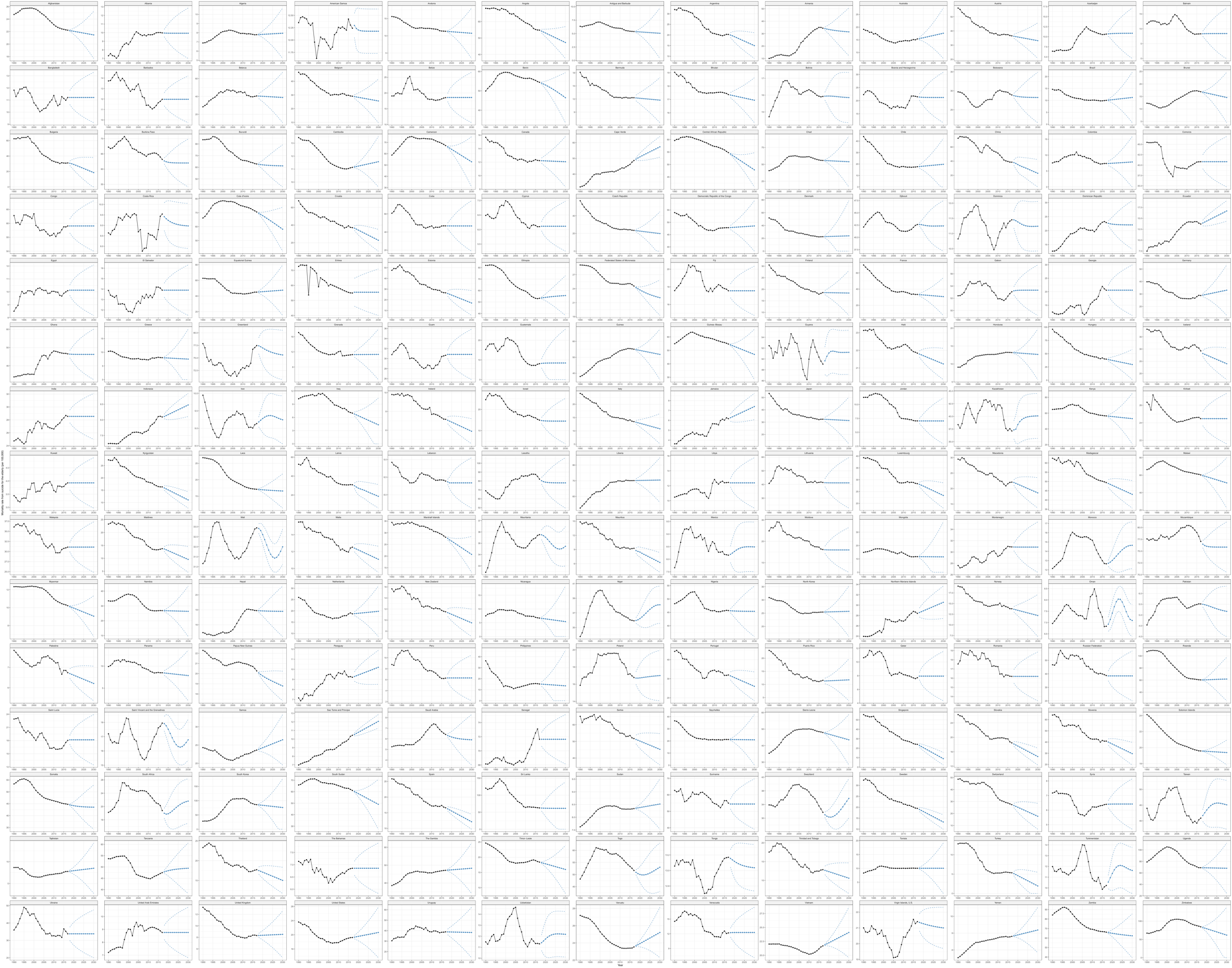

Supplement: Supplementary S1 — Partial statistical methods used in the study. [file Data_Sheet_1.zip › Supplementary Figure 4.pdf]

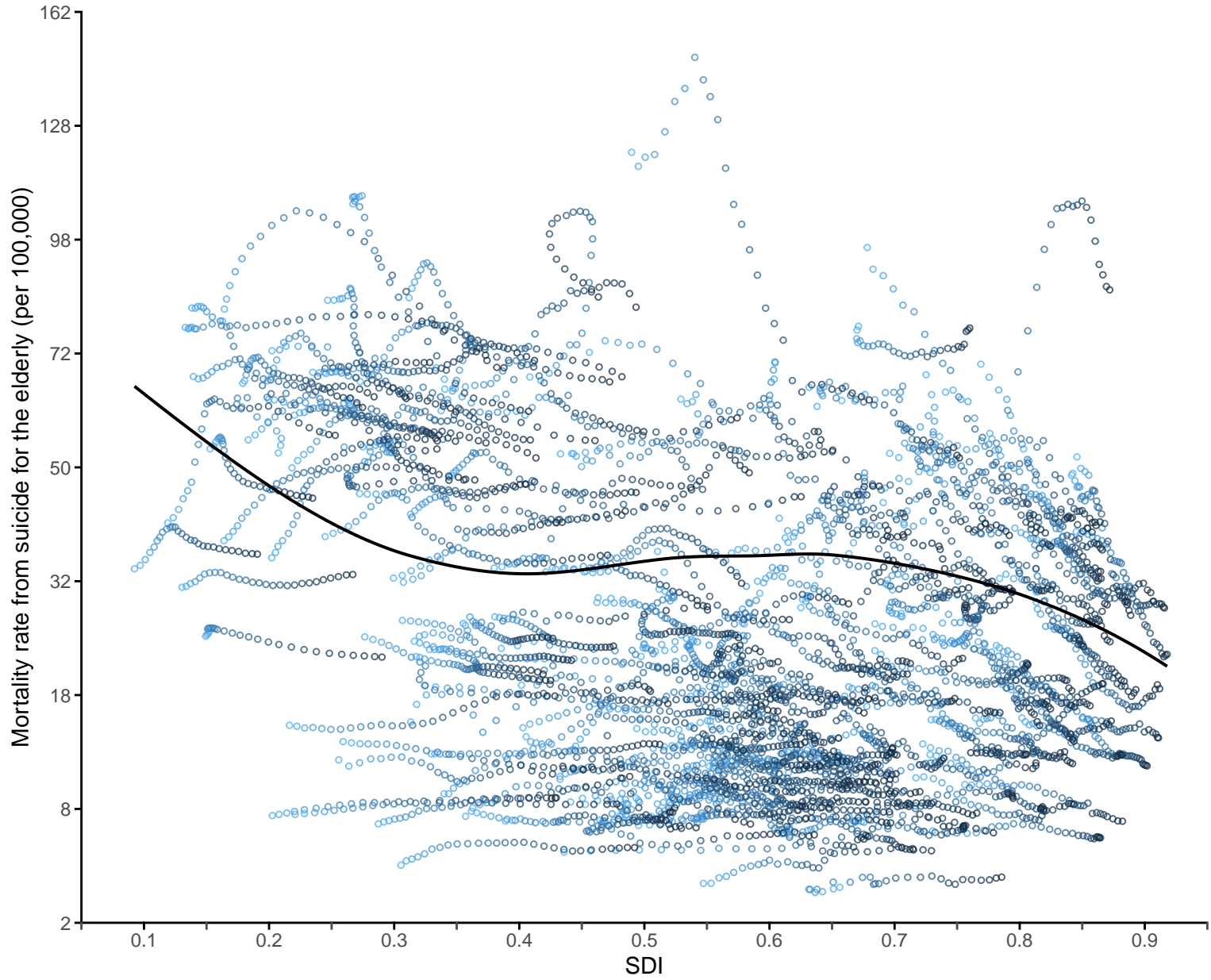

Supplement: Supplementary S1 — Partial statistical methods used in the study. [file Data_Sheet_1.zip › Supplementary Figure 5.pdf]

A

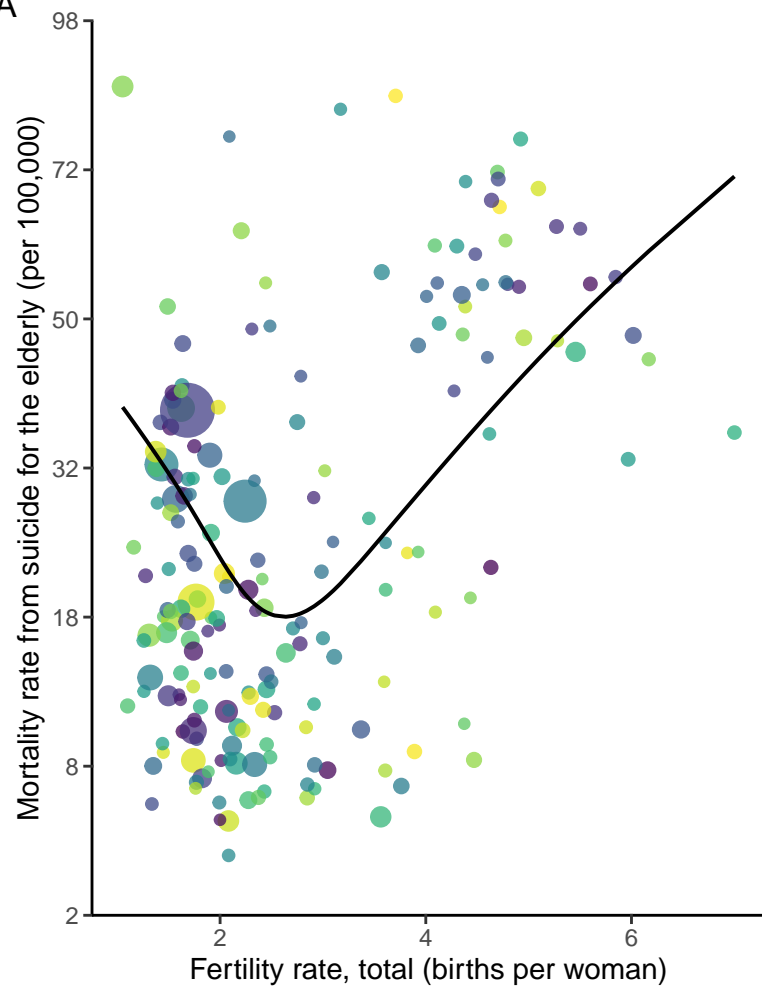

B

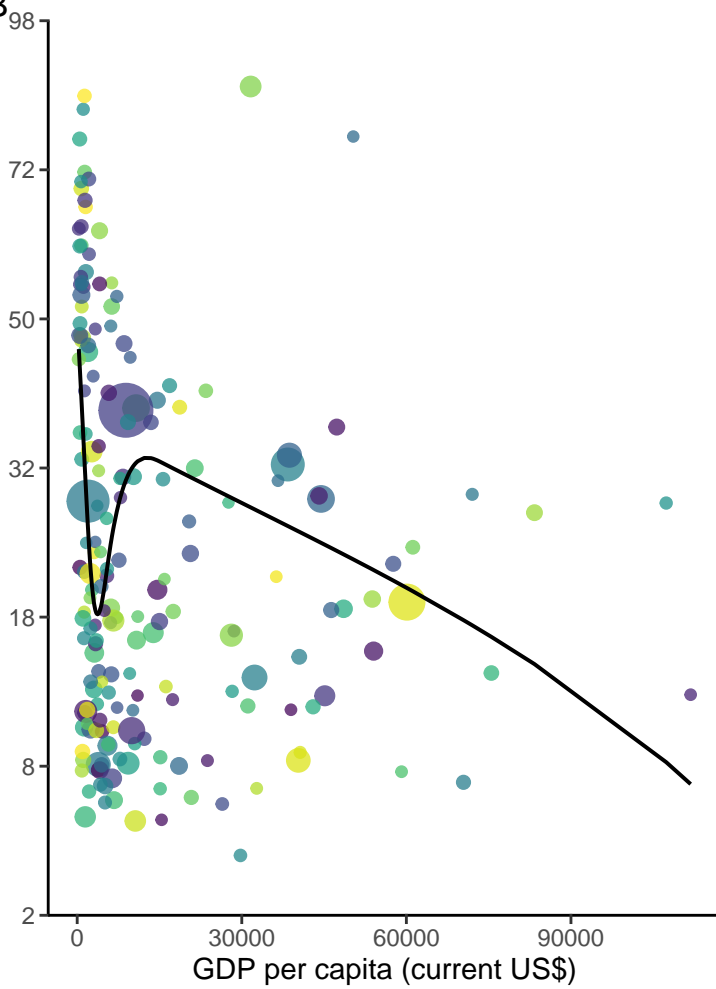

C

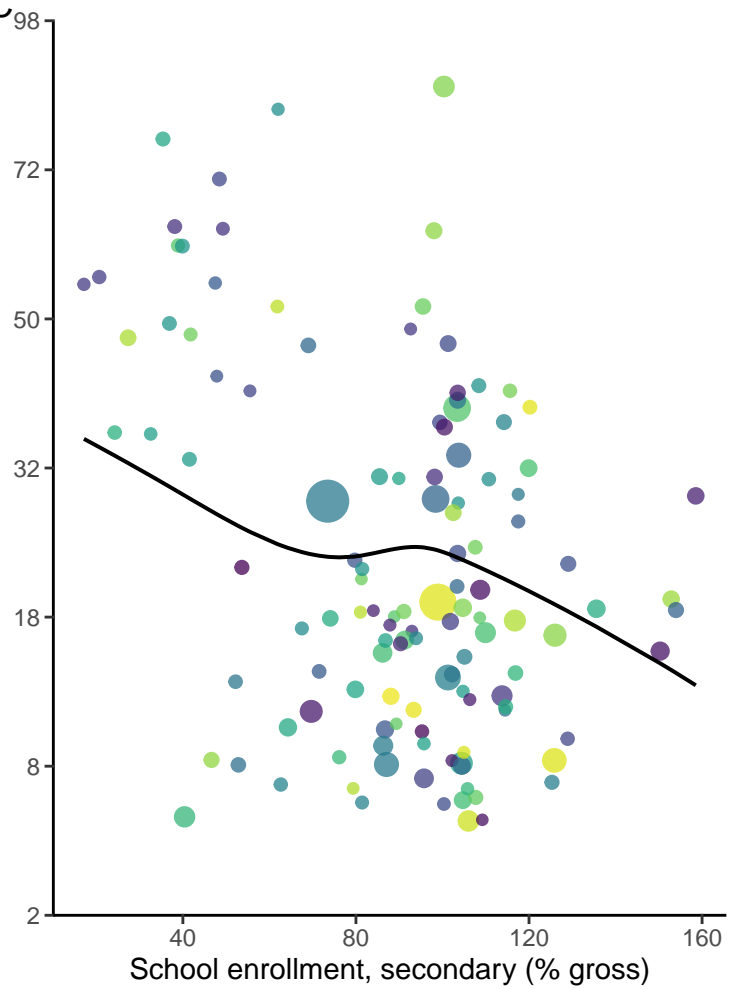

Supplement: Supplementary S1 — Partial statistical methods used in the study. [file Data_Sheet_1.zip › Supplementary Figure 6.pdf]
